# Investigations into the biosynthesis of the antifungal strobilurins†
†Electronic supplementary information (ESI) available. See DOI: 10.1039/c8ob00608c


**DOI:** 10.1039/c8ob00608c

**Published:** 2018-07-20

**Authors:** Zafar Iqbal, Li-Chen Han, Anna M. Soares-Sello, Risa Nofiani, Gerald Thormann, Axel Zeeck, Russell J. Cox, Christine L. Willis, Thomas J. Simpson

**Affiliations:** a School of Chemistry , University of Bristol , Cantocks Close , Bristol , BS8 1TS , UK . Email: tom.simpson@bristol.ac.uk ; Email: chris.willis@bristol.ac.uk; b Institut für Organische und Biomolekulare Chemie , Georg-August Universität , Tammannstraße 2 , 37077 Göttingen , Germany; c Institut für Organische Chemie Chemistry , Schneiderberg 1B, Leibniz Universität , 30167 Hannover , Germany

## Abstract

The strobilurins are important antifungal metabolites isolated from a number of basidiomycetes and have been valuable leads for the development of commercially important fungicides. Isotopic labelling studies with early and advanced intermediates confirm for the first time that they are produced *via* a linear tetraketide, primed with the rare benzoate starter unit, itself derived from phenylalanine *via* cinnamate. Isolation of a novel biphenyl metabolite, pseudostrobilurin B, provides evidence for the involvement of an epoxide in the key rearrangement to form the β-methoxyacrylate moiety essential for biological activity. Formation of two bolineol related metabolites, strobilurins Y and Z, also probably involves epoxide intermediates. Time course studies indicate a likely biosynthetic pathway from strobilurin A, with the simplest non-subsubstituted benzoate ring, to strobilurin G with a complex dioxepin terpenoid-derived substituent. Precursor-directed biosynthetic studies allow production of a number of novel ring-halogenated analogues as well as a new pyridyl strobilurin. These studies also provide evidence for a non-linear biosynthetic relationship between strobilurin A and strobilurin B.

## Abstract 2

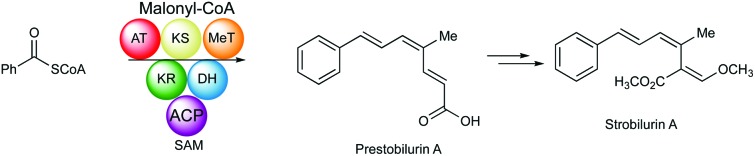
Prestrobilurin A, formed *via* benzoate and cinnamate, has been identified as the first enzyme free intermediate in strobilurin biosynthesis.

## Introduction

Strobilurins are a group of bioactive metabolites produced by various fungi.^1^ Mucidin **1** was the first to be isolated in 1965 from the basidiomycete *Oudemansiella mucida*.^2^ The triene system was initially assigned the all *E*,*E*,*E* configuration, and its potent antifungal activity led to its commercialisation as “mucidermin” for the treatment of skin infections.^3^ In 1977, Anke and co-workers isolated two antifungal metabolites, strobilurin A **2** and strobilurin B **3** from *Strobilurus tenacellus*.^4^ The former was clearly identical to mucidin which was thus re-assigned the *E*,*Z*,*E* configuration. Many other strobilurin analogues have been isolated from other basidiomycetes. Structural variations include oudemansin A **4** from *Oudemansiella mucida* in which the central 9/10 double bond of the triene system has formally had methanol added,^5^ 9-methoxystrobilurin A **5** from *Flaviolaschia* sp. which contains a methyl enol ether^6^ and 14-hydroxystrobilurin A **6** from a *Petrula* sp. containing a hydroxymethyl.^7^ A number of strobilurins have complex dioxepin substituents containing two highly modified prenyl moieties on the phenyl ring, *e.g.* strobilurin G **7** from *Bolinea lutea*.^8^ Bolineol **8** in which the methoxyacrylate substituent of strobilurin A has been replaced by a methyl 3-hydroxy-propionate moiety has also been isolated from *B. lutea*.^9^


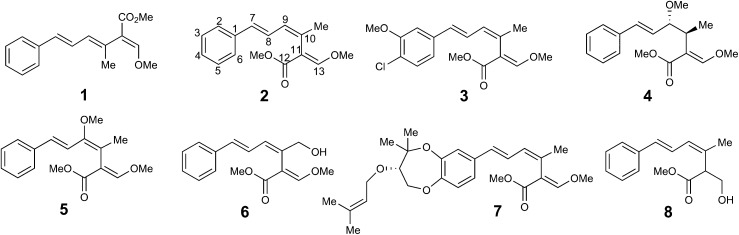
The key β-methoxyacrylate toxophore targets the Qo site of complex III of the mitochondrial electron transport chain and prevents ATP synthesis.^10^ The major class of β-methoxyacrylate agricultural fungicides were developed from the structures of **2** and **3** with the aim of increasing photo-stability and selectivity. Thus, compounds such as azoxystrobin **9** (Syngenta) and Kresoxim-methyl **10** (BASF) are among the most widely used fungicides worldwide, used as effective treatments against a broad range of destructive fungal plant pathogens.^11^,^12^ The strobilurin fungicides are estimated to have been worth $3.4 billion per annum in 2015 and make up 25% of the fungicide market and 6.7% of the total crop protection market.^13^ Other antifungal natural products containing the β-methoxyacrylate toxophore include myxothiazole **11** and cyrmenin A **12** from the myxobacteria, *Myxococcus fulvus* and *Cystobacter armeniaca* respectively.^14^,^15^


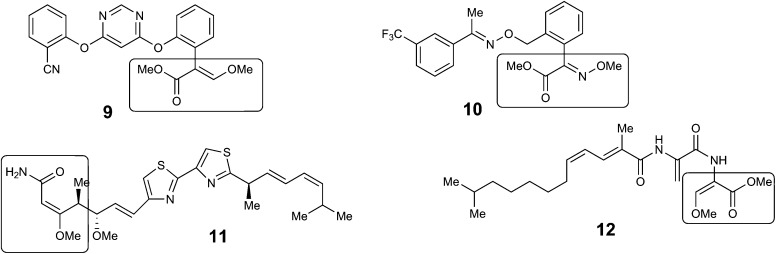
The biosynthesis of the strobilurins, particularly that of the β-methoxyacrylate toxophore, remains obscure despite the fact that the strobilurins and analogues are amongst the most commercially important fungal metabolites known. Early labelling studies on mucidin were consistent with a polyketide produced from a benzoate starter, itself derived *via* phenylalanine, extended by successive condensations with three malonates, and a *C*-methylation from *S*-adenosyl methionine (SAM).^16^ The labelling of both C-11 and C-12 from [1-^13^C]-acetate suggested that rearrangement of an originally linear polyketide chain occurs. *B. lutea* has been described as the only ascomycete to produce the strobilurin/oudemansin family of metabolites.^17^

## Results and discussion

**Scheme 1:**
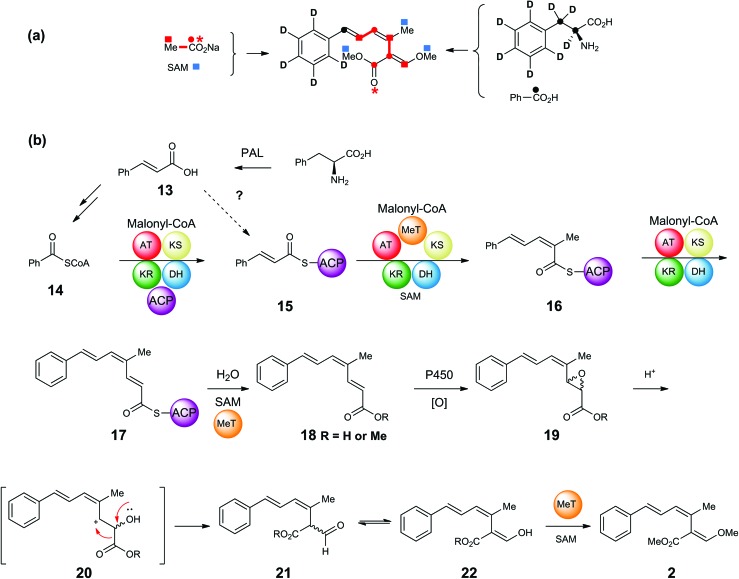
(a) Incorporation of ^2^H and ^13^C-labelled precursors into strobilurin A **2** in *S. tenacellus* and *B. lutea*. (b) Proposed assembly and rearrangement of strobilurin tetraketide.

Feeding studies using singly and doubly labelled [^13^C]-acetates, [*methyl*-^13^C]-methionine, [^2^H_8_]-, [3-^13^C]- and [2,3-^13^C_2_]-phenylalanines, supplemented to cultures of *S. tenacellus* and *B. lutea* resulted in the production of isotopically enriched strobilurin A **2** (Scheme 1a). The results are consistent with the pathway summarised in Scheme 1b, in which phenylalanine undergoes loss of ammonia to give cinnamic acid **13**,^18^,^19^ followed by degradation to benzoate. Although benzoate is a relatively uncommon starter unit in polyketide biosynthesis, benzoyl CoA serves as a component in the formation of a number of important plant metabolites including taxol^20^ and cocaine.^21^ The hexaketide moiety in squalestatin (zaragozic acid) biosynthesis in fungi is also formed from a benzoate starter, now known to be produced from phenylalanine *via* cinnamic acid **13**.^22^,^23^ The bacterial metabolites enterocin (*Streptomyces maritimus*)^24^ and soraphen A (*Sorangium cellulosum*)^25^ also have benzoate priming their polyketide synthases.

In strobilurin A **2**, the labelling pattern supports a pathway in which benzoyl CoA **14** would undergo three successive chain elongations with one *C*-methylation *via* the putative PKS-bound diketide, triketide and tetraketide intermediates **15**, **16** and **17** to give prestrobilurin **18** which would then undergo rearrangement of the epoxide **19***via***20** to the formyl-carboxylic acid **21**/**22**. Finally, two *O*-methylations using SAM as cofactor complete the biosynthesis of strobilurin A **2**. A number of these points are discussed below.

**Scheme 2:**
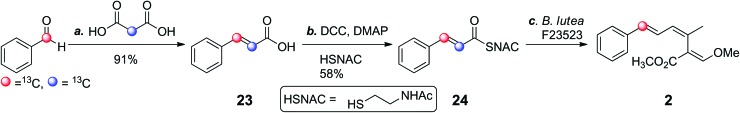
Reagents and conditions: (a) [2-^13^C]-malonic acid (1.02 equiv.), Na_2_SO_4_ (0.15 equiv.), pyridine, piperidine, reflux, 4 h, 91%; (b) DCC (1.13 equiv.), DMAP (0.04 equiv.), HSNAC (1.43 equiv.), DCM, 0 °C, 2 h, 23 °C, 16 h, 58%.

Cinnamate **13** is in the unusual position of potentially acting as the source of the benzoate starter, but also as a polyketide chain elongation intermediate (**15**, Scheme 1b). Feeding of [2,3-^13^C_2_]-phenylanine resulted in only a single ^13^C label (from C-3) being incorporated, indicating that cinnamate cannot be incorporated intact into the strobilurins. However, precursor-directed biosynthesis studies (discussed in detail below) with ^19^F-labelled benzoates and cinnamates gave consistently higher incorporation of fluorine label into fluorostrobilurin analogues from cinnamate (78%) than from benzoate (30%) for the 4-fluoro SNAC thiolester analogues. This potential contradiction was re-examined by synthesis (Scheme 2) and feeding of [2,3-^13^C_2_]-cinnamate both as the free acid **23** and its SNAC thiol ester **24**. SNAC thiol esters have been commonly used in studies of polyketide biosynthesis to mimic the thiol ester linkage of co-enzyme A and acyl carrier protein (ACP) and are usually incorporated into biosynthetic pathways more readily than the parent carboxylic acids.^26^,^27^ In this case, however, free cinnamic acid **23** was incorporated significantly more efficiently (85%) than the SNAC thiolester **24** (40%, Fig. S1†). In contrast, [carboxyl-^13^C]-benzoate is incorporated more efficiently as its SNAC thiol ester compared to the free acid.

**Scheme 3:**
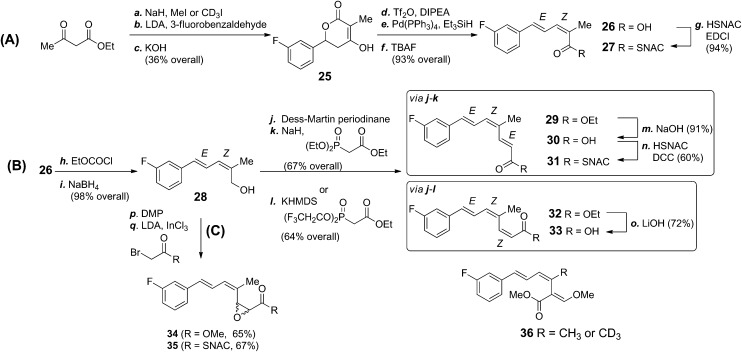
(a) NaH (60%, 1.08 equiv.), THF, 23 °C, 0.5 h, 50 °C, 20 h, 56%; (b) DIPA (2.5 equiv.), *n*-BuLi (2.5 equiv.), HMPA (1 equiv.), 3-fluorobenzaldehyde (1.1 equiv.), THF, –78 °C, 3 h; (c) 1 M KOH_(aq)_, 23 °C, 14 h, then 6 M HCl_(aq)_, 0 °C, 65% (over 2 steps); (d) DIPEA (1.5 equiv.), Tf_2_O (1.1 equiv.), CH_2_Cl_2_, –78 °C, 1 h, 97%; (e) Pd(PPh_3_)_4_ (0.01 equiv.), Et_3_SiH (2.0 equiv.), DMF, 60 °C, 2 h, 99%; (f) TBAF (1 M, 5 equiv.), THF, 23 °C, 2 h, 97%; (g) EDCI·HCl (1.6 equiv.), DMAP (1.2 equiv.), HSNAC (1.5 equiv.), DCM, 0 °C, 2 h, 23 °C, 14 h, 94%; (h) Et_3_N (2 equiv.), EtOCOCl (1.3 equiv.), THF, 0 °C, 0.5 h; (i) NaBH_4_ (2.5 equiv.), MeOH, –78 °C, 4 h, 60% (over 2 steps); (j) Dess–Martin periodinane (0.3 M, 1.3 equiv.), CH_2_Cl_2_, 23 °C, 1.5 h; (k) NaH (60%, 1.4 equiv.), (EtO)_2_P(O)CH_2_CO_2_Et (1.7 equiv.), THF, 0 °C, 10 min, 23 °C, 16 h, 67% (over 2 steps); (l) (CF_3_CH_2_O)_2_P(O)CH_2_CO_2_Et (2.1 equiv.), KHMDS (15 wt%, 2 equiv.), 18-crown-6 (2.4 equiv.), THF, –78 °C, 6.5 h, 64% (2*Z* : 2*E*::5 : 1, over 2 steps); (m) 1 M NaOH_(aq)_, THF, 23 °C, 91%; (n) EDCI·HCl (2 equiv.), DMAP (2.4 equiv.), HSNAC (8.9 equiv.), DCM, 23 °C, 16 h, 60%; (o) 1 M LiOH_(aq)_, MeOH, 23 °C, 16 h, 72%; (p) DIPA (2 equiv.), *n*-BuLi (2 equiv.), InCl_3_ (0.6 equiv.), THF, –78 °C, 2 h, 23 °C, 1 h, **34** (65%, over 2 steps), **35** (67%, over 2 steps); (q) NaH (60%, 1.4 equiv.), (EtO)_2_P(O)CH_2_CO_2_Et (1.7 equiv.), THF, 0 °C, 10 min, 23 °C, 16 h, 81% (over 2 steps).

Most fungal PKS belong to the Type I iterative class consisting of a single multi-domain protein encoded by a single gene.^28^ They have been classified according to the increasing degree of reductive modifications and *C*-methylations they carry out. One major class, the highly-reducing Type II (HR II) have a *trans*-acting stand-alone ER domain^29^ while a minority are Type III systems^29^ which do not catalyse β-processing reactions.^30^ The strobilurin PKS is likely to be a Type I PKS and catalyse three iterations of chain-extension and β-processing to produce the three enzyme bound intermediates **15–17** shown in Scheme 1b. In order to investigate the intermediacy of these compounds we decided to feed analogues of **16** and **17** to cultures of *B. lutea*. To aid metabolite analysis they were synthesised with a fluorinated phenyl ring as fluorine has been demonstrated to be a useful tracer for biosynthetic studies.^31^ Accordingly, 3-fluorobenzaldehyde was converted to the 2*Z*,4*E* isomer of 2-methyl-5-phenylpenta-2,4-dienoic acid **26** and its SNAC thiol ester **27** (Scheme 3A). Key synthetic steps included methylation of ethyl acetoacetate followed by condensation with 3-fluorobenzaldehyde and cyclisation to give the 3-hydroxy-lactone **25**. Following reductive removal of the hydroxyl group, ring opening of the resulting unsaturated lactone using TBAF gave acid **26** which was esterified to the corresponding thiol ester **27** in 94% yield. The *Z*-geometry of the 2,3-double bond was confirmed by nOe studies.

Both precursors **26** and **27** were pulse fed to cultures of *B. lutea* after 24, 36 and 48 hours according to the protocol used for previous feeding studies. LCMS analysis of the culture extract, however, failed to detect any fluorinated strobilurin analogues but the peaks corresponding to **26** and **27** remained with no obvious degradation of triketide to benzoate occurring as had been observed to occur for the free acid and SNAC thiolester of the diketide (cinnamate) **15**.

Tetraketide **18** (Scheme 1) is proposed to be the first enzyme-free product of the PKS and its fluoro-analogue was also synthesised as both the free acid **30** and SNAC thiol ester **31** (Scheme 3B). Acid **26** was reduced to primary alcohol **28** and, following oxidation to an aldehyde was chain extended under Horner–Wadsworth–Emmons conditions to ester **29** with the 2*E* double bond. Hydrolysis of ester **29** gave acid **30** which was converted to thiol ester **31**. The isomeric acid **33** with the 2*Z*-double bond was prepared using a similar strategy but with a Still–Gennari reaction for the chain extension.

**Fig. 1:**
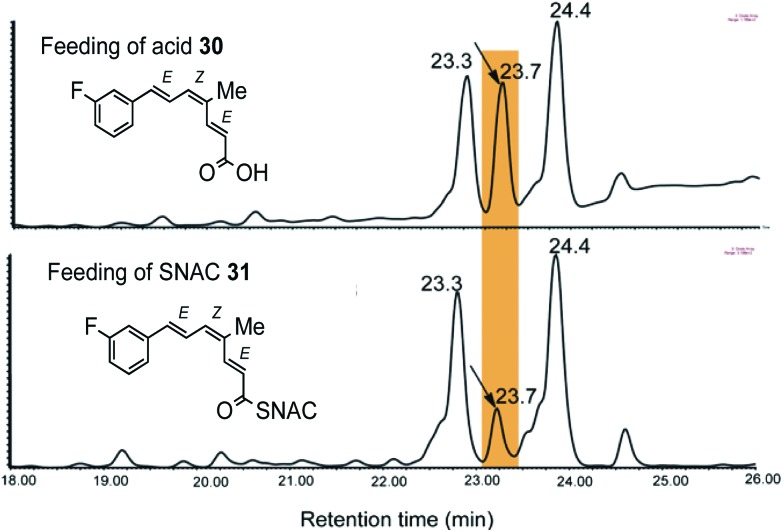
LCMS analysis shows that the tetraketide **30** fed to culture of *B. lutea* yields more of the enriched metabolite than the corresponding SNAC thiol ester **31**.

Acid **30** and thiol ester **31** were pulse fed to *B. lutea* in separate experiments. On extraction, and LCMS analysis (Fig. 1), a new metabolite was detected at 23.7 min, between the peaks for strobilurin A (23.3 min) and strobilurin B (24.4 min) with a molecular ion [M + Na]^+^ 299 Da, 18 units heavier than strobilurin A **2**. Interestingly, no fluorinated analogue of strobilurin B **3** was detected in either experiment, suggesting that fluorine in the phenyl ring inhibits hydroxylation. The yield of the new metabolite was *ca.* three times higher from the free acid **30** than from the SNAC ester **31** (Fig. 1). The metabolite 3-fluorostrobilurin **36** was purified and its structure confirmed by ^1^H and ^19^F NMR analysis. The proton resonances for the trienoic acid side chain are essentially identical to those of strobilurin A **2** as expected, confirming the 7*E*,9*Z*,11*E* configuration, but the aromatic ring shows the coupling pattern predicted for a 3-fluoro substituent, in particular vicinal H–F couplings of 10.1 and 8.8 Hz respectively to H-4 and H-2, and a 4 bond H–F coupling (5.8 Hz) to H-5. The ^19^F NMR spectrum shows a signal at –114 ppm as a double, double, doublet with the same coupling constants (Fig. S2b†).

As the trienoic acid **18** has not been detected in extracts of *B. lutea*, it suggests that on release of **17** from the PKS, this first enzyme-free intermediate is rapidly converted to strobilurins (Scheme 1b) as discussed in more detail below. At this stage we could not rule out that the observed incorporation of fluorine label was occurring *via* degradation of **30** or **31** to 3-fluorobenzoate and reincorporation, as observed for cinnamate. We thus synthesised an isotopomer of **30** (Scheme 3B) with a trideuteriomethyl at C-14 in addition to the original C-3 fluorine label. Incubation of the isotopomer with *B. lutea* and LCMS analysis of the extract showed a new peak at 24.3 min (strobilurin A 23.9 min) with the correct molecular ion ([M + H]^+^ 280 Da) for the trideuterio-labelled analogue of 3-fluorostrobilurin **36** to indicate intact incorporation of **30**. The structure was again confirmed by full NMR analysis. The ^19^F NMR spectrum was identical to that previously obtained for **36**, as was the ^1^H NMR spectrum except for the absence of the signal at *δ* 1.98 ppm for the three protons of the 14-methyl. This confirms that tetraketide **17** is indeed the final enzyme-bound product of the strobilurin PKS.‖
‖The 2*Z* isomer **33** was also synthesised and fed as a 5 : 1 mixture with the 2*E* isomer. A small incorporation into **36** was observed, but this is likely to have been due to the small amount of *E*-isomer present in the fed mixture.


Conversion of tetraketide **18** (thus named prestrobilurin A) to strobilurin A is proposed to proceed *via* epoxidation and rearrangement to give aldehyde **21**, enolisation to **22** and methylation forming the β-methoxyacrylate moiety (Scheme 1b). To further investigate this process we synthesised the epimeric epoxides **34** and **35** with a fluorine label (Scheme 3C). The allylic alcohol **29** was oxidized by Dess–Martin periodinane, and then the unstable aldehyde was treated with the methyl ester or SNAC thiolester of α-bromoacetate under Darzens reaction conditions. However, on feeding to *B. lutea* no incorporation into strobilurins, direct or indirect could be detected. In addition, fermentation of *B. lutea* in the presence of varying concentrations of the known cytochrome P450 monooxygenase inhibitor ancymidol^32^ had no effect on strobilurin production. Thus no direct evidence for an epoxide-mediated arrangement is available.

**Scheme 4:**
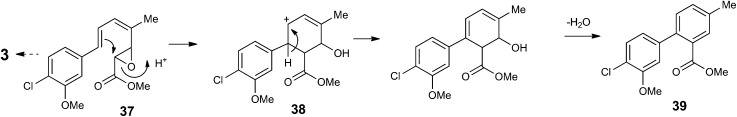
Conversion of epoxide **37** to prestrobilurin B **39**.

However, on investigating minor components of a second strain of *B. lutea* (strain F23523, which in our hands produced strobilurin B **3** as its major metabolite) we observed a minor peak eluting after strobilurin B **3** with a molecular ion [M + H]^+^ 291 Da with a ^37^Cl isotopic peak at 293 Da. We purified this metabolite by preparative HPLC, and isolated 0.8 mg of pure compound for NMR characterisation. Signals for a 3-methoxy-4-chloro-phenyl ring as in strobilurin B **3** were evident, but those for the triene side chain were absent, being replaced by signals consistent with the presence of a second 1,2,4-trisubstituted aromatic ring. COSY and HMBC correlations (Fig. S3†) were consistent with the biphenyl structure **39** (Scheme 4). We propose that this novel compound is produced by an alternative rearrangement of the key epoxide intermediate **37** (*cf.***19** in Scheme 1b) where the initial carbocation intermediate from epoxide ring opening undergoes electrophilic addition to the C-6/C-7 double bond, to give the cyclohexenyl cation **38**, which is then aromatised by proton loss and dehydration to give **39** which we have named pseudostrobilurin B. In support of this hypothesis is the fact that **37** must possess the predicted 4*Z*,6*E* geometry for the cyclisation to occur.

**Scheme 5:**
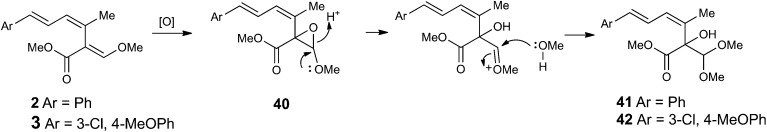
Formation of strobilurins Y **42** and Z **43***via* methanolysis of epoxide **40**.

Further detailed analysis of LCMS chromatograms of *B. lutea* extracts revealed the presence of two new minor compounds (each *ca.* 1 mg L^–1^) with UV characteristics typical of strobilurin A and B. These were isolated and their structures **41** and **42** determined from their mass spectra and full NMR analysis (Fig. S4†). They were named strobilurin Y **41** and strobilurin Z **42** respectively. Their formation can be rationalised by methanolysis of the epoxide **40** derived by a P_450_ or similar mono-oxygenase-mediated oxidation of strobilurins A and B (Scheme 5). In order to rule out formation of these compounds from methanol used as solvent in the original LCMS purification, we repeated the isolation using acetonitrile in place of methanol. Their continued production confirmed that they are the result of enzymatic transformations.

**Scheme 6:**
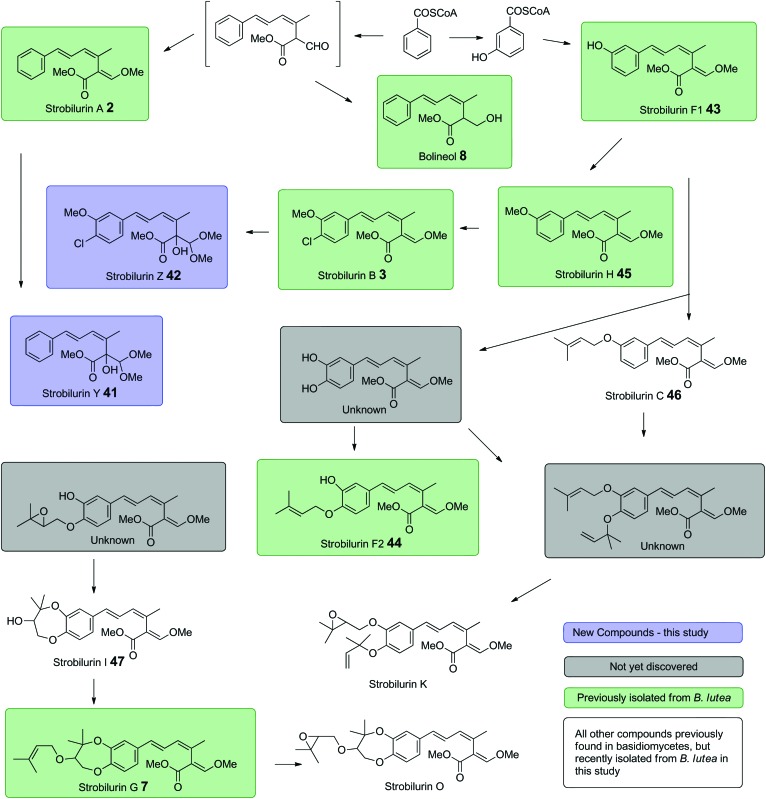
Proposed biosynthetic inter-relationships among strobilurin related metabolites in *B. lutea*.

In addition, a number of other strobilurins previously isolated from *B. lutea* were observed after optimisation of fermentation conditions (Fig. S5†). Besides the major metabolites, strobilurins A **2** and B **3**, these include strobilurins F1 **43**, F2 **44**, G **7**, H **45** and bolineol **8**. We also observed a number of very minor compounds which were identified by NMR and mass spectrometric characteristics. These are strobilurin C **46** and strobilurin I **47** previously isolated from *Xerula longipes*^33^ and an agaricus species^34^ respectively. Strobilurin C **46** appears to be the earliest prenylated strobilurin, presumably formed from strobilurin F1 **43**, whereas strobilurin I **47** is likely to be an intermediate to strobilurin G **7**. The likely overall biosynthetic interrelationships are summarised in Scheme 6.

Precursor directed biosynthesis, in which analogues of natural substrates are fed to either WT or mutant producing cultures has proved an effective method for producing analogues of microbial natural products, particularly for producing halogenated analogues.^35^ The presence of halogens often has a beneficial effect for enhancing biological activities. For example, the presence of a halogen is required for bioactivity in the case of salinosporamide, neomangicols and rebeccamycin,^36^,^37^ their non-halogenated analogues being inactive.

**Fig. 2:**
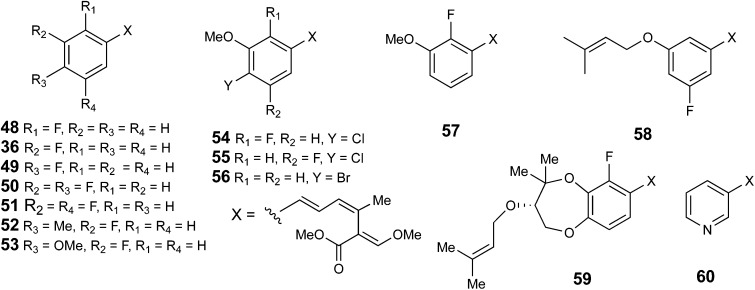
Strobilurin analogues produced by precursor-directed biosynthesis.

As indicated above, fluorinated precursors can be efficiently incorporated into strobilurins. With this in mind we made a more systematic study aimed at producing novel fluorinated, chlorinated and brominated analogues. The outcomes of these experiments were dependent on the producing strain used. Our initial studies used *B. lutea* strain F24510. A series of fluorinated cinnamates and benzoates were fed both as the free acid and SNAC thiol esters. The products were observed by mass spectrometric and ^1^H and ^19^F NMR analysis of the extracts after partial purification by thin layer chromatography and the results are summarised in Fig. 2. Mono- and di-fluorinated analogues were incorporated and the structures confirmed by NMR analysis, but individual analogues were not always isolated. Similar results were obtained with both *B. lut*ea F23523 and *S. tenacellus*, but variations were observed in that relative efficiencies of benzoate and cinnamate incorporations were sometimes reversed with benzoates being preferred by *S. tenacellus*.

However, feeding of 2-, 3- and 4-fluorobenzoate/cinnamate gave the corresponding fluorostrobilurins **48**, **36** and **49** in *S. tenacellus* in relative yields (compared to levels of strobilurin A) of 8–30% with 2-fluorostrobilurin **48** consistently the poorest yield. The 3,4 and 3,5-difluoro-analogues **50** and **51** were formed from the corresponding benzoates, and feeding 3-fluoro-4-methyl- and 3-fluoro-4-methoxybenzoates gave the corresponding fluorostrobilurins **52** and **53**. Feeding of nicotinic acid gave the novel 3-aza-strobilurin A analogue **60** and supplementation of the medium with KBr produced the bromo-analogue 4-dechloro-4-bromostrobilurin B **56**. Other attempts to introduce halogens, *e.g.* by supplementation with chloride, bromide or iodide, or by adding chloro- or bromobenzoates gave only trace amounts (by LCMS), at best, of alternatively halogenated compounds. Similar results were observed in feeding a range of aromatic analogues to squalestatin-producing cultures of *Phoma* sp.^38^ In this study a similar range of mono- and difluorinated benzoates were incorporated but no other halogens were accepted.

With *B. lutea* F23523, feeding of 2-fluorocinnamate gave an interesting result. Production of strobilurin A **2** was completely inhibited, the fermentation giving strobilurin B **3** along with about 50% 2-fluorostrobilurin B **48**, 10% 2-fluorostrobilurin G **59**, and about 1% 2-fluorostrobilurin H **57** (Fig. S6†). All structures were confirmed by NMR and mass spectrometric analysis. 3-Fluorocinnamate again produced 3-fluorostrobilurin A **36**, 4-fluorostrobilurin B **55** and 4-fluorostrobilurin C **58**. Finally 4-fluorocinnamate gave strobilurin A **2** along with 4-fluorostrobilurin A **49** only with no strobilurin B, presumably fluorine at C-4 inhibiting oxidative hydroxylation.

**Scheme 7:**
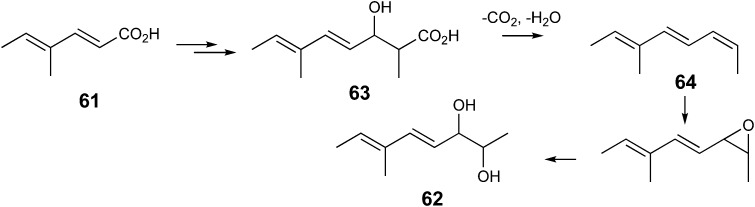
Proposed biosynthetic conversion of acid **61** to diol **62**.

The inhibition of strobilurin A production with 2-fluorocinnamate reflected the result of another experiment. The simple triketide analogue **61** was chemically synthesised (Scheme S1†) as a potential substrate analogue for the epoxidase believed to be responsible for the rearrangement to give the methoxyacrylate toxiphore. On feeding to *B. lutea* it was rapidly metabolised (Fig. S7†) to give a new compound shown to be diol **62**. The formation of **62** can be rationalised as summarised in Scheme 7 by a cycle of polyketide condensation, methylation and keto-reduction to give **63**. Concerted decarboxylation and dehydration to triene **64** followed by epoxidation and hydrolysis gives diol **62**, whose structure was established by 1D and 2D NMR analysis (Fig. S8†).

In this experiment, strobilurin B **3** production remains unaffected but no strobilurin A **2** is detected. Time course experiments (Fig. S9†) clearly indicate that the triketide analogue **61** is rapidly converted to the diol and that the diol itself is responsible for inhibiting strobilurin A production. In both these experiments, feeding acid **61** and 2-fluorocinnamate, strobilurin B production is unaffected. This strongly suggests that the pathways to strobilurin A **2** and strobilurin B **3** are different and that strobilurin A **2** is not converted directly to strobilurin B **3**, so that the pathways must diverge at an early stage. On feeding [2,3-^13^C_2_]-cinnamate to cultures inhibited as above, no restoration of strobilurin A **2** production is observed, but strobilurin B **3** is produced and labelled from the precursor. This suggest that in these experiments, incorporation post-benzoyl CoA into strobilurin A is being inhibited. This suggests that benzoyl CoA may first be hydroxylated to 3-hydroxybenzoyl CoA before elaboration into the strobilurin B pathway (Scheme 6).

## Conclusions

The strobilurins and analogues are amongst the most commercially important fungal metabolites known. Despite this their biosynthesis has been relatively little studied since the initial work of Nerud *et al.*^16^ In this study we have demonstrated that phenylalanine is converted to cinnamate, and then onwards to benzoate which forms the starter unit for formation of a linear tetraketide, prestrobilurin A **18**. The potential role of cinnamate as both the precursor to the starter unit and an assembly intermediate in its own right has been clarified. Prestobilurin A **18** undergoes oxidative rearrangement to form the core β-methoxyacrylate moiety, but no direct evidence for the process has yet been observed. However, indirect evidence for the intermediacy of an epoxide in the key rearrangement to form the methoxyacrylate toxiphore has been obtained here *via* the isolation pseudostrobilurin B **39** which is likely formed *via* the common epoxide intermediate **37**. Precursor-directed biosynthesis experiments demonstrated that a wide range of mainly fluorinated starter units can be tolerated by the PKS to provide several halogenated strobilurin analogues. Finally and unexpectedly, the production of strobilurin A **2** can be selectively inhibited with no effect on strobilurin B **3** biosynthesis. This suggests that they are produced by parallel pathways and that strobilurin A **2** is not an intermediate to strobilurin B **3**. Further work on the genetic and molecular basis for strobilurin biosynthesis is underway and will be reported elsewhere.

## Experimental

Full experimental details are supplies in the ESI.†

## Conflicts of interest

There are no conflicts of interest to declare.

## Supplementary Material

Supplementary information
