# Supplementary material for: Investigations into the biosynthesis of the antifungal strobilurins
Source: Org Biomol Chem. 2018 Jul 20;16(30):5524–32. doi: 10.1039/c8ob00608c (PMC6085771; doi:10.1039/c8ob00608c)
Supplement: Supplementary file 1 [file OB-016-C8OB00608C-s001.pdf]

Electronic Supplementary Material (ESI) for Organic & Biomolecular Chemistry.
This journal is © The Royal Society of Chemistry 2018

Investigations into the biosynthesis of the antifungal strobilurins: biosynthetic inter-relationships and novel halogenated strobilurins from precursor-directed biosynthesis

Zafar Iqbal,[#a,d] Li-Chen Han, [#a] Anna M. Soares-Sello,[a] Risa Nofiani,[a,e] Gerald Thormann,[b] Axel Zeeck,[b] Russell J. Cox,[c] Christine L. Willis,*[a] and Thomas J. Simpson*[a]

a School of Chemistry, University of Bristol, Cantocks Close, Bristol, BS8 1TS, UK

b Institut für Organische und Biomolekulare Chemie ,Tammannstraße 2, 37077 Göttingen.

c Institute for Organic Chemistry, Leibniz Universität, Hannover

d Current address, Department of Agriculture, Faculty of Nutrition Sciences, The University of Agriculture Peshawar, Pakistan

e Current address, Department of Chemistry, Faculty of Mathematics and Natural Sciences, University of Tanjungpura, Jl. Prof. Dr. H. Hadari Nawawi, Pontianak, Indonesia 78124

* corresponding authors: tom.simpson@bristol.ac.uk, chris.willis@bristol.ac.uk

Electronic Supplementary Information

1. Fermentations, Natural Product Purification and Analysis **1**
2. Synthesis of intermediates **8**
3. Figures and schemes
4. NMR data of Synthetic and Isolated Compounds **15**
4. References **55**

1. Fermentations, Natural Product Purification and Analysis, Feeding studies

Selection of Fungal Strains

Bolinea lutea strains F23523 and F24150 were obtained as a gift from Novartis, Switzerland in 2007. Two strains of *Strobilurus tenacellus,* one gifted by Prof. Zeeck in Germany and other obtained from CBS-KNAW, Fungal Diversity Centre, Netherland were selected for preliminary screening.

Strobilurus tenacellus: Maintenance and Fermentation

S. tenacellus culture was received on malt extract agar (MEA) medium in a glass ampule. For maintenance it was transferred to an agar plate on MEA medium (50 $g{\cdot}L^{-1}$ water). The strain grew well on the agar plates and was inoculated into liquid shake flask culture in M2 medium (10 g malt extract, 4 g yeast extract, 4 g glucose L^{-1}) as seed culture and incubated for 5 days at 22 °C and 150 rpm. Production cultures in MEA medium (150 mL) in 500 mL conical flask were inoculated with the seed culture (10 mL) and grown at 22 °C and 150 rpm. After 7-10 days of shake flask incubation the liquid culture was collected and filtered under vacuum.

Bolinea lutea: Agar Plate Preparation and Storage

Both strains of *B. lutea* were preserved as frozen culture. They were grown on agar plates on YGM medium containing yeast extract (0.4%), glucose (0.4%) and malt extract (1%) in water supplemented with 2% each of oatmeal and agar. The autoclaved agar solution was then melted in a microwave and poured into sterile petri dishes. A sterile loop was used to inoculate the agar plates from the frozen glycerol stock culture. The plates were left to grow at 25 °C for 5 days or when a substantial amount of biomass is spread throughout the plate. The mature agar slants could either be used to inoculate seed culture or stored for future use (viable up to 6 months in refrigerator at 4 °C). For long storage the mycelia from mature plates were also

transferred to a sterile solution of 20% *w/v* glycerol in water and stored in 1.5 mL pre-sterilized vial at -80 °C.

***Bolinea lutea:* Seed Culture Preparation**

An aqueous medium consisting of 3% glucose, 1% maltose, 0.4% yeast extract and 2% oatmeal was prepared in 100 mL in 250 mL conical flask. The flasks were plugged with a foam bung and capped with aluminium foil prior to autoclave sterilization. It was then inoculated with the mycelia (1/4th portion of one mature agar plate) and then incubated for 3-5 days on a rotary shaker at 25 °C and 150 rpm until a well grown mass of fungi was achieved.

***B. lutea:* Production Culture Preparation**

The production medium consists of 3% glucose, 1% malt extract, 0.4% yeast extract and 2% oatmeal in water. The pH was adjusted to 7.5 with dil. NaOH solution before sterilization. Transferred 150 mL to a 500 mL flask and inoculated with 10 mL of seed culture under aseptic conditions and cultivated for 7-10 days at 25 °C, 150 rpm.

Optimisation of Culture Conditions

To overcome initial production problem optimization of fermentation conditions was carried out for enhancing strobilurin production. Five different media were tested: glucose nutrient broth (GNB); malt extract broth (MEB); yeast extract, glucose, malt extract, (YGM); complete medium broth (CMB); and potato dextrose broth (PDB). GNB medium was selected for future work for its better yield. Variation in pH of production medium before inoculation was also checked. pH 4-9 by the addition of dil. NaOH or HCl was adjusted. Fungi were grown in baffled flasks and the metabolite profile was compared to see the effect of aeration. Different sets of temperature (20-37 °C) and shaking (50-250 rpm) were also evaluated.

Extraction of Metabolites and LCMS Analysis

Strobilurus tenacellus

A 1 L *S. tenacellus* culture after 10 days of fermentation was filtered using vacuum and washed several times with distilled water. The wet cells (31 g) were extracted with MeOH/Acetone (2:1) and then with MeOH. The combined extracts were evaporated to remove most of the acetone and the metabolites were extracted with 100 mL $CHCl_3$ from the residue. The organic extract was dried over anhydrous $MgSO_4$, filtered and evaporated *in vacuo*, yielding 500 mg of dark brown oil. The crude extract solution (10 mg·mL^{-1} in MeOH) was first centrifuged to remove solids and then subjected to LCMS analysis.

Bolinea lutea

The whole cell 1 L culture (7-10 days) was homogenized and a 0.5 L solution of MeOH/EtOAc (3:2) was added to the extract overnight. Filtration under vacuum, and the filtrate was concentrated *in vacuo*. The concentrate was again extracted with $CHCl_3$ (3 × 150 mL). The organic solvents were dried over anhydrous $MgSO_4$ and evaporated *in vacuo*. The extract was dissolved in acetonitrile and extracted with petroleum ether (2 × 100 mL) to remove fatty acids. The defatted acetonitrile extract was dried to give 600 mg of yellow oil.

LCMS Analysis

A solution of the crude extract (10 mg·mL^{-1}) was prepared in HPLC grade MeOH and centrifuged for removal of solids. 50 µL of this solution was injected to a Phenomenex Luna 5µ C_{18} (II) (250 × 4.6 mm) reversed phase column. A solvent system of MeOH (B) and water (A) with 0.05% formic acid each was used. The sample was run for one hour program (0-5 min: 75% A, 25% B; 5-51 min: 5% A, 9 5% B; 51-53 min: 5% A, 95% B; 53-55 min: 75% A, 25% B; 55-60 min: 75% A, 25% B), at 1 mL·min^{-1} flow rate.

Preparative Thin Layer Chromatography and isolation for isolation of Strobilurin A and B

Crude *B. lutea* extract (50-70 mg) was loaded on a silica gel coated TLC glass plate (20 × 20 cm). The plate was developed with 10% EtOAc in petroleum ether (4 consecutive elutions) to afford 5 bands. A yellow pure band at Rf 0.84 corresponded to strobilurin A **2**, while strobilurin B **3** appeared at Rf 0.56 as a bright yellow band. Strobilurin H **45** is a minor metabolite which appeared in mixture with strobilurin B **3** which could not be separated by preparative TLC. The target bands were carefully scraped off and extracted with EtOAc for further characterization.

Strobilurin A **2** (25-35 mg·L^{-1}) was isolated as a yellow oil: λ_{max} (MeOH) = 228, 293 nm; ν_{max} (KBr) 2952, 1709, 1628, 1437, 1244, 1121, 1068, 693 cm^{-1}; δ_H (500 MHz, $CDCl_3$), 1.98 (3H, br s, CH_3-14), 3.74 (3H, s, OCH_3-15), 3.85 (3H, s, OCH_3-16), 6.26 (1H, br d, *J* 10.8, H-9), 6.49 (1H, d, *J* 15.5, H-7), 6.62 (1H, dd, *J* 15.5, 10.8, H-8), 7.18 (1H, tt, *J* 7.3, 1.8, H-4), 7.28 (2H, m, H-3, H-5), 7.34 (2H, m, H-2, H-6), 7.43 (1H, s, H-12); δ_C (100 MHz, $CDCl_3$) 23.6 (14-CH_3), 51.5 (15-OCH_3), 61.8 (16-OCH_3), 110.7 (C-11) , 126.5 (C-2, C-6), 127.1 (C-4), 128.5 (C-8), 129.8 (C-3, C-5), 131.1 (C-7), 131.4 (C-9), 132.4 (C-10), 137.8 (C-1), 158.8 (12-CH), 167.7 (13-C=O); *m/z* (ESI) 281 (MNa^+, 35%), 259 (MH^+, 45%), 227 (MH^+- MeOH, 70%). 167 (M^+- CO_2Me, -MeOH, 100%).

Strobilurin B **3** (35-40 mg·L^{-1}) was obtained as a yellow oil: λ_{max} (MeOH) = 228, 302 nm; ν_{max} (KBr) 3467, 2952, 1725, 1588, 1409, 1255, 1029, 1074, 755, 667 cm^{-1}; δ_H (500 MHz, $CDCl_3$), 1.98 (3H, br s, CH_3-14), 3.74 (3H, s, OCH_3-15), 3.85 (3H, s, OCH_3-16), 3.91 (3H, s, OCH_3-17), 6.26 (1H, br d, *J* 10.6, H-9), 6.43 (1H, d, *J* 15.6, H-7), 6.57 (1H, dd, *J* 15.6, 10.6, H-8), 6.84 (1H, d, *J* 1.8, H-2), 6.92 (1H, dd, *J* 8.3, 1.8, H-6), 7.25(1H, d, *J* 8.3, H-5), 7.43 (1H, s, H-12); δ_C (100 MHz, $CDCl_3$) 23.7 (14-CH_3), 51.6 (15-OCH_3), 56.1 (16-OCH_3), 61.9 (17-OCH_3), 110.1 (C-2), 110.7 (C-11), 119.1 (C-4), 121.1 (C-6), 127.2 (C-5), 130.4 (C-7), 130.5 (C-8), 130.6 (C-9), 132.1 (C-10), 137.9 (C-1), 155.0 (C-3), 158.9 (C-12), 167.8 (13-C=O); *m/z* (ESI) 345/347 (MNa^+, MNa^+ + 2 (^{37}Cl), 33%/12%), 323/325 (MH^+, MH^+ + 2 (^{37}Cl), 22%/ 8%), 291/293 (MH^+- MeOH, MH^+- MeOH + 2 (^{37}Cl), 100%/ 33%), 231/233 (M^+ - CO_2Me, MeOH, - CO_2Me, MeOH + 2 (^{37}Cl), 100%/ 35%).

Characterisation of known (*B. lutea*) Minor Strobilurin Metabolites

Crude extract (500-600 mg) obtained from 1L culture of *B. lutea* was partially purified by preparative TLC and then subjected to HPLC purification (Dionex system) and a Phenomenex Luna 5µ C_{18} (II) (250 × 4.6 mm) reversed phase column. A 50 min. program with gradient of HPLC grade MeOH and Water (+ 0.05% formic acid in water only) was used: 0-5 min, 60% A, 40% B; 5-38 min, 5% A, 95% B; 38-43 min, 5% A, 95% B; 43-45 min, 60% A, 40% B; 45-50 min, 60% A, 40% B at 1 mL·min^{-1} flow rate detected at (200-800) nm range. Fractions (0.5 mL) were collected in glass tubes by an automatic fraction collector. Metabolites collected were: strobilurin F1 **43** (Rt 17.6 min.); strobilurin F2 **44** (22.3 min.); strobilurin G **7** **(**26.6 min.); strobilurin H **45** (21.4 min.); and bolineol **8** (21.6 min.).

Strobilurin F1 **43** colourless oil (1-2 mg·L^{-1}): λ_{max} (MeOH) = 221, 299 nm; δ_H (500 MHz, $CDCl_3$), 1.97 (3H, br s, CH_3-14), 3.74 (3H, s, OCH_3-15), 3.85 (3H, s, OCH_3-16), 6.25 (1H, br d, *J* 10.6, H-9), 6.43 (1H, d, *J* 15.4, H-7), 6.60 (1H, dd, *J* 15.4, 10.6, H-8), 6.66 (1H, dd, *J* 7.8, 1.8, H-6), 6.83(1H, br s, H-2), 6.91 (1H, d, *J* 7.8, H-4), 7.15 (1H, t, *J* 7.8 H-5), 7.43 (1H, s, H-12); *m/z* (ESI) 297 (MNa^+, 100%), 275 (MH^+, 22%), 243 (MH^+- MeOH, 20%), 215 (M- CO_2Me, 14%), 183 (M-CO_2Me, -MeOH, 85%).

Strobilurin F2 **44** colourless oil (0.5-1 mg·L^{-1}): λ_{max} (MeOH) = 226, 301 nm; δ_H (500 MHz, $CDCl_3$), 1.74 (3H, s, CH_3-20), 1.80 (3H, s, CH_3-21), 1.97 (3H, br s, CH_3-14), 3.74 (3H, s, OCH_3-15), 3.85 (3H, s, OCH_3-16), 4.51 (2H, d, *J* 7.0, CH_2-17), 5.49 (1H, m, H-18), 6.25 (1H, br d, *J* 10.6, H-9), 6.43 (1H, d, *J* 15.5, H-7), 6.48 (1H, dd, *J* 15.5, 10.6, H-8), 6.76 (2H, m, H-5, H-6), 6.98 (1H, br s, H-2), 7.42 (1H, s, H-12); *m/z* (ESI) 381 (MNa^+, 50%), 359 (MH^+, 18%), 327 (MH^+ - MeOH, 30%).

Strobilurin H **45** yellowish oil (1-1.5 mg·L^{-1}): λ_{max} (MeOH) = 226, 295 nm; δ_H (500 MHz, $CDCl_3$), 1.98 (3H, br s, CH_3-14), 3.74 (3H, s, OCH_3-15), 3.82 (3H, s, OCH_3-17), 3.85 (3H, s, OCH_3-16), 6.27 (1H, br d, *J* 10.7, H-9), 6.47 (1H, d, *J* 15.5, H-7), 6.61 (1H, dd, *J* 15.5, 10.7, H-8), 6.77 (1H, ddd, *J* 8.0, 2, 1.4 H-4), 6.88 (1H, br s, H-2), 6.96 (1H, br d, *J* 7.8, H-6), 7.21 (1H, t, *J* 7.8, H-5), 7.43 (1H, s, H-12); *m/z* (ESI) 311 (MNa^+, 30%), 289 (MH^+,8%), 257 (MH^+ - MeOH, 70%).

Strobilurin G **7** yellow oil (1-3 mg·L^{-1}): λ_{max} (MeOH) = 221, 300 nm; δ_H (500 MHz, $CDCl_3$), 1.22 (3H, s, CH_3-20), 1.48 (3H, s, CH_3-21), 1.70 (3H, br s, CH_3-26), 1.77 (3H, br s, CH_3-25), 1.97 (3H, br s, CH_3-14), 3.51 (1H, dd, *J* 8.0, 3.3, H-18), 3.74 (3H, s, OCH_3-15), 3.85 (3H, s, OCH_3-16), 3.97 (1H, dd, *J* 12.5, 8.0, H-17), 4.07 (1H, br dd, *J* 11.7, 7.3, H-22), 4.17 (1H, br dd, *J* 11.7, 6.8, *H-22*), 4.25 (1H, dd, *J* , 12.5, 3.3, *H-17*), 5.35 (1H, m, H-23), 6.23 (1H, br d, *J* 10.6, H-9), 6.37 (1H, d, *J* 15.5, H-7), 6.48 (1H, dd, *J* 15.5, 10.6, H-8),), 6.84 (1H, d, *J* 8.5, H-5), 6.93 (1H, dd, *J* 8.5, 1.9 H-6), 6.95 (1H, br s, H-2), 7.43 (1H, s, H-12); *m/z* (ESI) 465 (MNa^+, 100%), 443 (MH^+, 10%), 411 (MH^+ - MeOH, 70%).

Bolineol **8** pale yellow oil (12-15 mg·L^{-1}): λ_{max} (MeOH) = 222, 291nm; δ_H (500 MHz, $CDCl_3$) 1.81 (3H, br s, CH_3-14), 3.68 (1H, dd, *J* 10.6, 5.2, H-11), 3.74 (3H, s, OCH_3-15), 3.98 (1H, dd, *J* 8.6, 5.2, H-12a), 4.10 (1H, dd, *J* 10.6, 8.6, H-12b), 6.22 (1H, br d, *J* 11.0, H-9), 6.54 (1H, d, *J* 15.3, H-7), 7.00 (1H, dd, *J* 15.3, 11.0, H-8), 7.23 (1H, t, *J* 7.3, H-4), 7.32 (2H, dd, *J* 7.6, 7.3, H-3, H-5), 7.40 (2H, d, *J* 7.6, H-2, H-6); *m/z* (ESI) 269 (MNa^+, 70%), 247 (MH^+, 30%), 217 (MH^+ - CH_2OH, 45%), 188 (MH^+ - CO_2Me, 20%), 185 (MH^+ - CH_2OH, - MeOH 33%).

Other Known Strobilurins identified in *B. lutea*

Following the above procedures two strobilurins previously known, but not previously identified in *B. lutea:* strobilurin C **46** (Rt 31.8 min.) and strobilurin I **47** (19.1 min.) were also isolated.

Strobilurin C **46** colourless oil (0.5-1 mg.L^{-1}): λ_{max} (MeOH) = 222, 298 nm; δ_H (500 MHz, $CDCl_3$) 1.77 (3H, s, CH_3-20), 1.82 (3H, s, CH_3-21), 1.97 (3H, br s, CH_3-14), 3.74 (3H, s, OCH_3-15), 3.85 (3H, s, OCH_3-16), 4.51 (2H, d, *J* , 7.0, H-17), 5.53 (1H, m, H-18), 6.27 (1H, br d, *J* 10.5, H-9), 6.47 (1H, d, *J* 15.5, H-7), 6.63 (1H, dd, *J* 15.5, 10.5, H-8),), 6.78-6.93 (3H, Ar m, H-2, H-4, H-6), 7.21(1H, dd, *J*, 7.8, H-5), 7.43 (1H, s, H-12); *m/z* (ESI) 365 (MNa^+, 60%), 343 (MH^+, 10%), 311 (MH^+ - MeOH, 24%).

Strobilurin I **47 y**ellow oil (0.1-0.5 mg·L^{-1}): λ_{max} (MeOH) = 219, 299 nm, δ_H (500 MHz, $CDCl_3$), 1.22 (3H, s, CH_3-20), 1.48 (3H, s, CH_3-21), 1.97 (3H, br s, CH_3-14), 3.71 (1H, dd, *J* 7.9, 3.3, H-18), 3.74 (3H, s, OCH_3-15), 3.85 (3H, s, OCH_3-16), 3.91 (1H, dd, *J* 12.5, 7.9, H-17), 4.25 (1H, dd, *J* 12.5, 3.3, *H-17*), 6.23 (1H, br d, *J* 10.6, H-9), 6.37 (1H, d, *J* 15.5, H-7), 6.48 (1H, dd, *J* 15.5, 10.6, H-8),), 6.84 (1H, d, *J* 8.5, H-5), 6.93 (1H, br d, *J* 8.5, H-6), 6.95 (1H, br s, H-2), 7.43 (1H, s, H-12); *m/z* (ESI) 397 (MNa^+, 100%), 375 (MH^+, 10%), 343 (MH^+ - MeOH, 50%), 325 (MH^+ - MeOH, - H_2O, 20%), 283 (MH^+ - CO_2Me, - MeOH, 25%).

Characterisation of Novel Strobilurin Analogues.

Following the above procedures the two novel strobilurin analogues strobilurin Y **41** (Rt 20.8 min.) and strobilurin Z **42** (23.5 min.) were isolated.

Strobilurin Y **41** yellow oil (0.7-1 mg·L^{-1}): λ_{max} (MeOH) = 210, 293 nm; δ_H (500 MHz, $CDCl_3$), 1.99 (3H, d, *J* 1.2 CH_3-14), 3.50 (3H, s, OCH_3-16), 3.54 (3H, s, OCH_3-17), 3.81 (3H, s, OCH_3-15), 4.92 (1H, s, H-12), 6.63 (1H, d, *J* 15.5, H-7), 6.68 (1H, dq, *J* 10.9, 1.2 H-9), 7.02 (1H, dd, *J* 15.5, 10.9, H-8), 7.23 (1H, tt, *J* 7.1, 1.5, H-4), 7.31 (2H, m, H-3, H-5), 7.42 (2H, m, H-2, H-6); δ_C (125 MHz, $CDCl_3$) 13.3 (CH_3-14), 52.9 (-OCH_3-15), 58.3 (OCH_3-16), 58.5 (OCH_3-17), 82.5 (C-11), 104.7 (C-12), 127.0 (C-9), 127.5 (C-2, C-6), 127.9 (C-4), 128.8 (C-3, C-5), 132.1 (C-8), 132.4 (C-10), 134.1 (C-7), 137.5 (C-1), 172.8 (13-C=O); HRESI-MS *m/z* 329.1354 [M]Na^+ (calcd. for $C_{17}H_{22}O_5Na^+$, 329.1359); *m/z* (ESI) 329 (MNa^+, 100%), 307 (MH^+, 10%), 275 (MH^+ - MeOH, 40%).

Strobilurin Z **42** yellow oil (0.8-1 mg·L^{-1}): λ_{max} (MeOH) = 221, 300 nm; δ_H (500 MHz, $CDCl_3$), 1.99 (3H, d, *J* 1.2, CH_3-14), 3.50 (3H, s, OCH_3-16), 3.52 (3H, s, OCH_3-17), 3.81 (3H, s, OCH_3-15), 3.93 (3H, s, OCH_3-18), 4.92 (1H, s, H-12), 6.56 (1H, d, *J* 15.5, H-7), 6.66 (1H, dq, *J* 10.9, 1.2, H-9), 6.92 (1H, d, *J* 1.8, H-2), 6.96 (1H, dd, *J* 8.2, 1.8, H-6), 6.98 (1H, dd, *J* 15.5, 10.9, H-8), 7.28(1H, d, *J* 8.2, H-5); δ_C (125 MHz, $CDCl_3$) 13.6 (14-CH_3), 52.9 (OCH_3-15), 56.1 (OCH3-16), 56.2 (OCH_3-17), 57.53 (OCH_3-18), 82.2 (C-11), 104.5 (C-12), 109.8 (C-2), 119.4 (C-6), 121.6 (C-4), 125.1 (C-8), 127.0 (C-5), 130.2 (C-9), 133.0 (C-7), 134.3 (C-10), 137.5 (C-1), 155.0 (C-3), 172.1 (13-C=O); HRESI-MS *m/z* [M]Na^+ 393.1063 (calcd. for $C_{18}H_{23}ClO_6Na^+$, 393.1075); *m/z* (ESI) 393/395 (MNa^+, MNa^+ + 2

(^{37}Cl), 100%/35%), 371/373 (MH^+, MH^+ + 2 (^{37}Cl), 14%/ 5%), 339/341 (MH^+- MeOH, - MeOH + 2 (^{37}Cl), 45%/ 15%).

Pseudostrobilurin B 39

A novel biphenyl compound, pseudostrobilurin B **39**, an analogue of strobilurin B **3,** Rt at 48.9 min was collected using preparative HPLC a 60 min program with gradient of MeOH and Water (+ 0.05% formic acid each): 0-5 min, 75% A, 25% B; 5-51 min, 5% A, 95% B; 51-53 min, 5% A, 95% B; 53-55 min, 75% A, 25% B; 55-60 min, 75% A, 25% B, at 4 mL·min^{-1} flow rate at (200-400 nm) range. Fractions were collected in tubes in an automatic fraction collector.

Pseudostrobilurin B **39** yellow oil (0.1-0.2 mg·L^{-1}): λ_{max} (MeOH) = 210, 280 nm; δ_H (500 MHz, $CDCl_3$), 2.59 (3H, br s, CH_3-14), 3.89 (3H, s, OCH_3-15), 4.01 (3H, s, OCH_3-16), 7.24 (1H, dd, *J* 8.2, 2.0, H-6), 7.38 (1H, d, *J* 2.0, H-2), 7.42 (1H, d, *J* 8.0, H-8), 7.49 (1H, d, *J* 8.2, H-5), 7.78 (1H, dd, *J* 8.0, 2.1, H-9), 8.14 (1H, d, *J* 2.1, H-11); δ_C (100 MHz, $CDCl_3$) 21.3 (14-CH_3), 52.3 (15-OCH_3), 56.7 (16-OCH_3), 111.9 (C-2), 120.6 (C-6), 121.7 (C-4), 129.5 (C-11), 131.2 (C-5), 131.3 (C-9), 133.2 (C-8), 137.9 (C-7), 139.5 (C-10), 140.2 (C-1), 141.6 (C-12), 156.1 (C-3), 167.5 (13-C=O) HRCI-MS $[M]H^+$ *m/z* 291.0779 (calcd. for $C_{16}H_{16}ClO_3$, 291.0788); *m/z* (ESI) 291/293 (MH^+, MH^+ + 2 (^{37}Cl), 60%/ 22%), 259/261 (MH^+ - MeOH, - MeOH + 2 (^{37}Cl), 40%/15%).

Time Course Production Studies.

30 conical flasks (500 ml) each containing the production media (150 ml) were inoculated with mycelia from the seed culture (10 ml). The flasks were shaken at room temperature and 150 rpm. Each day one flask was collected and extracted following the standard protocol. The crude extract obtained from the *B. lutea* whole culture from each flask was weighed and dissolved in 3 mL MeOH. The solution was centrifuged for removal of solids. 50 µL of this solution was injected to the LCMS, a Phenomenex Luna 5µ C_{18} (II) (250 × 4.6 mm) reversed phase column. A solvent system of CH_3CN (B) and water (A) with 0.045% trifluoroacetic acid was used. The sample was run for one h program (0-5 min: 75% A, 25% B; 5-51 min: 5% A, 9 5% B; 51-53 min: 5% A, 95% B; 53-55 min: 75% A, 25% B; 55-60 min: 75% A, 25% B), at flow rate of 1 mL·min^{-1}.

A series of known concentrations of strobilurin A **2**, B **3**, G **16** and H **45** (0.03-1 mg·mL^{-1} in MeOH) were detected by diode array detector (200-400 nm). Calibration curves were plotted by area under HPLC peaks *vs*. a series of standard of known strobilurin concentrations. Crude extraction method was used to identify the compounds *via* LCMS analysis (retention times, UV characteristics and ESI-MS) and then peak integrations were performed to compare peak area with the corresponding standard curves for quantification.

General Procedure for Precursor Feeding

Conical flasks (500 mL), each containing 150 mL of the specified production medium were inoculated with the mycelia from the seed cultures (5-10 mL) as described earlier. The flasks were incubated at 25 °C and 150 rpm in a shaker. The selected proposed precursors were supplied as a pulse feed on days 2, 3 and 4 of cultivation as MeOH or DMSO solution. Controls in parallel were run with each experiment for systematic comparison. After fermentation for an appropriate duration the flasks were collected for extraction. Metabolites of interest were isolated following standard protocol.

Feeding and Incorporation of [2, 3-$^{13}C_2$]-Cinnamic Acid (23) and thiolester (24).

Following the above procedure [2, 3-$^{13}C_2$]-cinnamic acid **23** and its SNAC thiolester **24** were fed separately to the cultures of *B. lutea* as MeOH solution (0.05 mM). After 8-10 days of cultivation LCMS screening of the crude extract found all observed strobilurins enriched with precursors identified by their difference in *m/z* value as compared to control. The two major enriched metabolites eluting at Rt 41.7 min and 43.5 min were purified by preparative HPLC. Their structures were confirmed as strobilurin A and strobilurin B by NMR spectroscopy. The 13C NMR signals at 131.3and 130.3 ppm respectively showed incorporations of 70%.

Feeding of (2*Z,* 4*E*) 3-Fluorophenyl -2-methylpentadienoic Acid 26 SNAC Thiolester 27.

Following the standard feeding procedure **26** and it SNAC **27 (**15-20 mg each**)** were fed to the *B. lutea* culture (150 mL) and was extracted accordingly after 7-10 days of cultivation. LCMS analysis confirmed that 3-fluorostrobilurin A **36** was not produced in either case.

Feeding of (*2E, 4Z, 6E*)-3-fluorophenyl-4-methylheptatrienoic acid (30) and thiolester (31)

(*E,Z,E*) 3-fluorophenyl-4-methylhepta-trienoic acid **30** and its SNAC thiolester **31**were fed (150 mg·L^{-1}) to the whole cell culture of *B. lutea* following the above feeding procedures. After 8-10 days of fermentation the culture was extracted accordingly. The crude extract was centrifuged and then subjected to LCMS analysis. Initial LCMS analysis suggested that a new peak eluted at 23.1 min with difference of 18 in *m/z* value from natural analogue strobilurin A **2** is 3-fluorostrobilurin A **36** The peak was targeted for purification using the method above. The structure was confirmed as 3-fluorostrobilurin A by 1D, 2D NMR and HRMS investigations. (15-18 mg·L^{-1} culture, inoculated with 150 mg of **30.**

Production of [14-C^2H_3]-3-Fluorstrobilurin A

Following the above procedure(*E,Z, E*) [4-C^2H_3**]**-3-fluorophenyl-4-methylhepta-trienoic acid **30,** was fed to *B. lutea* (500 mL culture) at a oncentration of 100 mg·L^{-1}. The cultures were allowed to grow for 8-10 day at 25 °C and 150 rpm. The culture was collected and extracted using the standard procedure. The crude extract was dissolved in HPLC MeOH (10 mg·mL^{-1}) and centrifuged to remove solids before injection. 50 µL of this solution was injected for LCMS analysis. Initial analysis showed a new peak eluted at 24.2 min. The new peak was isolated as mixture with strobilurin A **2** (1:3). Repurification afforded a mixture of enriched-compound and strobilurin A again in 3:2 ratio (0.9 mg). The mixture was subjected to spectroscopic analysis and structure of the new compound was identified as [14-$^2H_3$**]**-3-fluorostrobilurin A: λ_{max} (MeOH) = 219, 300 nm; δ_H (500 MHz, $CDCl_3$), 3.75 (3H, s, OCH_3-15), 3.86 (3H, s, OCH_3-16), 6.26 (1H, br d, *J* 10.8, H-9), 6.46 (1H, d, *J* 15.8, H-7), 6.62 (1H, dd, *J* 15.8, 10.8, H-8), 6.88 (1H, dddd, *J* 9.1, 8.1, 2.5, 0.8, H-4), 7.05 (1H, ddd, *J* 10.5, 2.5, 1.8 H-2), 7.10 (1H, br d, *J* 7.6, H-6) 7.22 (1H, ddd, *J* 8.1, 7.6, 6.0 H-5), 7.44 (1H, s, H-12); HRCI-MS *m/z* 280.1437 $[M]H^+$ (calcd. for $C_{16}H_{15}D_3FO_3$, 280.1428); *m/z* (ESI) 302 (MNa^+, 50%), 280 (MH^+ 48%), 248 (MH^+- MeOH, 80%).

Precursor Directed Biosynthesis

2-Fluorocinnamic Acid

Following the procedure described above 2-fluorocinnamic acid, 150 mg was fed to 1 L culture of *B. lutea* in GNB medium. Fluorine-enriched metabolites were identified by LCMS analysis. The low titre of 2-fluorostrsobilurin H **45** and 2-fluorostrobilurin G **7** hindered their isolation for full NMR characterization. The enriched metabolites, 2-fluorostrobilurin A **48** and 2-fluorostrobilurin B **54** were purified and their structure was confirmed by spectroscopic analysis.

2-Fluorostrobilurin A **48** pale oil (1-5 mg·L^{-1} culture, inoculated with 2-fluorocinnamic acid, 250mg.). λ_{max} (MeOH) 227, 252, 292, 305 nm; (δ_H 600 MHz, CD_3OD): 1.90 (s, 3H, CH_3**)**, 3.70 (s, 3H, 15-OCH_3**)**, 3.84 (s, 3H, 16-OCH_3**)**, 6.18 (d, *J* 11.0, 1.5 Hz,1H, 9-H), 6.55 (d, *J* 16 Hz, 1H, 7-H),6.60 (dd, *J* 11.0, 16.0 Hz, 1H, 8-H), 7.01 (ddd, *J* 11.2, 8.0, 1.0 Hz, 1H, 3-H), 7.08 (td, *J* 8.0, 1.0 Hz, 1H, 5-H), 7.17 (dddd, *J* 8.0, 8.0, 5,5 2.0 Hz, 1H, 4-H), 7.41 (td, *J* = 8.0, 2.0 Hz, 1H, 6-H), 7.51 (s, 12-H) ppm. δ_C (125 MHz) 23.8 (C-14), 51.9 (C-16), 62.3 (C-15), 111.4 (C-11), 116.4 (C-1), 116.7 (C-3), 123.8 (C-7), 125.41 (C-5), 128.7 (C-6), 129.6 (C-4), 130.6 (C-8), 131.1 (C-9), 133.6 (C-10), 161.0 (C-12), 162,5 (C-2), 169.4 (C-13) ppm. Δ_F - 120.5 (ddd *J* = 11.2, 7.8, 5.2) ppm. HREI-MS m/z 276.1162 (calcd. for $C1_6H_{17}O_3F$, 276.1163).

2-Fluorostrobilurin B **54** pale yellow oil (10-15 mg·L^{-1} culture, inoculated with 150 mg of 2-fluorocinnamic acid): λ_{max} (MeOH) 231, 301 nm; δ_H (500 MHz, d_6-acetone), 1.92 (3H, br s, CH_3-14), 3.65 (3H, s, OCH_3-15), 3.89 (3H, s, OCH_3-16), 3.92 (3H, d, *J* 1.0 OCH_3-17) 6.24 (1H, br d, *J* 10.8, H-9), 6.55 (1H, d, *J* 16.0, H-7), 6.79 (1H, dd, *J* 16.0, 10.8, H-8), 7.16 (1H, dd, *J* 8.5, 1.5, H-6), 7.25 (1H, d, *J* 8.5, H-5), 7.47 (1H, s, H-12); δ_C (125 MHz,) 23.89 (14-CH_3), 51.5 (15-OCH_3), 61.7

(17-OCH_3), 62.2 (16-OCH_3), 110.79 (C-11), 121.8 (C-1), 121.8 (C-5), 122.1 (C-7), 126.2 (C-4) 126.4 (C-6) 130.4 (C-9), 131.6 (C-8), 134.0 (C-10), 145.1 (C-2)* (C-2 was assigned by HMBC data) , 153.7 (C-3), 160.2 (C-12), 167.6 (13-C=O); δ_F -133.19 (1F, d, *J* 1.5, F-2). HRCI-MS *m/z* 341.0967 [M]H^+ (calcd. for $C_{17}H_{19}FClO_4$, 341.0956); *m/z* (ESI) 363/365 (MNa^+, MNa^++ 2 (^{37}Cl), 40%/14%), 341/343 (MH^+, MH^+ + 2 (^{37}Cl), 79%/ 29%) 309/311 (MH^+- MeOH, MH^+- MeOH + 2 (^{37}Cl), 30%/ 10%).

3-Fluorocinnamic Acid

Following the above procedure 3-fluorocinnamic acid (150 mg·L^{-1}) was fed to *B. lutea*. Fluorine-enriched metabolites were observed by preliminary LCMS analysis. The enriched metabolite 3-fluorostrobilurin A **36** was purified and its structure was confirmed by spectroscopic data. Other fluorinated strobilurins, 3-fluorostrobilurin B **55** and 3-fluorostrobilurin C **58** were only observed by LCMS analysis and could not be isolated due to very low yield for NMR confirmation.

3-Fluorostrobilurin A **36** pale yellow oil (16-22 mg·L^{-1} culture, inoculated with 150 mg of 3-fluorocinnamic acid): λ_{max} (MeOH) = 228, 299 nm; δ_H (500 MHz, $CDCl_3$), 1.98 (3H, br s, CH_3-14), 3.74 (3H, s, OCH_3-15), 3.85 (3H, s, OCH_3-16), 6.25 (1H, br d, *J* 10.8, H-9), 6.44 (1H, d, *J* 15.8, H-7), 6.60 (1H, dd, *J* 15.8, 10.8, H-8), 6.87 (1H, dddd, *J* 9.1, 8.1, 2.5, 0.8, H-4), 7.03 (1H, ddd, *J* 10.5, 2.5, 1.8 H-2), 7.10 (1H, dd, *J* 7.6, 1.8 H-6) 7.22 (1H, ddd, *J* 8.1, 7.6, 6.0 H-5), 7.44 (1H, s, H-12); δ_C (125 MHz, $CDCl_3$) 23.7 (14-CH_3), 51.6 (15-OCH_3), 61.9 (16-OCH_3), 110.6 (C-11), 111.4 (C-2), 113.8 (C-4), 122.26 (C-5), 127.8 (C-8), 129.3 (C-9), 129.9 (C-7), 132.6 (C-6), 140.2 (C-10), 140.3(C-1), 158.9 (C-12), 164.1 (C-3, d, *J* 156), 167.61 (13-C=O); δ_F -113.87 (ddd 9.5, 8.9 6.0 F-3). HRCI-MS *m/z* 277.1247 [M]H^+ (calcd. for $C_{16}H_{18}O_3F$, 277.1240); *m/z* (ESI) 299 (MNa^+, 100%), 277 (MH^+, 30%), 245 (MH^+- MeOH, 67%).

4-Fluorocinnamic Acid

Feeding of 4-fluorocinnamic acid (150 mg·L^{-1}) was carried out following the above procedures. 4-fluorostrobilurin A **51** was produced as shown by LCMS analysis. No other enriched strobilurin was observed. The enriched metabolite **51** was purified as pale yellow oil (10-14 mg·L^{-1} culture, inoculated with 150 mg of 4-fluorocinnamic acid): λ_{max} (MeOH) = 220, 290 nm; δ_H (500 MHz, $CDCl_3$), 1.97 (3H, br s, C**H_3-14**), 3.74 (3H, s, OC**H_3-15**), 3.85 (3H, s, OC**H_3-16**), 6.20 (1H, br d, *J* 10.8, **H-9**), 6.40 (1H, d, *J* 15.8, **H-7**), 6.50 (1H, dd, *J* 15.8, 10.8, **H-8**), 6.97 (2H, dd, *J* 9.0, 8.5, **H-3**,**5**), 7.30 (1H, dd, *J* 8.5, 5.4 **H-2**, **6**), 7.42 (1H, s, **H**-**12**); δ_C (100 MHz, $CDCl_3$) 23.7 (**14**-**C**H_3), 51.6 (**15**-OCH_3), 62.2 (**16**-O**C**H_3), 111.6 (**C-11**), 116.7 (**C**-**3**), 116.9 (**C-5**), 128.6 (**C**-**2**), 128.6 (**C**-**6**), 129.7 (**C-8**), 130.2 (**C-1)**, 130.9 (**C-9**), 131.8 (**C-7**), 136.2 (**C-10**), 140.2 (**C-1**), 159.8 (**C-12**), 163.6 (**C-4**), 167.61 (**13-C=**O); δ_F -113.87 (1F, tt, *J* 9.3, 5.5 **F-4**). HRCI-MS *m/z* 277.1247 [M]H^+ (calcd. for $C_{16}H_{18}O_3F$, 277.1240); *m/z* (ESI) 299 (MNa^+, 40%), 277 (MH^+ 50%), 245 (MH^+- MeOH, 100%).

Nicotinic Acid

Feeding of nicotinic acid acid (50 mg·L^{-1}) was carried out following the above procedures. 3-aza-strobilurin A **60** was produced as shown by LCMS analysis. The enriched metabolite **60** was purified as pale yellow oil (10 mg·L^{-1} culture, inoculated with 50 mg of nicotinic acid): λ_{max} (MeOH) = 224, 290 nm; δ_H (500 MHz, $CDCl_3$), 1.94 (3H, br s, C**H_3-14**), 3.71 (3H, s, OC**H_3-15**), 3.86 (3H, s, OC**H_3-16**), 6.95 (1H, br d, *J* 10.4, **H-9**), 7.29 (1H, d, *J* 15.6, **H-7**), 7.45 (1H, dd, *J* 15.6, 10.4, **H-8**), 7.98 (1H, t, *J* 6.0, **H-3**), 8.11 (1H, d, *J* 6.0, **H-2**), 8.12 (1H, s, **H**-**6**), 8.25. 1H, d, *J* 6.0, **H-4**), 8.28 (1H, s, **H-12**). *m/z* (ESI) 282 (MNa^+, 35%), 260 (MH^+, 45%), 228 (MH^+- MeOH, 70%). 168 (M^+- CO_2Me, -MeOH, 100%).

5.18 Feeding and Incorporation of 4-Methylhexa-2,4-dienoic Acid 61

Following the standard feeding procedure 15 mg of 4-methylhexa-2, 4-dienoic acid **61**, was fed to 150 mL culture of *B. lutea* in 500 mL flask. All the enriched flasks were incubated at 25 °C and 150 rpm for 7-10 days. The resulting new metabolite, diol **62** was purified by preparative HPLC using methods as previously described

Diol **62**: λ_{max} (MeOH) = 232 nm; δ_H (500 MHz, $CDCl_3$), 1.15 (3H, d, *J* 6.36 C**H_3-1**), 1.71 (3H, d, *J* 6.2 CH_3-8), 1.76 (3H, s, CH_3-9), 3.90 (1H, m, H-2), 4.12 (1H, dd, *J* 7.4, 3.9 H-3) 5.58 (1H, dd, *J* 15.6, 7.4 H-4), 5.61 (1H, q, *J* 6.2 H-7) 6.31 (1H, d, *J* 15.6, H-5); δ_C (125 MHz) 12.1 (C-9), 13.9 (C-8), 17.7 (C-1), 70.3 (C-2), 86.5 (C-3), 123.3 (C-4), 128.3 (C-7), 133.7 (C-6), 138.51 (C-5). HRESI-MS *m/z* 179.1050 [M]Na^+ (calcd. for $C_9H_{16}O_2Na^+$, 179.1042); *m/z* (ESI) 179 (MNa^+, 100%), 157 (10%), 139 (20%).

2. Synthesis of intermediates

General Synthetic and Analytical Details

Commercially available compounds were used without further purification except where stated. Experiments which included moisture or air sensitive reactions were carried out in flame-dried glassware under a positive pressure of nitrogen using standard syringe/ septa techniques. Anhydrous solvents dichloromethane and tetrahydrofuran were obtained by passing through a modified Grubbs system of alumina columns, manufactured by Anhydrous Engineering. Petroleum ether is of the 40-60 °C boiling point range. Routine monitoring of reactions was performed using precoated Merck-Keiselgel 60 F_{254} aluminium backed T.L.C. plates. The spots were visualised by UV_{254} light, or potassium permanganate. Flash column chromatography was performed using silica gel (40-63 micron, obtained from Fluorochem Ltd.) as the adsorbent and carried out according to the procedure outlined by Still *et al.*[1] Melting points were determined on an Electrothermal IA6301 melting point apparatus and are uncorrected. Infrared (IR) spectra were recorded on a Perkin Elmer Spectrum One FT-IR spectrometer as either a neat solid or liquid. ^1H, ^{13}C and CRAPT NMR spectra were recorded as solution in $CDCl_3$ unless stated otherwise. The spectra were recorded on a lambda 300 MHz, varian 400 MHz or a Jeol Eclipse 400 MHz spectrometer. The chemical shifts (δ) are reported in parts per million (ppm) and the coupling constants (*J*) are in Hertz (Hz). Electrospray (ESI) mass spectra were recorded on a Bruker Daltonics Apex 4e 7.0T FT-MS mass spectrometer. Methane was the ionised gas used for the chemical ionisation. Unless stated, data for all known compounds are in agreement with published data.

Ethyl 2-methyl-3-oxobutanoate

Ethyl acetoacetate (5 mL, 39.534 mmol) was added dropwise to a mixture of THF (30 mL) and NaH (60%, 1.7 g, 42.500 mmol, pre-washed with *n*-hexane) at 0 °C under an atmosphere of nitrogen. Iodomethane (2.3 mL, 36.945 mmol) was added to stir for 0.5 h at room temperature and then the mixture was stirred for 20 h at 50 °C. Water (40 mL) was added to quench the reaction and the aqueous layer was extracted with EtOAc (3 × 40 mL). The organic layers were combined, dried over $MgSO_4$, filtered and concentrated *in vacuo*. The crude oil was purified by flash chromatography (SiO_2, 10% EtOAc in petroleum ether 40-60 °C) giving ethyl 2-methyl-3-oxobutanoate as a colourless oil (3.207 g, 56%). δ_H(400 MHz, $CDCl_3$) 1.28 (3H, t, *J* 7.1, OCH_2CH_3), 1.35 (3H, d, *J* 7.1, 2-CH_3), 2.25 (3H, s, 4-H_3), 3.50 (1H, q, *J* 7.3, 2-H), 4.21 (2H, q, *J* 7.1, OCH_2CH_3); δ_C(100 MHz, $CDCl_3$) 12.7 (2-CH_3), 14.1 (OCH_2CH_3), 28.4 (C-4), 53.6 (C-2), 61.3 (OCH_2CH_3), 170.5 (C-1), 203.6 (C-3). Spectroscopic data in accord with literature.[2]

(2*Z*,4*E*)-5-(3'-Fluorophenyl)-2-methylpenta-2,4-dienoic acid (26)

Diisopropylamine (5.81 mL, 41.17 mmol) was dissolved in anhydrous THF (35 mL) and then cooled to 0 °C under an atmosphere of nitrogen. *n*-BuLi (2.5 M in hexane, 16.47 mL, 41.17) was added dropwise followed by adding HMPA (2.87 mL, 16.47 mL) dropwise to the solution. The mixture was cooled to –78 °C and then ethyl 2-methyl-3-oxobutanoate (2.374 g, 16.47 mmol) in anhydrous THF (5 mL) was added. After the reaction was stirred for 1 h, 3-fluorobenzaldehyde (2.25 mL, 18.12 mmol) was added and stirred for 2 h. The reaction was

quenched by $HCl_{(aq)}$ (6 M, 20 mL) and then allowed to warm to room temperature. The aqueous layer was extracted into Et_2O (3 × 60 mL). The organic layers were combined, dried over $MgSO_4$, filtered and concentrated *in vacuo*. The crude oil was used in the next step without purification. The crude oil was diluted with $KOH_{(aq)}$ (1 M, 80 mL) and stirred for 14 h. The mixture was cooled to 0 °C and acidified with $HCl_{(aq)}$ (6 M) to pH 0, and a yellow solid was precipitated from the solution. The solid was filtered and washed with water (50 mL). The aqueous layer was extracted into Et_2O (3 × 60 mL). The organic layers were combined, dried over $MgSO_4$, filtered and concentrated *in vacuo*. The yellow solid collected from the filtrate and the concentrated residue was recrystallised from MeOH giving lactone **25** as a white solid (2.388 g, 65%). m.p. 174-176 °C; ν_{max}/cm^{-1} 2925, 2608, 1595, 1490; δ_H(400MHz, DMSO) 1.68 (3H, s, 3-CH_3), 2.65 (1H, m, 5-*H*H), 2.87 (1H, m, 5-H*H*), 5.44 (1H, dd, *J* 12.1, 3.9, 6-H), 7.20-7.46 (4H, m, ArH), 10.84 (1H, br s, OH); δ_C(100 MHz, DMSO) 8.7 (3-CH_3), 34.5 (C-5), 74.5 (C-6), 97.3 (C-3), 113.2, 115.0, 122.4, 130.6, 142.1 and 162.1 (Ar), 165.4 and 167.7 (C-2 and C-4). Found (CI): 223.0780 $[M+H]^+$, (required $C_{12}H_{12}FO_3$ 223.0770). Anal. Calcd. for $C_{12}H_{11}FO_3$: C, 64.86; H, 4.99. Found: C, 64.75; H, 4.88.

Lactone **25** (0.892 g, 4.015 mmol) was dissolved in anhydrous CH_2Cl_2 (20 mL) and cooled to –78 °C under an atmosphere of nitrogen. *N*,*N*-Diisopropylethylamine (1.05 mL, 6.022 mmol) in anhydrous CH_2Cl_2 (5 mL) was added dropwise and stirred for 20 minutes. Triflic anhydride (0.75 mL, 4.458 mmol) in anhydrous CH_2Cl_2 (5 mL) was added dropwise over 5 minutes. The reaction was stirred for 30 minutes and then concentrated *in vacuo* giving a residue which was diluted with Et_2O (20 mL). The organic layer was washed with cold $HCl_{(aq)}$ (6 M, 2 × 5 mL) and brine (10 mL). The organic layer was dried over $MgSO_4$, filtered and concentrated *in vacuo*. The crude solid was purified by flash chromatography (SiO_2, 10% EtOAc in petroleum ether 40-60 °C) giving the triflate as a white solid (1.381 g, 97%). m.p. 64-66 °C; ν_{max}/cm^{-1} 1716, 1687, 1596, 1490; δ_H(400 MHz, $CDCl_3$) 2.05 (3H, dd, *J* 2.5, 1.2, 3-CH_3), 2.92 (1H, m, 5-*H*H), 3.12 (1H, m, 5-H*H*), 5.50 (1H, dd, *J* 11.9, 4.0, 6-H), 7.09-7.40 (4H, m, ArH); δ_C(100 MHz, $CDCl_3$) 10.8 (3-CH_3), 34.7 (C-5), 76.3 (C-6), 113.1 and 116.2 (Ar), 119.8 (CF_3), 120.8 (C-3), 121.5, 130.6 and 139.1 (Ar), 154.8 (C-4), 162.9 (Ar), 164.1 (C-2). Found (CI): 355.0272 $[M+H]^+$, (required $C_{13}H_{11}F_4O_5S$ 355.0263). Anal. Calcd. for $C_{13}H_{10}F_4O_5S$: C, 44.07; H, 2.85. Found: C, 44.35; H, 2.96.

The triflate (1.114 g, 3.143 mmol) was dissolved in DMF (16 mL), and then tetrakis(triphenylphospine)palladium (0.036 g, 0.031 mmol) and triethylsilane (1.004 mL, 6.287 mmol) were added to the solution. The mixture was heated to 60 °C and stirred for 2 h. The mixture was cooled to room temperature and water (10 mL) was added. The aqueous layer was extracted into EtOAc (3 × 50 mL). The organic layers were combined, extracted into brine (10 mL), dried over $MgSO_4$, filtered and concentrated *in vacuo*. The crude solid was purified by flash chromatography (SiO_2, 20% EtOAc in petroleum ether 40-60 °C) giving lactone as a yellow oil (0.639 g, 99%). ν_{max}/cm^{-1} 2985, 1714, 1648, 1449, 1360; δ_H(400MHz, $CDCl_3$) 2.0 (3H, m, 3-CH_3), 2.60 (2H, m, 5-H_2), 5.42 (1H, dd, *J* 11.0, 5.1, 6-H), 6.67 (1H, m, 4-H), 7.05-7.37 (4H, m, ArH); δ_C(100 MHz, $CDCl_3$) 17.0 (3-CH_3), 31.9 (C-5), 78.4 (d, *J* 2.3, C-6), 113.1, 115.3 and 121.5 (Ar), 128.9 (C-3), 130.2 (Ar), 138.50 (C-4), 141.2 (d, *J* 6.9, C-7), 162.8 (Ar), 165.3 (C-2). Found (EI): 206.0735 $[M]^+$, (required $C_{12}H_{11}FO_2$ 206.0743).

The lactone (0.639 g, 3.101 mmol) was dissolved in anhydrous THF (100 mL) and then TBAF (1 M in THF, 15.5 mL, 15.5 mmol) was added under an atmosphere of nitrogen. The mixture was stirred for 16 h following by adding water (100 mL). The aqueous layer was extracted with EtOAc (3 × 100 mL). The organic layers were combined, dried over $MgSO_4$, filtered and concentrated *in vacuo*. The crude oil was purified by flash chromatography (SiO_2, 60% EtOAc in petroleum ether 40-60 °C) giving dienoic acid **26** as white solid (0.621 g, 97%). m.p. 154-158 °C; ν_{max}/cm^{-1} 2929, 2577, 1719, 1657, 1595; δ_H(400MHz, CD_3COCD_3) 2.03 (3H, s, 2-CH_3), 6.71 (1H, d, *J* 11.2, 3-H), 6.81 (1H, d, *J* 15.6, 5-H), 7.05-7.41 (4H, m, ArH), 8.00 (1H, dd, *J* 15.6, 11.2, 4-H); δ_C(100 MHz, CD_3COCD_3) 21.3 (2-CH_3), 113.9, 115.8 and 123.9 (Ar), 128.5 (C-4),

128.9 and131.5 (Ar), 137.0 (d, *J* 3.0, C-5), 140.4 (C-3), 140.7 (d, *J* 7.8, C-2), 164.1 (Ar), 168.7 (C-1); Found (CI): 207.0823 $[M+H]^+$, (required $C_{12}H_{12}FO_2$ 207.0821); Anal. Calcd. for $C_{12}H_{11}FO_2$: C, 69.89; H, 5.38. Found: C, 70.00; H, 5.47.

S-(2-Acetamidoethyl) (2*Z*,4*E*)-5-(3-fluorophenyl)-2-methylpenta-2,4-dienethioate (27)

Dienoic acid **26** (0.062 g, 0.302 mmol) was dissolved in anhydrous CH_2Cl_2 (5 mL) under an atmosphere of nitrogen. EDCI (0.095 g, 0.496 mmol) and DMAP (0.041 g, 0.362 mmol) were added to the solution and cooled to 0 °C. N-acetylcysteamine (0.054 g, 0.453 mmol) in anhydrous CH_2Cl_2 (1.5 mL) was added to the mixture and stirred at 0 °C for 2 h then at room temperature for 14 h. The reaction was quenched by adding saturated $NH_4Cl_{(aq)}$ (2 mL) and the aqueous layer was extracted into CH_2Cl_2 (3 × 10 mL). The organic layers were combined, dried over $MgSO_4$, filtered and concentrated *in vacuo*. The crude product was purified by flash chromatography (SiO_2, 80% EtOAc in petroleum ether 40-60 °C) giving thiol ester **(27)** as a white solid (0.087 g, 94%). m.p. 104-105 °C; ν_{max}/cm^{-1} 3304, 3073, 2924, 1633, 1583, 1546, 1488, 1442; δ_H(400 MHz, $CDCl_3$) 2.03 (3H, s, $NCOCH_3$), 2.19 (3H, s, 2-CH_3), 3.18 (2H, t, *J* 6.3, SCH_2), 3.54 (2H, app. q, *J* 6.3, NCH_2), 6.24 (1H, br s, NH), 6.44 (1H, m, 3-H), 6.73 (1H, d, *J* 15.4, 5-H), 7.01-7.33 (4H, m, ArH), 7.83 (1H, dd, *J* 15.7, 11.3, 4-H); δ_C(100 MHz, $CDCl_3$) 20.7 (2-CH_3), 23.1 (NCO*C*H_3), 28.5 (SCH_2), 39.5 (NCH_2), 113.3, 115.3, 123.0, 126.6 and 130.1 (Ar), 132.4 (C-3), 137.6 (C-4), 138.3 (d, *J* 3.1, C-5), 138.8 (d, *J* 7.0, C-2), 162.9 (d, *J* 245.9, Ar), 170.3 (C-1), 192.9 (NCO). Found (CI): 308.1110 $[M+H]^+$, (required $C_{16}H_{19}FNO_2S$ 308.1121). Anal. Calcd. for $C_{16}H_{18}FNO_2S$: C, 62.52; H, 5.90; N, 4.56. Found: C, 62.37; H, 6.16; N, 4.79.

(2*Z*,4*E*)-5-(3-Fluorophenyl)-2-methylpenta-2,4-dien-1-ol (28)

Dienoic acid **26** (0.057 g, 0.276 mmol) was dissolved in anhydrous THF (1 mL) and cooled to 0 °C under an atmosphere of nitrogen. Triethylamine (0.077 mL, 0.552 mmol) and ethyl chloroformate (0.034 mL, 0.359 mmol) were added. The mixture was stirred at 0 °C for 0.5 h and then filtered through Celite plug to remove the solid which was washed with EtOAc (3 mL). The filtrate was concentrated *in vacuo* and then diluted with MeOH (2 mL). The yellow solution was cooled to –78 °C and $NaBH_4$ (0.026 g, 0.690 mmol) was added in portions and stirred at –78 °C for 4 h. Saturated $NH_4Cl_{(aq)}$ (2 mL) was added and the aqueous layer was extracted into EtOAc (3 × 10 mL). The combined organic layers were washed with brine (5 mL), dried over $MgSO_4$, filtered and concentrated *in vacuo*. The crude solid was purified by flash chromatography (SiO_2, 10% EtOAc in petroleum ether 40-60 °C) giving alcohol **28** as a white solid (0.032 g, 60%). m.p. 80-84 °C; ν_{max}/cm^{-1} 3305, 3071, 2923, 1654, 1631; δ_H(400MHz, $CDCl_3$) 1.99 (3H, s, 2-CH_3), 4.40 (2H, s, 1-H_2), 6.14 (1H, m, 3-H), 6.49 (1H, d, *J* 15.4, 5-H), 6.92 (1H, m, ArH), 7.07 (1H, dd, *J* 15.4, 11.2, 4-H), 7.10-7.27 (3H, m, ArH); δ_C(100MHz, $CDCl_3$) 21.8 (2-CH_3), 61.9 (C-1), 112.6 (Ar), 114.2 (Ar), 122.2 (Ar), 125.2 (C-3), 127.8 (C-4), 130.0 (Ar), 130.7 (d, *J* 3.1, C-5), 138.8 (Ar), 139.8 (d, *J* 7.7, C-2), 163.1 (Ar). Found (CI): 193.1026 $[M+H]^+$, (required $C_{12}H_{14}FO$ 193.1029). Anal. Calcd. for $C_{12}H_{14}FO$: C, 74.98; H, 6.82. Found: C, 74.64; H, 6.75.

Ethyl (2*E*,4*Z*,6*E*)-7-(3-fluorophenyl)-4-methylhepta-2,4,6-trienoate (29)

1. Alcohol **28** (0.198 g, 1.028 mmol) was dissolved in anhydrous CH_2Cl_2 (14 mL) at room temperature under an atmosphere of nitrogen. Dess-Martin periodinane (15 wt%, 2.78 mL, 1.336 mmol) added and the mixture stirred for 1.5 h. The reaction was quenched with water (5 mL) and the aqueous layer was extracted into CH_2Cl_2 (3 × 20 mL). The organic layers were combined, dried over $MgSO_4$, filtered and concentrated *in vacuo* to give the crude aldehyde as a colourless oil.

2. NaH (60%, 0.058 g, 1.439 mmol) was prewashed by hexane (2 × 2 mL) and suspended in anhydrous THF (40 mL) under an atmosphere of nitrogen. Triethyl phosphonoacetate (0.49 mL, 2.467 mmol) was added to the mixture and cooled to 0 °C and stirred for 10 min. The crude aldehyde in anhydrous THF (3 mL) was added to the reaction dropwise then stirred at room

temperature overnight. Brine (17 mL) was added and the aqueous layer was extracted into EtOAc (3 × 50 mL). The combined organic layers were washed with brine (20 mL), dried over $MgSO_4$, filtered and concentrated *in vacuo*. The crude solid was purified by flash chromatography (SiO_2, 10% EtOAc in petroleum ether 40-60 °C) giving ester **29** as a yellow solid (0.180 g, 67%). ν_{max}/cm^{-1} 3426, 2984, 2933, 1716; m.p. 60-62 °C; δ_H(400 MHz, $CDCl_3$) 1.35 (3H, t, *J* 7.1, OCH_2CH_3), 2.00 (3H, s, 4-CH_3), 4.28 (2H, q, *J* 7.1, OCH_2CH_3), 5.97 (1H, d, *J* 15.4, 2-H), 6.42 (1H, m, 5-H), 6.60 (1H, d, *J* 15.3, 7-H), 6.96-7.30 (4H, m, ArH), 7.34 (1H, dd, *J* 15.3, 11.5, 6-H), 8.01 (1H, d, *J* 15.4, 3-H); δ_C(100 MHz, $CDCl_3$) 14.3 (OCH_2CH_3), 20.3 (4-CH_3), 60.5 (OCH_2CH_3), 113.1 and 114.9 (Ar), 118.9 (C-5) 122.6 (d, *J* 3.1, C-13), 124.6 (C-6), 130.1 and 133.0 (Ar), 133.9 (d, *J* 2.3, C-7), 136.4 (C-2), 139.3 (d, *J* 7.8, C-4), 139.9 (C-3), 163.1 (Ar), 167.3 (C-1). Found (CI): 261.1282 $[M+H]^+$, (required $C_{16}H_{18}FO_2$ 261.1291). Anal. Calcd. for $C_{16}H_{18}FO_2$: C, 73.83; H, 6.58. Found: C, 74.04; H, 7.04.

(2*E*,4*Z*,6*E*)-7-(3-Fluorophenyl)-4-methylhepta-2,4,6-trienoic acid (30)

Ester **29** (0.180 g, 0.692 mmol) was dissolved in THF (36 mL) and $NaOH_{(aq)}$ (1 M, 72 mL) added then stirred at room temperature for 16 h. The reaction was cooled to 0 °C and $HCl_{(aq)}$ (6 M) was added to the reaction until pH 2. The aqueous layer was extracted into EtOAc (3 × 50 mL) and the organic layers combined, dried over $MgSO_4$, filtered and concentrated *in vacuo*. The crude product was purified by flash chromatography (SiO_2, 60% EtOAc in petroleum ether 40-60 °C) giving trienoic acid **30** as a yellow solid (0.147 g, 91%). m.p. 179-180 °C; ν_{max}/cm^{-1} 2884, 2563, 1695, 1669, 1615; δ_H(400 MHz, $CDCl_3$) 2.03 (3H, s, 4-CH_3), 5.98 (1H, d, *J* 15.4, 2-H), 6.55 (1H, d, *J* 11.5, 5-H), 6.77 (1H, d, *J* 15.4, 7-H), 7.03-7.47 (4H, m, ArH), 7.65 (1H, dd, *J* 15.4, 11.5, 6-H), 8.05 (1H, d, *J* 15.4, 3-H); δ_C(100 MHz, $CDCl_3$) 20.4 (4-CH_3), 113.8 (d, *J* 21.8, C-9), 115.5 (Ar), 120.1 (C-5), 124.1 (Ar), 126.0 (C-6), 131.4 and 133.4 (Ar), 134.1 (C-4), 135.0 (d, *J* 3.1, C-7), 137.5 (C-2), 141.0 (C-3), 143.2 and 164.2 (Ar), 168.0 (C-1); Found (ESI): 255.0779 $[M+Na]^+$ (required $C_{14}H_{13}FO_2Na$ 255.0792). Anal. Calcd. for $C_{14}H_{13}FO_2$: C, 72.40; H, 5.64. Found: C, 72.31; H 5.77.

***S*-(2-Acetamidoethyl) (2*E*,4*Z*,6*E*)-7-(3-fluorophenyl)-4-methylhepta-2,4,6-trienethioate (31)**

Trienoic acid **30** (0.025 g, 0.109 mmol) was dissolved in anhydrous CH_2Cl_2 (1 mL). EDCI (0.042 g, 0.218 mmol) was added to the solution followed by DMAP (0.030 g, 0.261 mmol) at 0 °C under an atmosphere of nitrogen. HSNAC (0.116 g, 0.973 mmol) in anhydrous CH_2Cl_2 (1 mL) was added to the mixture and stirred at room temperature for 16 h. The reaction was quenched with saturated $NH_4Cl_{(aq)}$ (5 mL) and the aqueous layer was extracted into CH_2Cl_2 (3 × 10 mL). The organic layers were combined, dried over $MgSO_4$, filtered and concentrated *in vacuo*. The crude product was purified by flash chromatography (SiO_2, 60% EtOAc in petroleum ether 40-60 °C) giving thiol ester **31** as a yellow solid (0.022 g, 60%). ν_{max}/cm^{-1} 3300, 3073, 2912, 2850, 1649, 1582; m.p. 130-132 °C; δ_H(400 MHz, $CDCl_3$) 2.00 (3H, s, 4-CH_3), 2.01 (3H, s, $NHCOCH_3$), 3.18 (2H, m, SCH_2), 3.52 (2H, m, $NHCH_2$), 5.95 (1H, br s, NH), 6.25 (1H, d, *J* 15.2, 2-H), 6.53 (1H, d, *J* 11.5, 5-H), 6.64 (1H, d, *J* 15.4, 7-H), 6.98-7.24 (3H, m, ArH), 7.30 (1H, dd, *J* 5.9, 2.0, 6-H), 7.34 (1H, m, ArH), 7.95 (1H, d, *J* 15.2, 3-H); δ_C(100 MHz, $CDCl_3$) 20.1 ($NCOCH_3$), 23.3 (4-CH_3), 28.6 (SCH_2), 39.9 ($NHCH_2$), 113.2, 115.2 and 122.7 (Ar), 124.4 (C-6), 125.0 (C-2), 130.2 and 132.6 (Ar), 134.8 (d, *J* 3.1, C-7), 136.3 (C-3), 138.6 (C-5), 139.1 (d, *J* 7.8, C-4), 163.2 (Ar), 170.3 (C-1), 190.3 (CON). Found (ESI): 356.1088 $[M+Na]^+$ (required $C_{18}H_{20}FNSO_2Na$ 356.1091). Anal. Calcd. for $C_{18}H_{20}FNSO_2$: C, 64.84; H, 6.05; N, 4.20. Found: C, 64.50; H 6.58; N, 4.46.

Ethyl (2*Z*,4*Z*,6*E*)-7-(3-fluorophenyl)-4-methylhepta-2,4,6-trienoate (32)

1. Alcohol **28** (0.072 g, 0.373 mmol) was dissolved in anhydrous CH_2Cl_2 (5.4 mL) and Dess-Martin periodinane (15 wt%, 1.0 mL, 1.485 mmol) added under an atmosphere of nitrogen in the dark. The mixture was stirred for 1.5 h at room temperature then water (3 mL) added. The aqueous phase was extracted into DCM (3 × 20 mL) and the combined organic layers were dried over $MgSO_4$, filtered and concentrated *in vacuo*. The crude aldehyde was used to the next step without further purification.

2. Ethyl [bis(2,2,2-trifluoroethoxy)phosphinyl]acetate (0.185 mL, 0.783 mmol) and 18-crown-6 (0.237 g, 0.897 mmol) were dissolved in anhydrous THF (2.3 mL) under an atmosphere of nitrogen. The mixture was cooled to –78 °C and KHMDS (15 wt% in toluene, 1.45 mL, 0.746 mmol) was added dropwise and stirred for 0.5 h. Aldehyde from the last step in anhydrous THF (3 mL) was added and stirred for 6 h at –78 °C. Saturated $NH_4Cl_{(aq)}$ (5 mL) was added to the reaction and the aqueous phase was extracted into Et_2O (3 × 30 mL). The combined organic layers were dried over $MgSO_4$, filtered and concentrated *in vacuo*. The crude oil was purified by flash chromatography (SiO_2, 10% EtOAc in petroleum ether 40-60 °C) giving ester **32** (2*Z*:2*E*::5:1) as a colourless oil (0.062 g, 64%). The following analysis was based on the major component **32**. ν_{max}/cm^{-1} 2958, 2871, 1670, 1621; δ_H(400 MHz, $CDCl_3$) 1.31 (3H, t, *J* 7.1, OCH_2CH_3), 2.10 (3H, d, *J* 1.2, 4-CH_3), 4.21 (2H, q, *J* 7.1, OCH_2CH_3), 5.74 (1H, d, *J* 12.7, 2-H), 6.42-6.45 (1H, m, 5-H), 6.46 (1H, d, *J* 12.7, 3-H), 6.62 (1H, d, *J* 15.4, 7-H),6.94 (1H, m, ArH), 7.09 (1H, dd, *J* 15.4, 11.2, 6-H), 7.14-7.29 (3H, m, ArH); δ_C(100 MHz, $CDCl_3$) 14.1 (OCH_2CH_3), 15.6 (4-CH_3), 60.3 (OCH_2CH_3), 112.8 and 114.6 (Ar), 118.2 (C-2), 122.5 (Ar), 125.8 (C-6), 130.0 (Ar), 133.9 (d, *J* 3.1, C-7), 135.3 (C-4), 135.9 (C-5), 139.5 (Ar), 144.5 (C-3), 163.1 (Ar), 166.7 (C-1). Found (CI): 261.1295 $[M+H]^+$ (required $C_{16}H_{18}FO_2$ 261.1291).

(2*Z*,4*Z*,6*E*)-7-(3-Fluorophenyl)-4-methylhepta-2,4,6-trienoic acid (33)

Ester **32** (2*E*:2*Z*::1:5) (0.109 g, 0.417 mmol) was dissolved in MeOH (2 mL) and $LiOH_{(aq)}$ (1 M, 4 mL) was added. The reaction was stirred overnight and then acidified by $HCl_{(aq)}$ (6 M, 10 mL). The aqueous phase was extracted into EtOAc (3 × 30 mL) and the combined organic layers were dried over $MgSO_4$, filtered and concentrated *in vacuo*. The crude oil was purified by flash chromatography (SiO_2, 20% EtOAc in petroleum ether 40-60 °C) giving acid **33** (2*E*:2*Z*::1:5) (0.070 g, 72%) as a colourless oil. δ_H(400 MHz, $CDCl_3$) 2.14 (3H, d, *J* 1.0, 4-CH_3), 5.77 (1H, d, *J* 12.6, 2-H), 6.49 (1H, d, *J* 11.4, 5-H), 6.61 (1H, d, *J* 12.6, 3-H), 6.66 (1H, d, *J* 15.5, 7-H), 6.96 (1H, m, ArH), 7.10 (1H, dd, *J* 15.5, 11.4, 6-H), 7.14-7.30 (3H, m, ArH); δ_C(100 MHz, $CDCl_3$) 15.9 (4-CH_3), 113.0 and 114.9 (Ar), 116.9 (C-2), 122.7 (Ar), 125.8 (C-6), 130.1 (Ar), 134.7 (d, *J* 2.3, C-7), 135.2 (C-4), 137.1 (C-5), 139.4 (Ar), 147.7 (C-3), 163.1 (Ar), 166.7 (C-1). Found (ESI): 255.0801 $[M+Na]^+$ (required $C_{14}H_{13}FO_2Na$ 255.0792).

Methyl (4*Z*,6*E*)-2,3-epoxy-7-(3-fluorophenyl)-4-methylhept-4,6-dienoate (34)

1. Alcohol **28** (0.079 g, 0.410 mmol) was dissolved in anhydrous CH_2Cl_2 (6 mL) under an atmosphere of nitrogen. Dess-Martin periodinane (15 wt %, 1.1 mL, 0.534 mmol) was added to the solution and the reaction was stirred for 1 h at room temperature. The reaction was quenched by adding water (3 mL) and the aqueous phase was washed with CH_2Cl_2 (3 × 30 mL). The organic layers were combined, dried over $MgSO_4$, filtered and concentrated *in vacuo*.

2. Diisopropylamine (0.12 mL, 0.821 mmol) was dissolved in anhydrous THF (1 mL) and was cooled to –78 °C followed by adding *n*-BuLi (2.28 M in hexane, 0.36 mL, 0.821 mmol) dropwise under an atmosphere of nitrogen. The mixture was stirred for 1 h and then a mixture of methyl bromoacetate (0.08 mL, 0.821 mmol) and indium (III) chloride (0.054 g, 0.246 mmol) in anhydrous THF (1 mL) was added dropwise. The reaction was stirred for 10 min at -78 °C. Aldehyde from part 1 in anhydrous THF (1 mL) was added dropwise and the reaction was stirred at –78 °C for 1 h and then at room temperature for 1 h. The reaction was quenched by adding water (5 mL) and the aqueous phase was washed with EtOAc (3 × 30 mL). The organic layers were combined, dried over $MgSO_4$, filtered and concentrated *in vacuo*. The crude oil was purified by flash chromatography (SiO_2, 10% EtOAc in petroleum ether 40-60 °C) giving epoxide **34** as a yellow oil (0.070 g, 65%). ν_{max}/cm^{-1} 2955, 1754, 1445; 2,3-H_2 in **34** are *anti*: δ_H(400 MHz, $CDCl_3$) 1.73 (3H, s, 4-CH_3), 3.61 (1H, d, *J* 2.0, 2-H), 3.86 (3H, s, OCH_3), 4.14 (1H, d, *J* 2.0, 3-H), 6.37 (1H, m, 5-H), 6.53 (1H, d, *J* 15.4, 7-H), 6.95-7.30 (5H, m, 6-H and ArH); δ_C(100 MHz, $CDCl_3$) 17.7 (4-CH_3), 52.3 (OCH_3), 52.6 (C-2), 56.2 (C-3), 112.8, 114.6 and 122.4 (Ar), 124.0 (C-6), 129.2 (C-5), 130.0 and 132.8 (Ar and C-7), 139.4 (d, *J* 7.7, C-4), 163.1 (Ar), 169.3 (C-1); 2,3-H_2 in **34** are

syn: δ_H(400 MHz, $CDCl_3$) 1.93 (3H, s, 4-CH_3), 3.67 (3H, s, OCH_3), 3.85 (1H, d, *J* 4.4, 2-H), 3.94 (1H, d, *J* 4.4, 3-H), 6.17 (1H, m, 5-H), 6.42 (1H, d, *J* 15.4, 7-H), 6.94-7.30 (5H, m, 6-H and ArH); δ_C(100 MHz, $CDCl_3$) 20.5 (4-CH_3), 52.1 (OCH_3), 54.2 (C-2), 56.6 (C-3), 112.6, 114.3 and 122.3 (Ar), 125.1 (C-6), 130.0 (d, *J* 8.5, C-5), 131.1 and 131.6 (Ar and C-7), 139.6 (d, *J* 7.7, C-4), 163.1 (Ar), 167.8 (C-1); Found (ESI): 285.0904 $[M+Na]^+$ (required $C_{15}H_{15}FO_3Na$ 285.0897).

_N_-Acetyl _S_-(2-bromoacetyl)cysteaminethioester

N-Acetylcysteamine (0.355 g, 2.980 mmol) was added to bromoacetyl bromide (0.42 mL, 4.768 mmol) and then the reaction was stirred under reduced pressure for 15 min. Saturated $NaHCO_{3(aq)}$ (10 mL) was added to the reaction and the aqueous phase was extracted into CH_2Cl_2 (3 × 20 mL). The combined organic layers were dried over $MgSO_4$, filtered and concentrated *in vacuo*. The crude oil was purified by flash chromatography (SiO_2, 100% EtOAc) giving the bromoacetylthioester as a colourless oil (0.519 g, 73%). δ_H(400 MHz, $CDCl_3$) 1.99 (3H, s, CH_3), 3.11, (2H, t, *J* 6.4, SCH_2), 3.47 (2H, app. q, *J* 6.4, NCH_2), 4.05 (2H, s, 2-H_2), 5.88 (1H, br s, NH); δ_C(100 MHz, $CDCl_3$) 23.2 (CH_3), 29.7 (SCH_2), 33.3 (C-2), 39.1 (NCH_2), 170.3 (NCO), 193.1 (C-1). Spectroscopic data in accord with the literature.[3]

Methyl (4*Z*,6*E*)-2,3-epoxy-7-(3-fluorophenyl)-4-methylhept-4,6-dienoate (35)

1. Alcohol **28** (0.089 g, 0.464 mmol) was dissolved in anhydrous CH_2Cl_2 (7 mL) under an atmosphere of nitrogen. Dess-Martin periodinane (15 wt%, 1.24 mL, 0.603 mmol) was added to the solution and the reaction was stirred for 1 h at room temperature. The reaction was quenched with water (3 mL) and the aqueous phase was extracted into CH_2Cl_2 (3 × 30 mL). The organic layers were combined, dried over $MgSO_4$, filtered and concentrated *in vacuo*.

2. Diisopropylamine (0.13 mL, 0.927 mmol) was dissolved in anhydrous THF (1 mL) and cooled to –78 °C followed by adding *n*-BuLi (2.5 M in hexane, 0.37 mL, 0.927 mmol) dropwise under an atmosphere of nitrogen. The mixture was stirred for 1 h and then a mixture of bromoacetyl thiol ester (0.223 g, 0.927 mmol) and indium (III) chloride (0.062 g, 0.278 mmol) in anhydrous THF (1 mL) was added dropwise. The reaction was stirred for 10 min at -78 °C under an atmosphere of nitrogen. Aldehyde in anhydrous THF (1 mL) was added dropwise and stirred at –78 °C for 1 h then at room temperature for 1 h. The reaction was quenched with water (5 mL) and the aqueous phase was extracted into EtOAc (3 × 20 mL). The organic layers were combined, dried over $MgSO_4$, filtered and concentrated *in vacuo*. The crude oil was purified by flash chromatography (SiO_2, 100% EtOAc) giving epoxy thiol ester **35** as a yellow oil (0.109 g, 67%). ν_{max}/cm^{-1} 3297, 3072, 2933, 1658; δ_H(400 MHz, $CDCl_3$) 1.71 (3H, s, 4-CH_3), 1.98 (3H, s, CH_3), 3.00-3.18 (2H, m, SCH_2), 3.38-3.57 (2H, m, NCH_2), 3.73 (1H, d, *J* 2.0, 2-H), 4.10 (1H, d, *J* 2.0, 3-H), 5.95 (1H, br s, NH), 6.36 (1H, d, *J* 11.5, 5-H), 6.52 (1H, d, *J* 15.4, 7-H), 6.92 (1H, tdd, *J* 8.3, 2.6, 0.9, ArH), 7.11-7.21 (3H, m, ArH), 7.27 (1H, td, *J* 8.1, 6.0, ArH); δ_C(100 MHz, $CDCl_3$) 17.7 (4-CH_3), 23.1 (CH_3), 28.1 (C-1′), 39.0 (C-2′), 57.9 (C-2), 59.1 (C-3), 112.8, 114.6 and 122.6 (Ar), 123.9 (C-6), 130.0 and 131.0 (Ar), 133.1 (d, *J* 3.1, C-7), 133.2 (C-5), 139.3 (d, *J* 7.8, C-4), 163.1 (Ar), 170.4 (NCO), 197.7 (C-1); Found (ESI): 350.1217 $[M+H]^+$ (required $C_{18}H_{21}FSNO_3$ 350.1221).

Ethyl 2-[$^2H_3$]-methyl-3-oxobutanoate

The preparation of 2-methyl-3-oxo-butanoate was repeated by using ethyl acetoacetate (0.90 mL, 7.149 mmol) and [$^2H_3$]-iodomethane (0.5 mL, 7.864 mmol) to afford the trideuterio-ester as a colourless oil (0.898 g, 85%). δ_H(400 MHz, $CDCl_3$) 1.28 (3H, t, *J* 7.1, OCH_2CH_3), 2.25 (3H, s, 4-H_3), 3.49 (1H, br s, 2-H), 4.21 (1H, qd, *J* 7.1, 1.0, OCH_2CH_3); δ_C(100 MHz, $CDCl_3$) 14.1 (OCH_2CH_3), 28.4 (C-4), 53.5 (C-2), 61.3 (OCH_2CH_3), 170.5 (C-1), 203.7 (C-3).

(2*E*,4*Z*,6*E*)-7-(3-Fluorophenyl)-4-[$^2H_3$]-methylhepta-2,4,6-trienoic acid

The preparation of **30** was repeated as above using ethyl 2-[$^2H_3$]-methyl-3-oxobutanoate (0.751 g, 5.105 mmol) to afford trideuterio-trienoic acid as a yellow solid (0.067 g, 72%).

δ_H(400 MHz, CD_3COCD_3) 5.98 (1H, d, *J* 15.4, 2-H), 6.54 (1H, d, *J* 11.5, 5-H), 6.77 (1H, d, *J* 15.4, 7-H), 7.03-7.45 (4H, m, ArH), 7.65 (1H, dd, *J* 15.4, 11.5, 6-H), 8.05 (1H, d, *J* 15.4, 3-H); δ_C(100 MHz, $CDCl_3$) 113.8 and 115.5 (Ar), 120.1 (C-5), 124.1 (Ar), 126.0 (C-6), 131.4 and 133.4 (Ar), 134.1 (C-4), 135.0 (d, *J* 3.1, C-7), 137.5 (C-2), 141.0 (C-3), 143.2 (Ar), 164.1 (Ar), 168.0 (C-1); Found (ESI): 258.0990 [MNa^+] (required $C_{14}H_{10}D_3FO_2Na$ 258.0980).

[2, 3-$^{13}C_2$]-cinnamic acid (23)

[2-^{13}C]-Malonic acid (0.50 g, 4.81 mmol), [1-13C]-benzaldehyde **192** (0.50 g, 4.72 mmol), pyridine (0.50 mL), piperidine (30 µL) and sodium sulphate (0.10 g, 0.70 mmol) were refluxed for 4 h. The solution was acidified with concentrated hydrochloric acid and the white precipitate which formed was dissolved by adding diethyl ether (40 mL). The organic phase was extracted with sodium hydroxide (2.0 M, 3 × 20 mL) and the combined aqueous extracts were acidified with concentrated HCl and then filtered to give [2,3-$^{13}C_2$]-cinnamic acid **23** (0.54 g, 87%) as white crystals. δ_H (400 MHz, $CDCl_3$), 6.48 (1H, ddd, *J* 163.0, 16.0, 1.0 2-H), 7.39-7.44 (3H, m, 3 × Ar-H), 7.54-7.59 (2H, m, 2 × Ar-H), 7.81 (1H, ddd, 156.5, 16.0, 2.5 H-3); δ_C (100 MHz) 117.3 (C-2, d, *J* 71.5 Hz), 128.6 (2 × Ar-C), 129.2 (2 × Ar-C), 130.8 (Ar-C), 134.0 (Ar-C_{ipso} d, *J* 48.0 Hz), 147.1 (C-3, d, *J* 71.5 Hz), 171.5 (C-1 d *J* 58.1 Hz); signal assigned to **C**-**2** (117.3 ppm) and **C**-**3** (147.1 ppm) appear as doublets (*J* 71.5) with an enhancement of 95% (based on NMR and MS data); *m/z* (EI) 151 [MH^+, 91%], 133 [48], 109 [57], 84 [30] and 63 [26].

[2, 3-$^{13}C_2$]-cinnamic acid *N*- Acetylcysteamine thiol ester (24)

Freshly prepared *N*-acetylcysteamine (0.27 g, 2.27 mmol) in dry CH_2Cl_2 (15 mL) was stirred at 0 °C under an atmosphere of nitrogen. DCC (0.36 g, 1.80 mmol) in dry CH_2Cl_2 (3 mL) was added followed by DMAP (0.009 g, 0.06 mmol). This was stirred for 10 minutes before addition of [2,3-$^{13}C_2$]-cinnamic acid **23** (0.21 g 1.59 mmol). The solution was left at 0 °C for 2 h and was allowed to warm to room temperature overnight. The reaction was subsequently quenched with saturated aq. Ammonium chloride solution (150 mL) and was extracted with CH_2Cl_2 (3 × 300 ml). The combined organic extracts were dried over magnesium sulphate. The solution was then filtered and concentrated *in vacuo* to yield a white solid. Ethyl acetate (5 mL) was added to dissolve the product and the insoluble urea by-product was then filtered off. The filtrate was then concentrated *in vacuo* to give the thiolester **24** (0.27 g 68%) as a shiny white solid. δ_H (400 MHz, $CDCl_3$),1.99 (3H, s, CH_3), 3.17 (2H, t, *J* 6.5, CH_2S), 3.51 (2H, q, *J* 6.5, CH_2N), 6.10 (1H, br s, NH), 6.73 (1H, ddd, *J* 161.0, 16.0, 1.5 H-2), 7.35-7.44 (3H, m, 3 × Ar-H), 7.53-7.59 (2H, m, 2 × Ar-H), 7.65 (1H, ddd, 155.5, 16.0, 2.5 H-3); δ_C (100 MHz) 23.3 ($COCH_3$), 28.6 (CH_2S), 39.8 (CH_2N), 124.7 (C-2 d, *J* 71.5), 128.5 (2 × Ar-C), 129.1 2 × Ar-C), 130.9 (Ar-C), 133.9 (Ar-C_{ipso} d, *J* 56.0 Hz), 141.3 (C-3 d, *J* 71.5), 170.5 (CON), 190.2 (C-1 d *J* 63.2 Hz), signals assigned to C-2 (124.7 ppm) and C-3 (141.3 ppm) appear as doublets (*J* 71.5) with an enhancement of > 90% (based on NMR and MS data.); *m/z* (EI) 252 [MH^+, 21%], 223 [60], 192 [84], 143 [24], 133 [100] and 78 [24].

4-Methyl hexa-2, 4-dienoic acid (61)

To a solution of triethyl-2-phosphonopropionate (10.6 g, 47.30 mmol) in hexane (90 mL) was added n-butyllithium (n-BuLi) (1.0 M solution in hexane, 47 mL) drop wise at room temperature. The resulting mixture was stirred at the same temperature for 30 min. *Trans*-2-methyl-2-butenal (4.2 mL, 49.9 mmol) was added drop wise to the mixture at 0 °C, and stirring was continued for 30 min at room temperature. After quenching the reaction by adding water (50 mL) the mixture was extracted with hexane (3×50mL). The organic extracts were combined, dried over anhydrous Na_2SO_4, filtered

and then concentrated *in vacuo*. The residue was dissolved in EtOH (45 mL) and 10% NaOH (35 mL) was added to the ethanolic solution. The mixture was heated at 50 °C for 16 h with stirring. After cooling the reaction mixture was washed with hexane (3 × 50 mL). The aqueous layer was made acidic (pH 1) by adding 2 M HCl solution to precipitate dieneoic acid **61**. After filtration and drying *in vacuo* the compound obtained was used directly for further feeding studies. λ_{max} (MeOH) = 265 nm; δ_H (500 MHz, $CDCl_3$), 1.80 (3H, t, *J* 1.1 CH_3-7), 1.84 (3H, d, *J* 7.0 CH_3-6), 5.81 (1H, d, *J* 15.6, H-2), 6.07 (1H, q, *J* 7.0 H-5) 7.42 (1H, d, *J* 15.6, H-3); δ_C (125 MHz) 12.3 (C-7), 13.8 (C-6), 121.3 (C-2), 125.3 (C-5), 135.7 (C-4), 148.5 (C-3), 171.2 (C-1); *m/z* (ESI) 149 [MNa$^+$, 77%], 127 [40%], 109 [20%].

Figures and Schemes

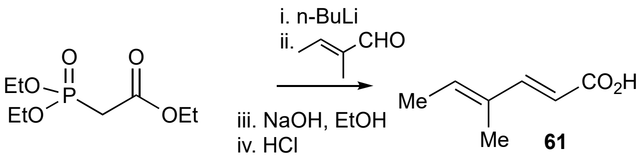

Scheme S1. Synthesis of 4-methylhexa-2,4-dienoic acid (**61**).

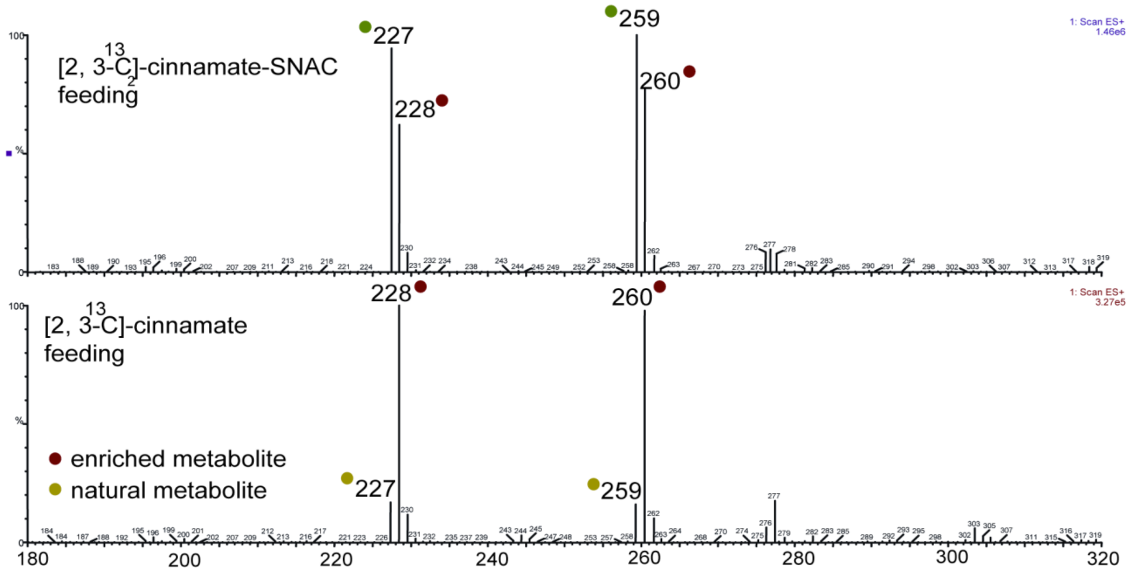

Figure S1. LCMS analysis shows that the rate of cinnamic acid incorporation is higher than its SNAC thiolester in culture of *B. lutea* F23523.

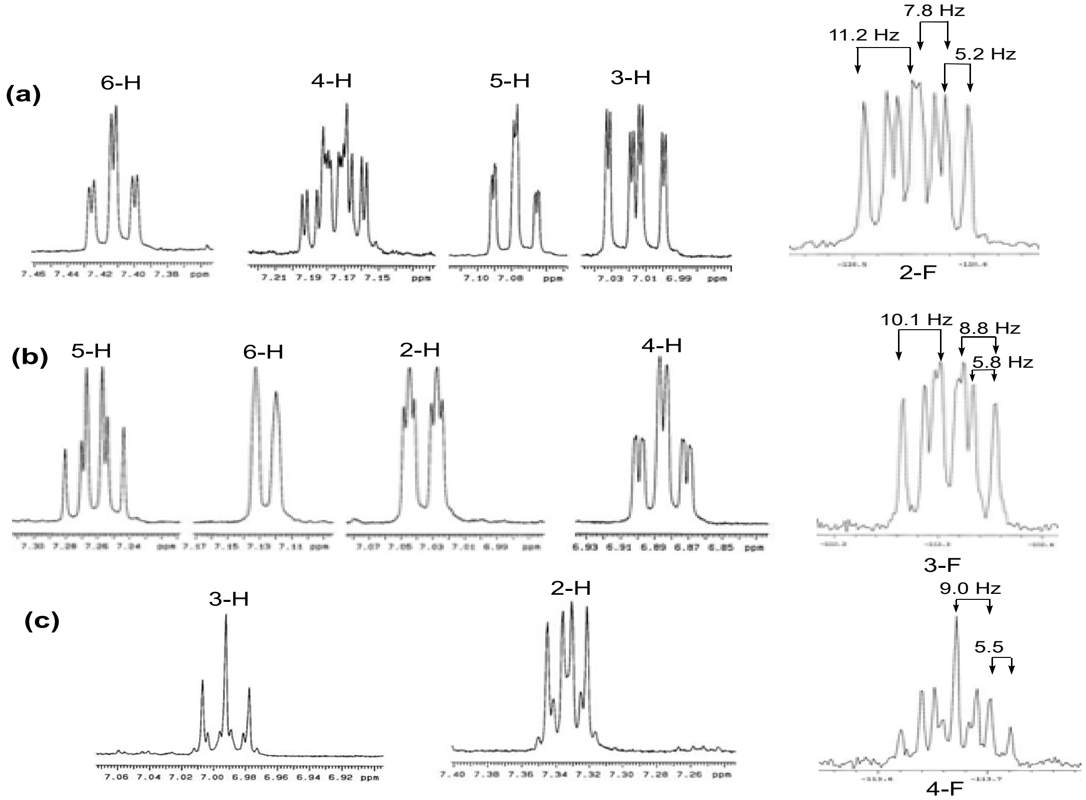

Figure S2. Aromatic regions of the ^{1}H and ^{19}FNMR spectra of (a) 2-fluoro -, (b) 3-fluoro-, and (c) 4-fluorostrobilurin A.

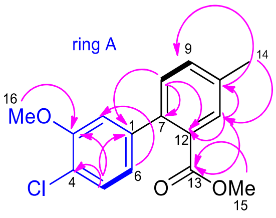

39

Figure S3. Selected COSY (bold) and HMBC correlations (plain arrows) in pseudostrobilurin B **39.**

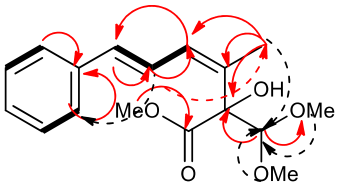

strobilurin Y **41**

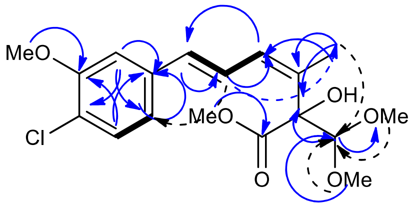

strobilurin Z **42**

Figure S4. Selected COSY and HMBC correlations in strobilurins Y **41** and Z **42.**

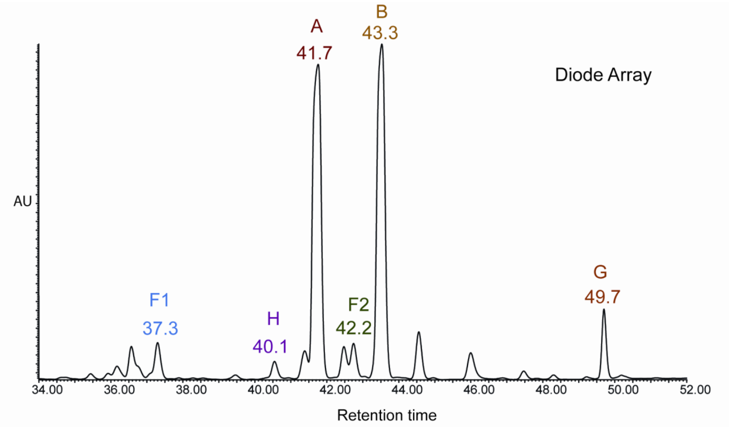

Figure S5. LCMS chromatogram showing strobilurin production after optimization of fermentation conditions.

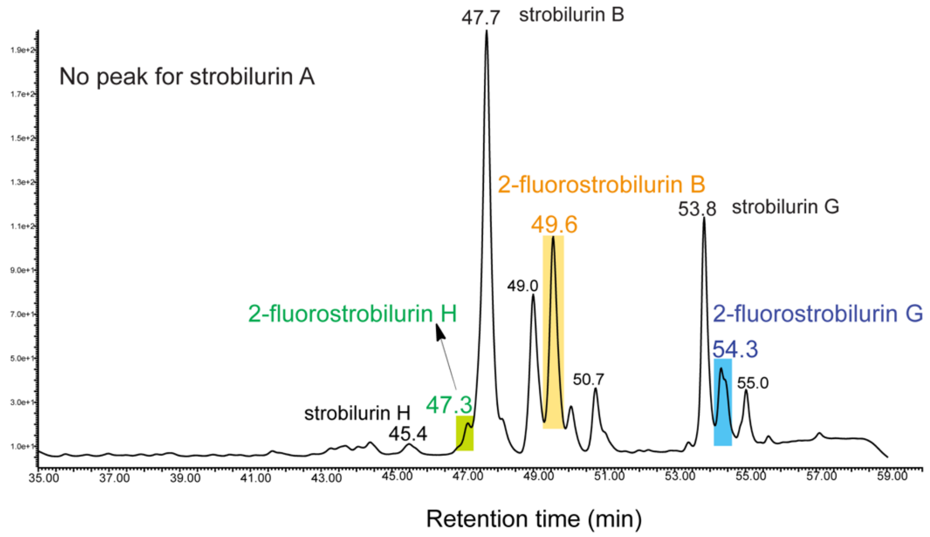

Figure S6 LCMS analysis of crude extract of *B. lutea* culture strain F23523 fed with 2fluorocinnamate.

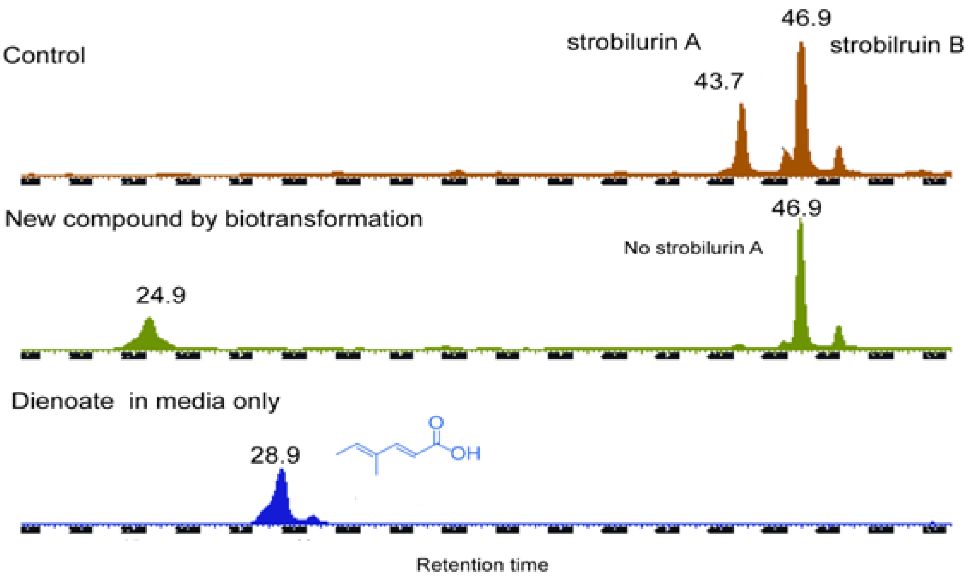

Figure S7. Biotransformation of dienoic acid **62** (Rt = 28.9 min) by *B. lutea* to diol **63** (RT = 24.9 min). 100 % bioconversion observed. Strobilurin A **2** (Rt = 43.7) production completely inhibited while strobilurin B **3** is produced as indicated at RT 46.9.

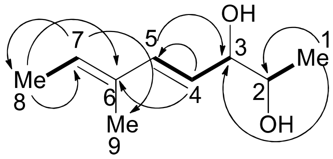

62

Figure S8. Key COSY (bold lines) and HMBC (plain arrows) correlations in diol **62**.

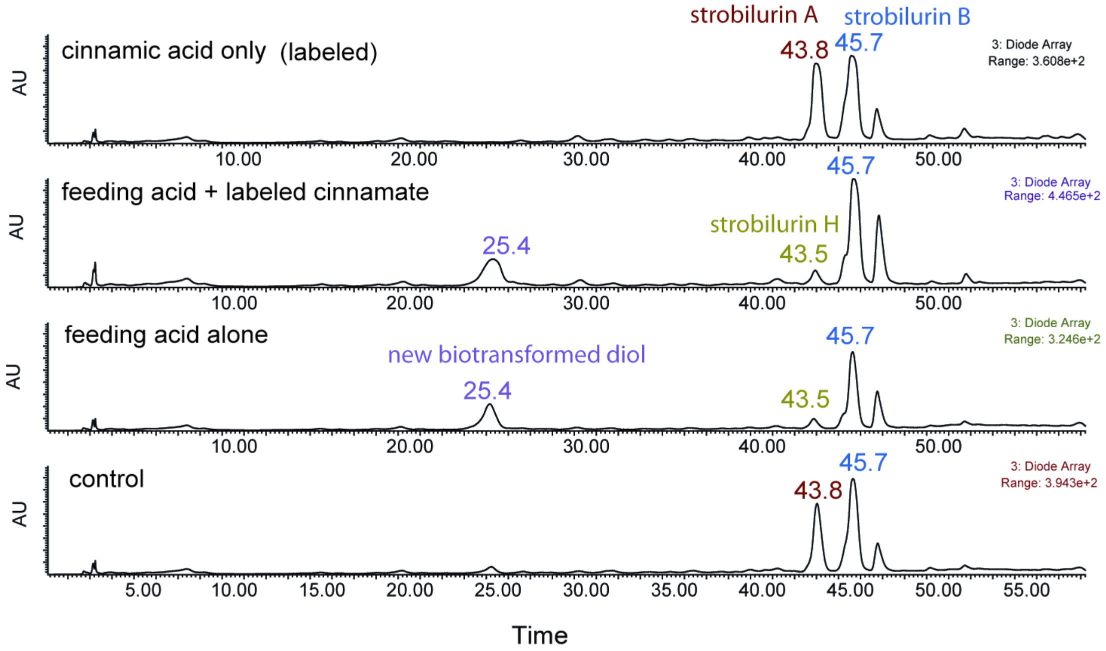

Figure S9. Feeding of both [2,3-$^{13}C_2$]-cinnamate + dienoic acid **61** to the same *B. lutea* culture. The labelled cinnamic acid is incorporated in strobilurin B **3**. Cinnamate feeding to the culture does not restore strobilurin A **2** production.

NMR Spectra

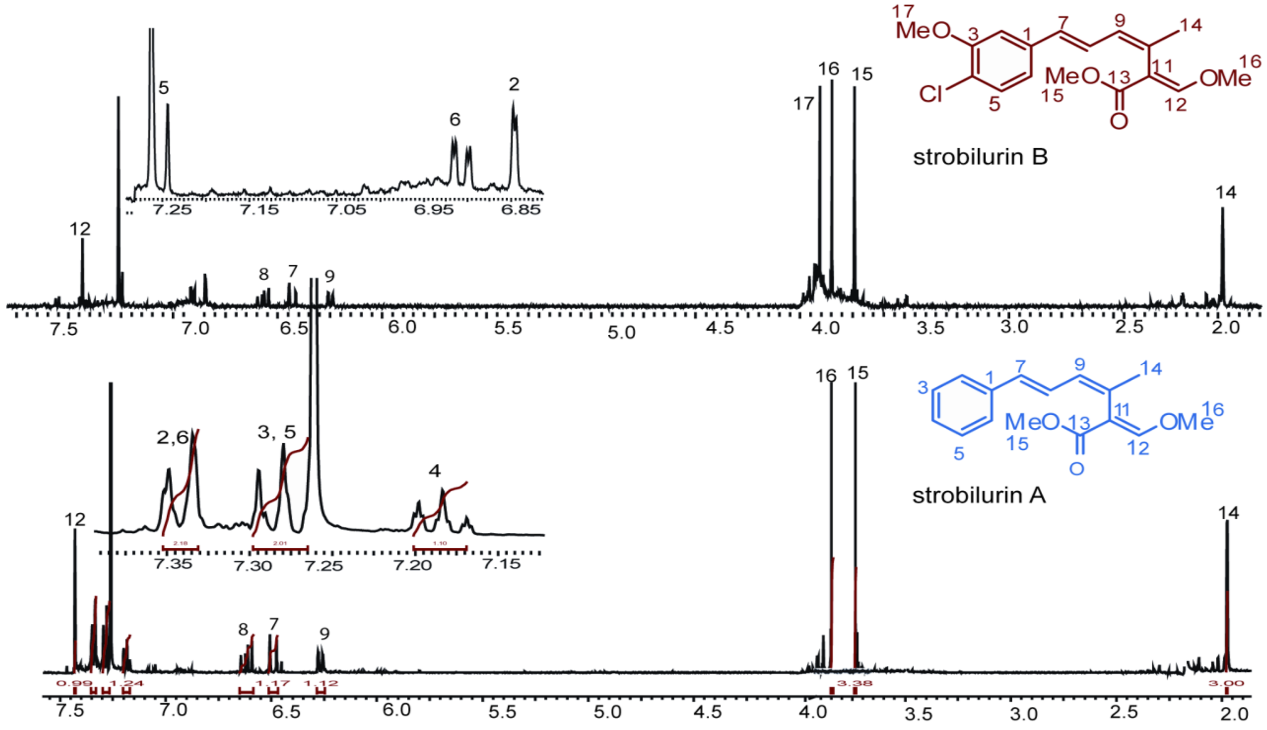

Figure S.10 ^{1}H NMR in $CDCl_3$ (500 MHz) spectra of strobilurin A **2** (below) and B 3 (above).

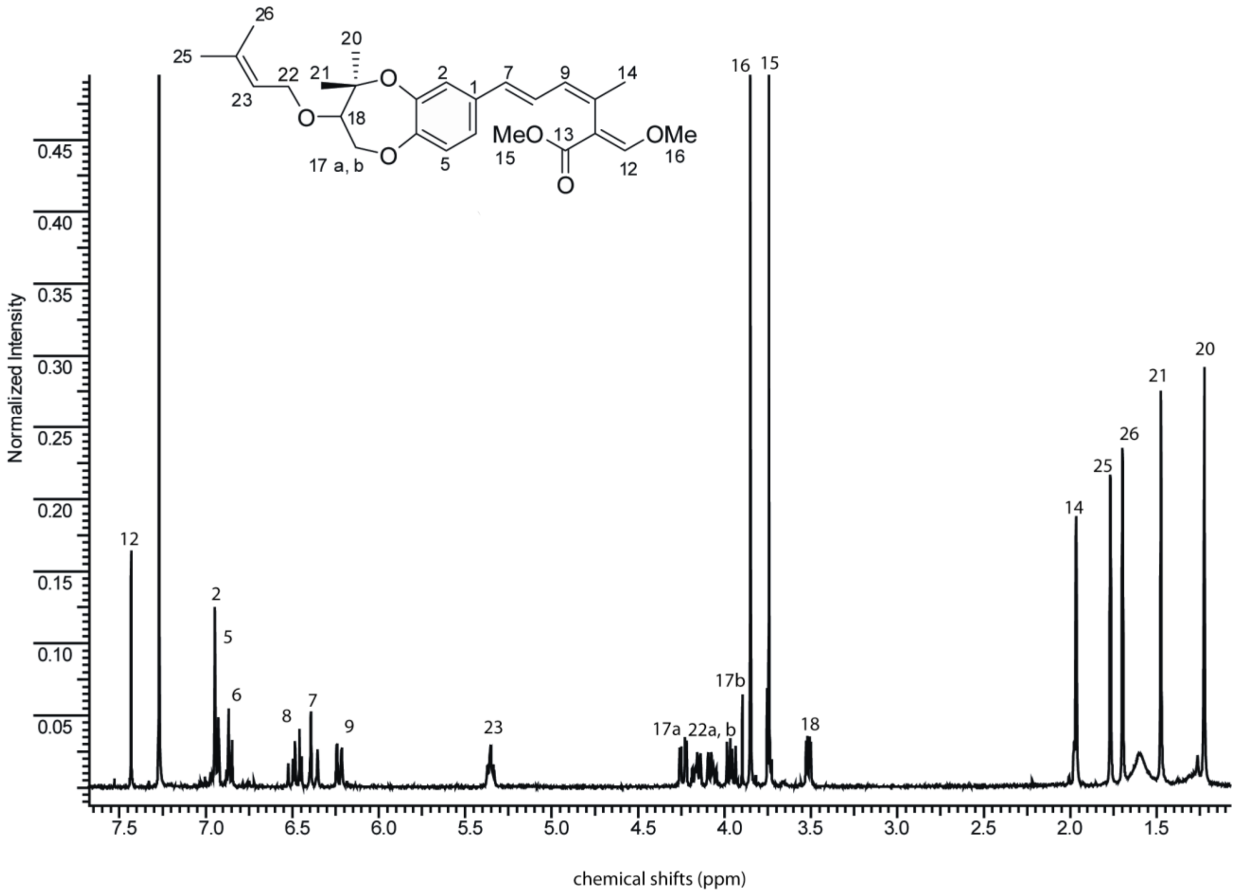

Figure S11 ^{1}H NMR spectrum ($CDCl_3$) of strobilurin G **7** isolated from *B. lutea*.

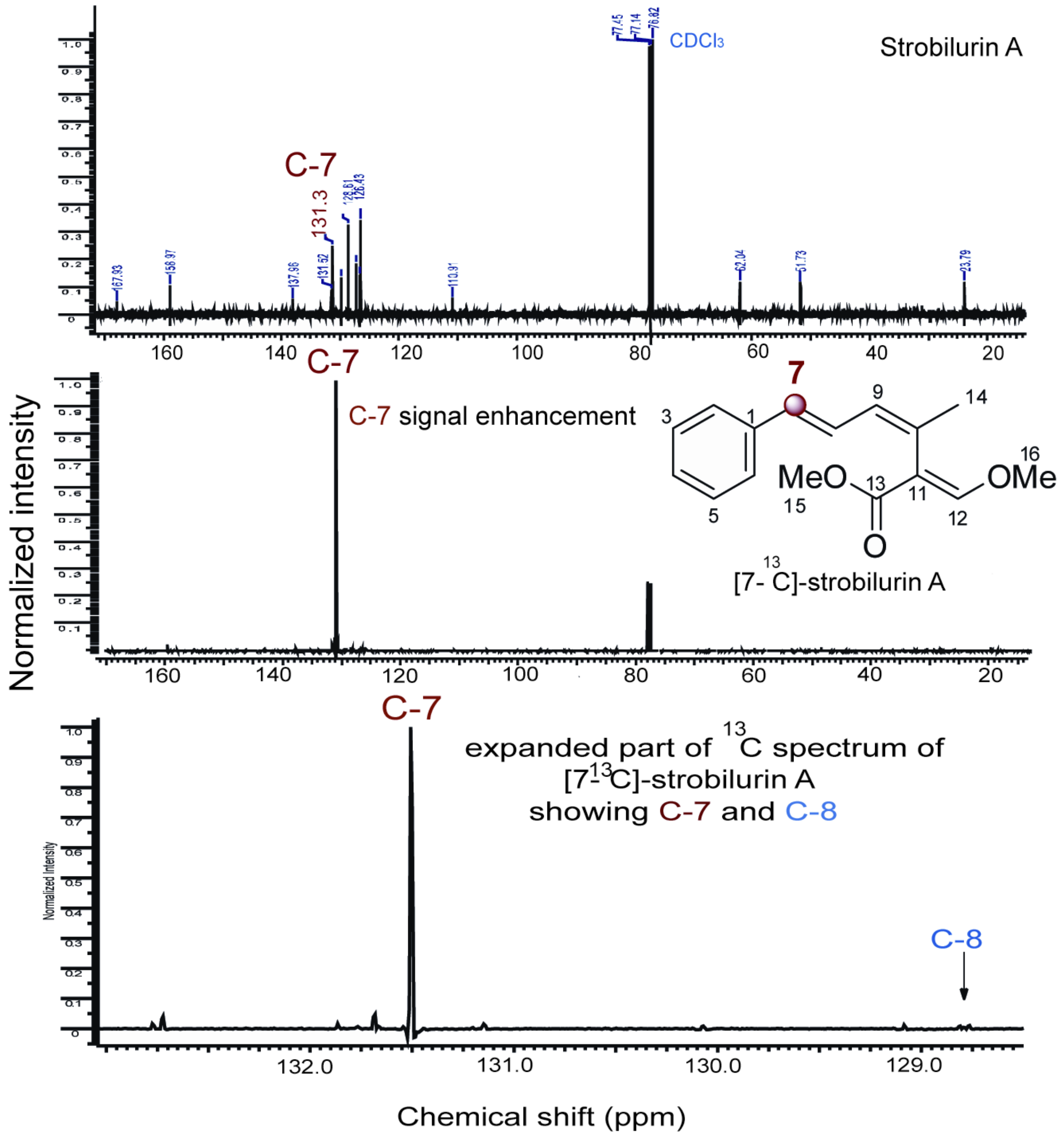

Figure S12 Comparison of ^{13}C-NMR spectrum of [7-^{13}C]-strobilurin A to that of strobilurin A.

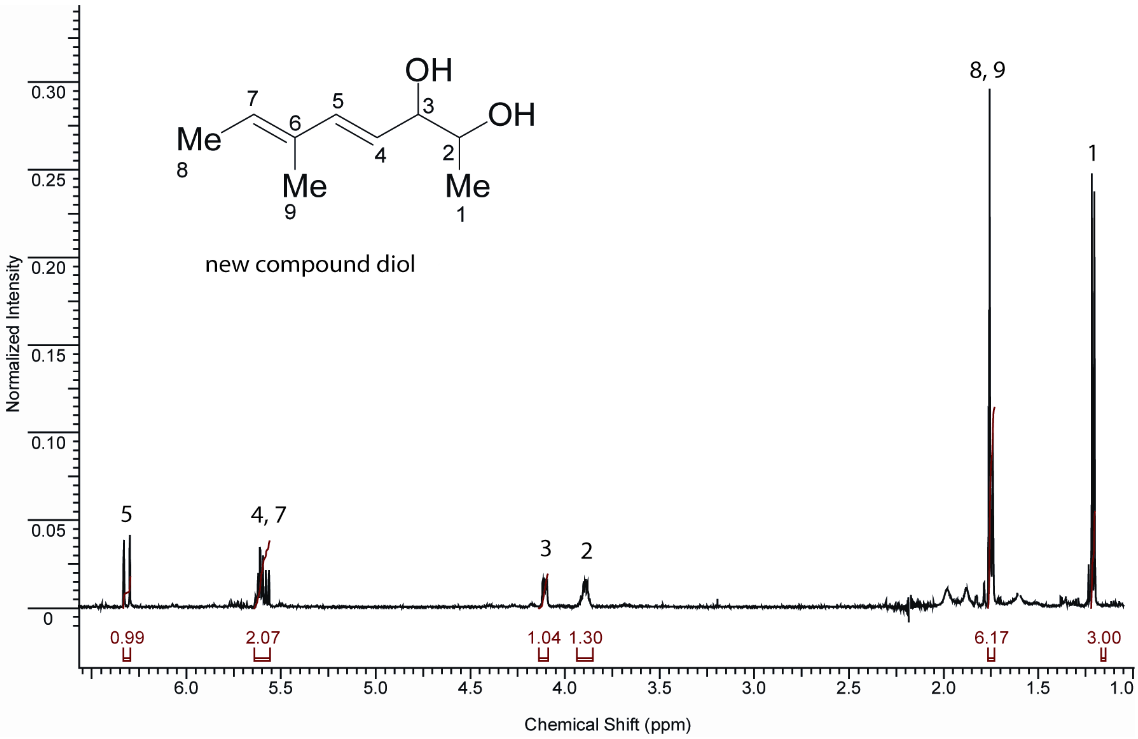

Figure S13. ^{1}H NMR spectrum of the diol 62 from biotransformation of acid **61**.

(a)

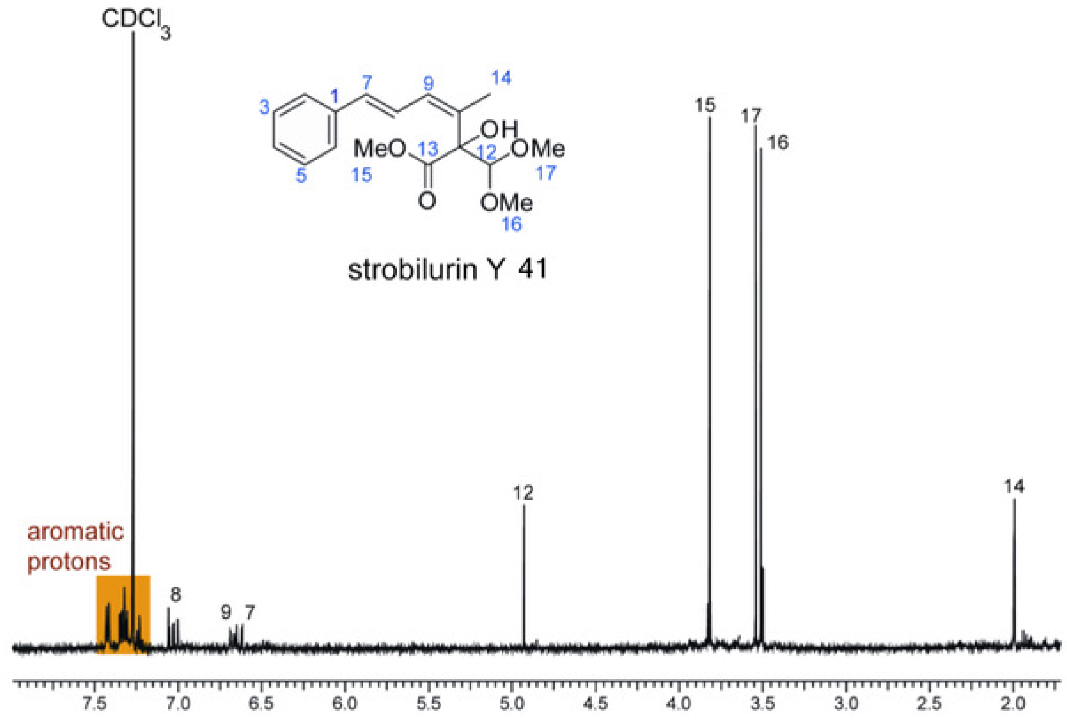

(b)

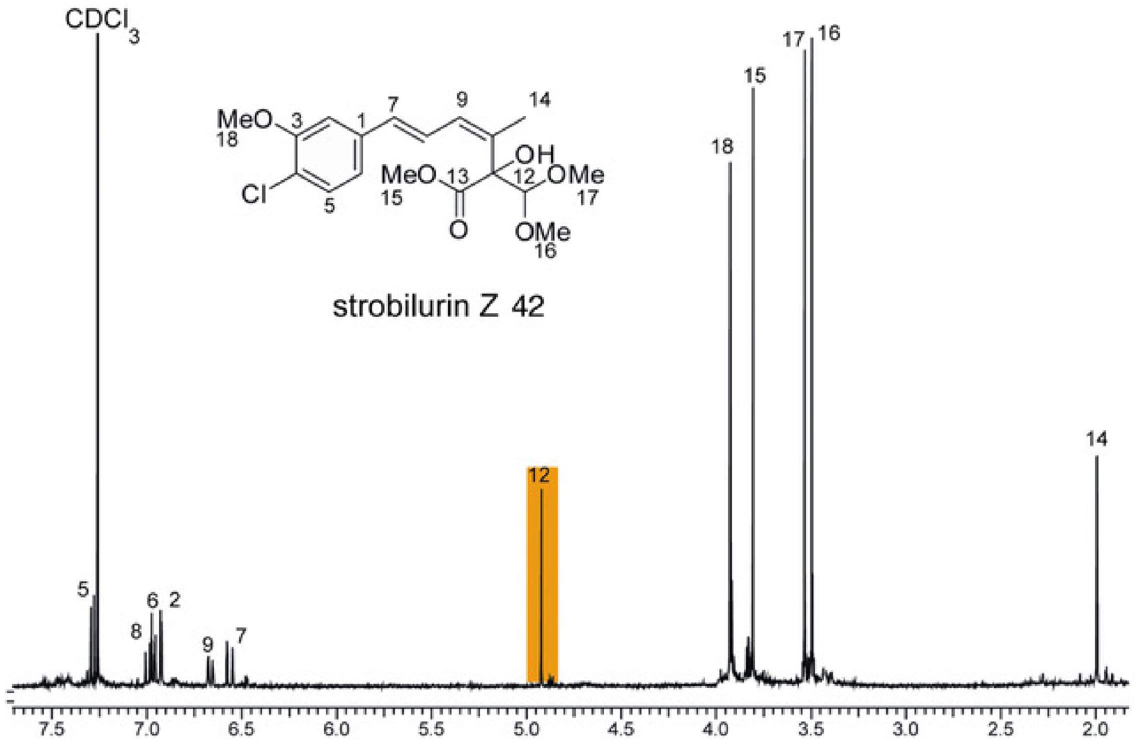

Figure S14. ^{1}H NMR spectra of (a) strobilurin Y **41** and (b) strobilurin Z **42**.

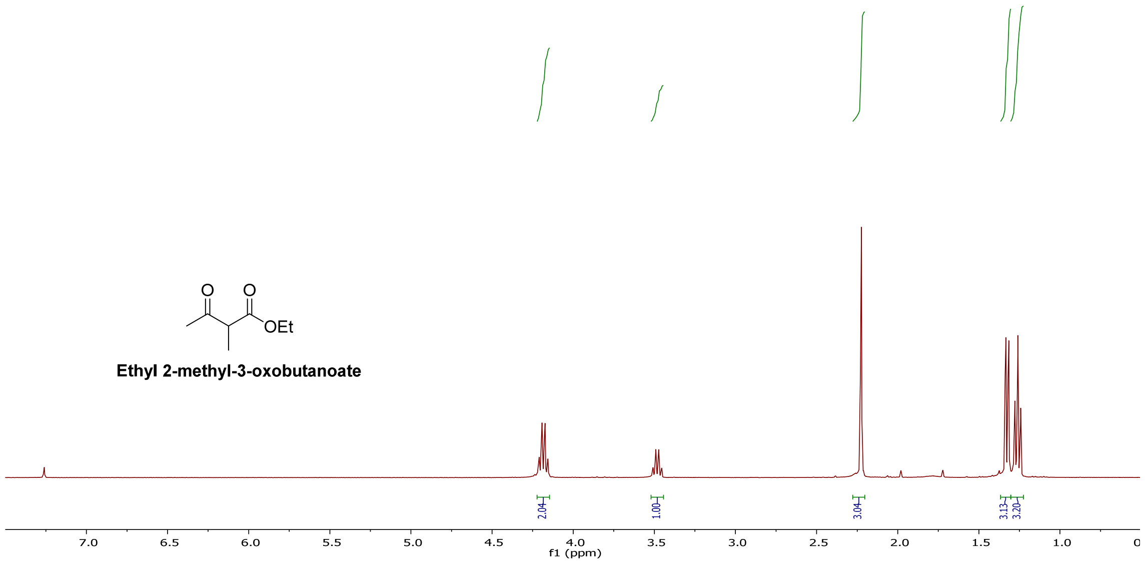

Figure S15. ^{1}H NMR spectrum of ethyl 2-methyl-3-oxobutanoate.

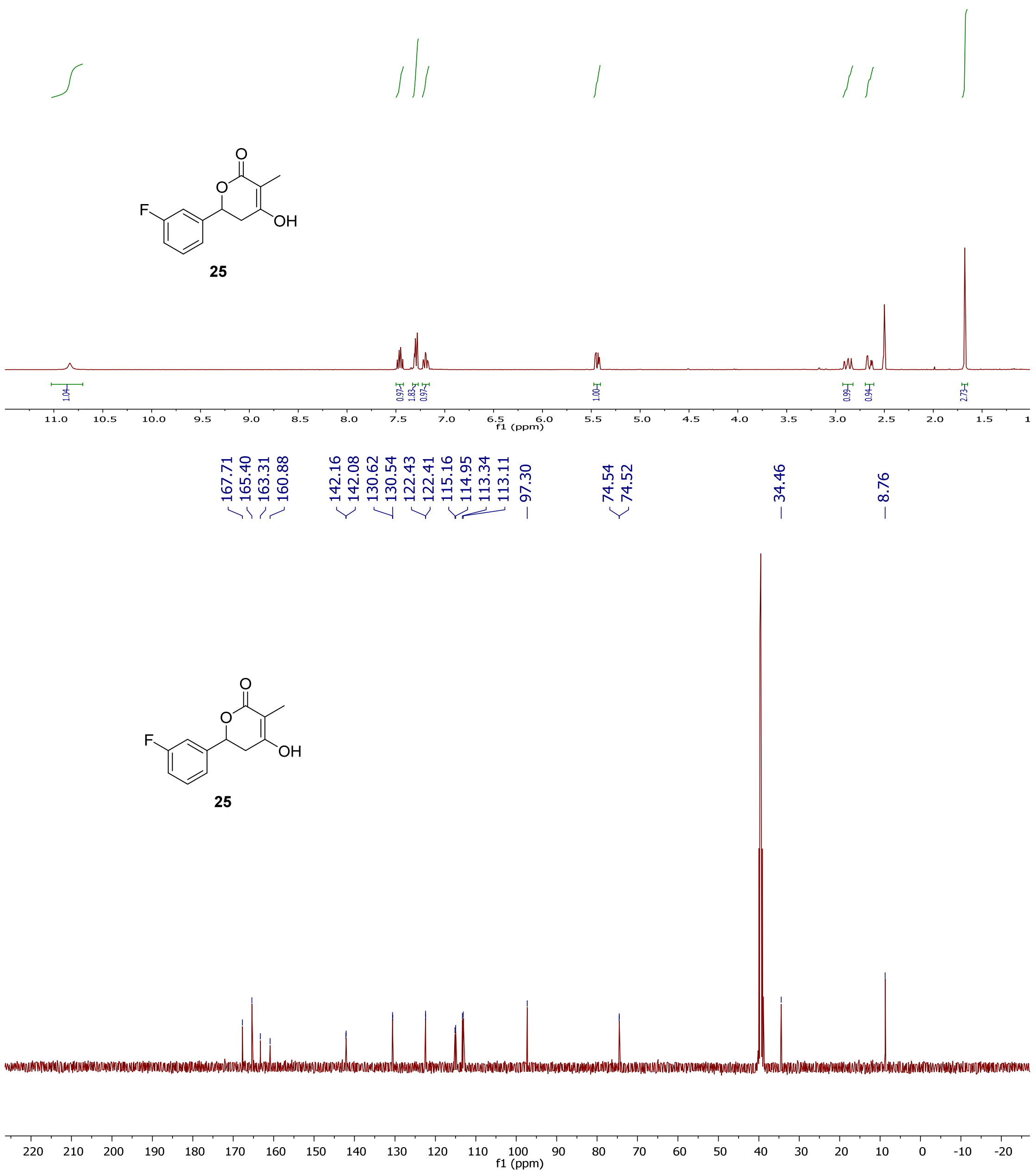

Figure S16. ^{1}H and ^{13}C NMR spectra of lactone **25**.

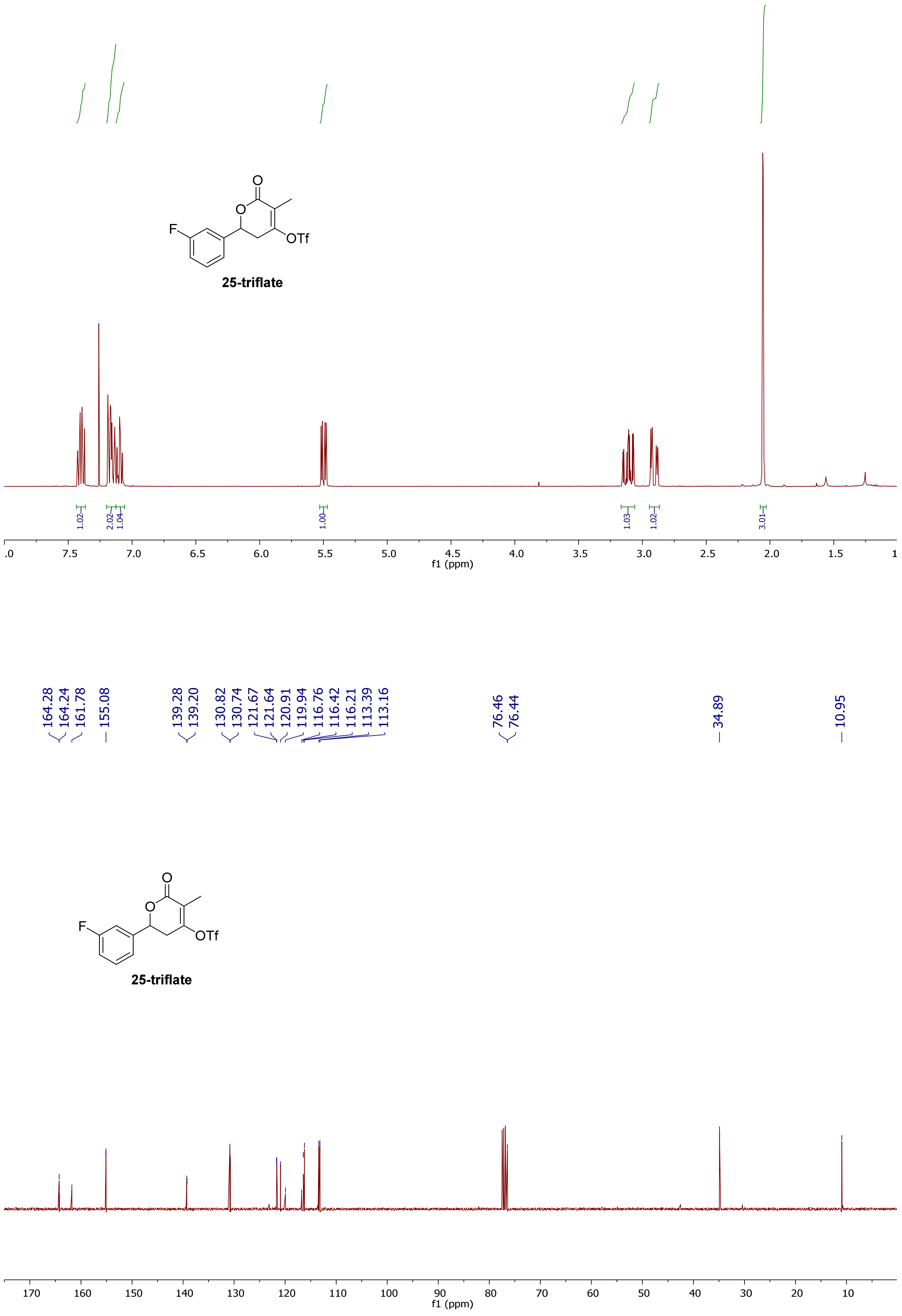

Figure S17. ^{1}H and ^{13}C NMR spectra of lactone **25** triflate.

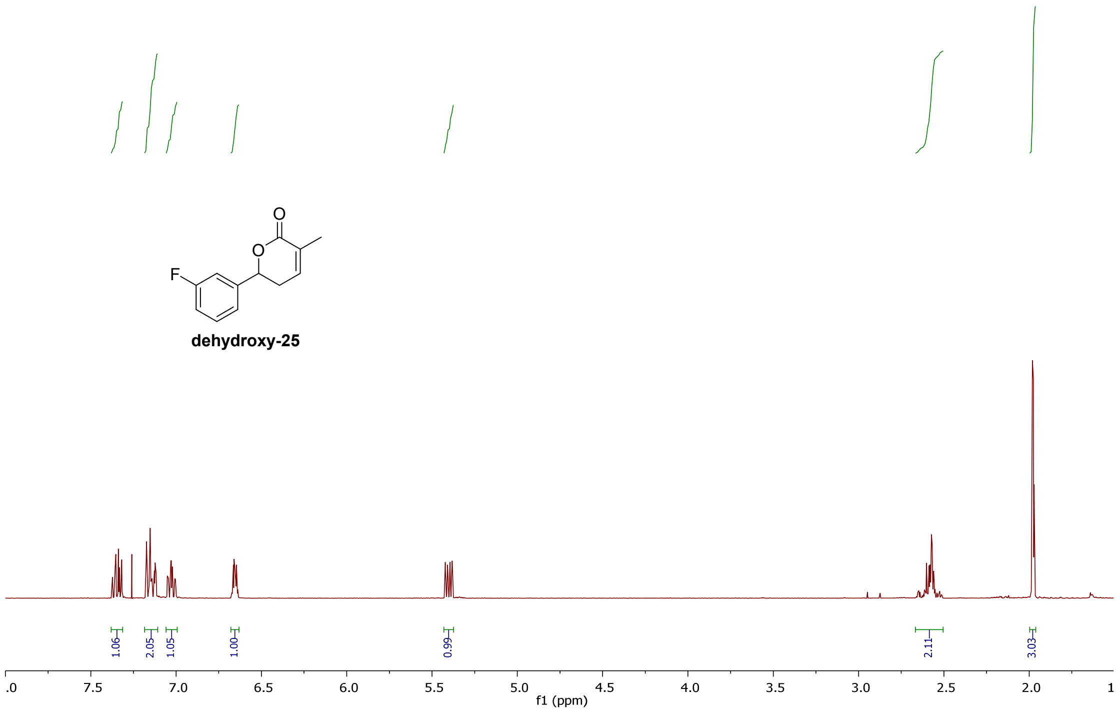

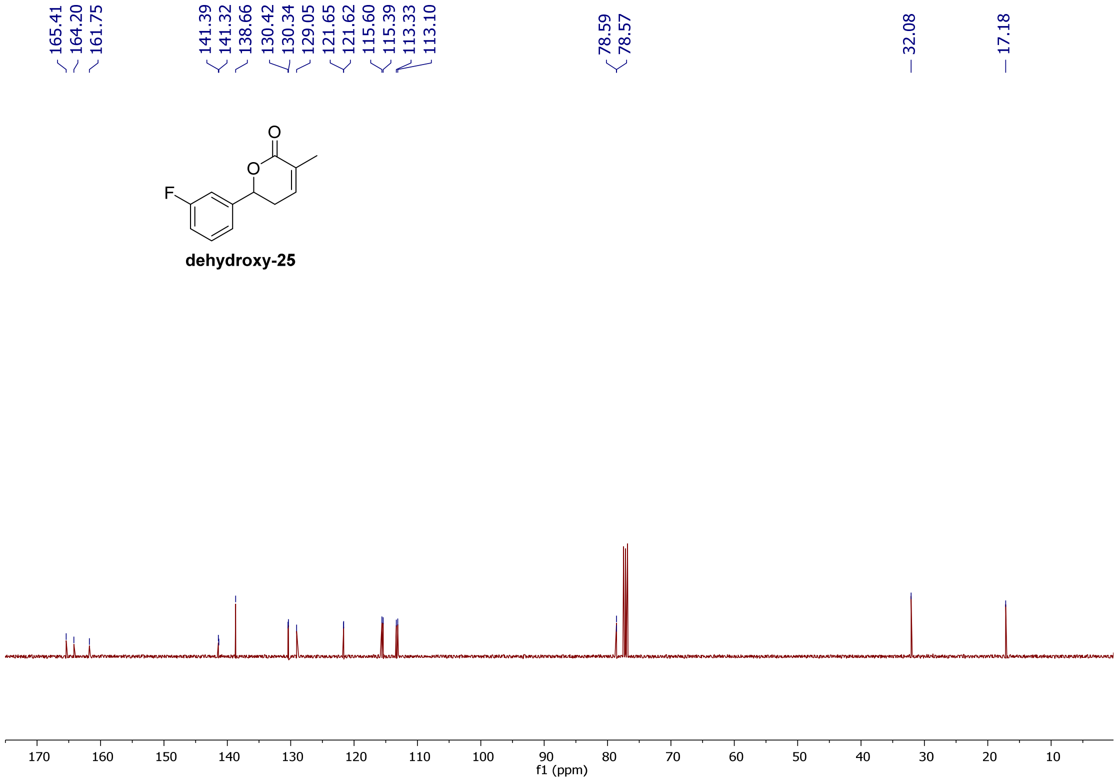

Figure S18. ^{1}H and ^{13}C NMR spectra of dehydroxy-lactone **25**.

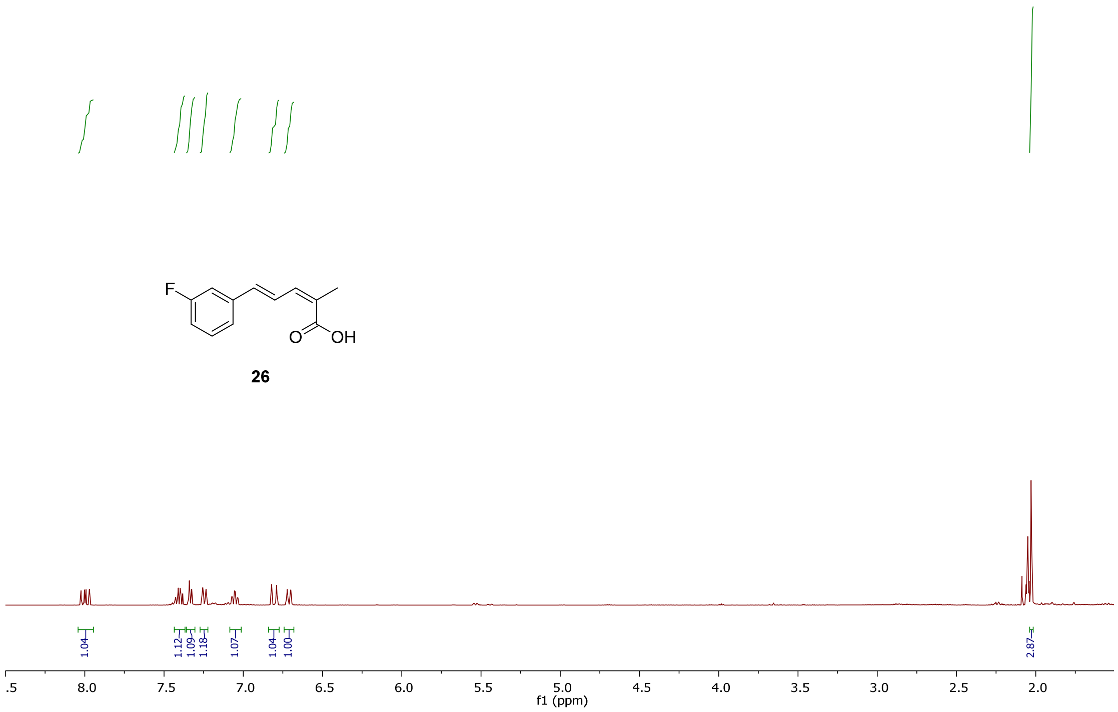

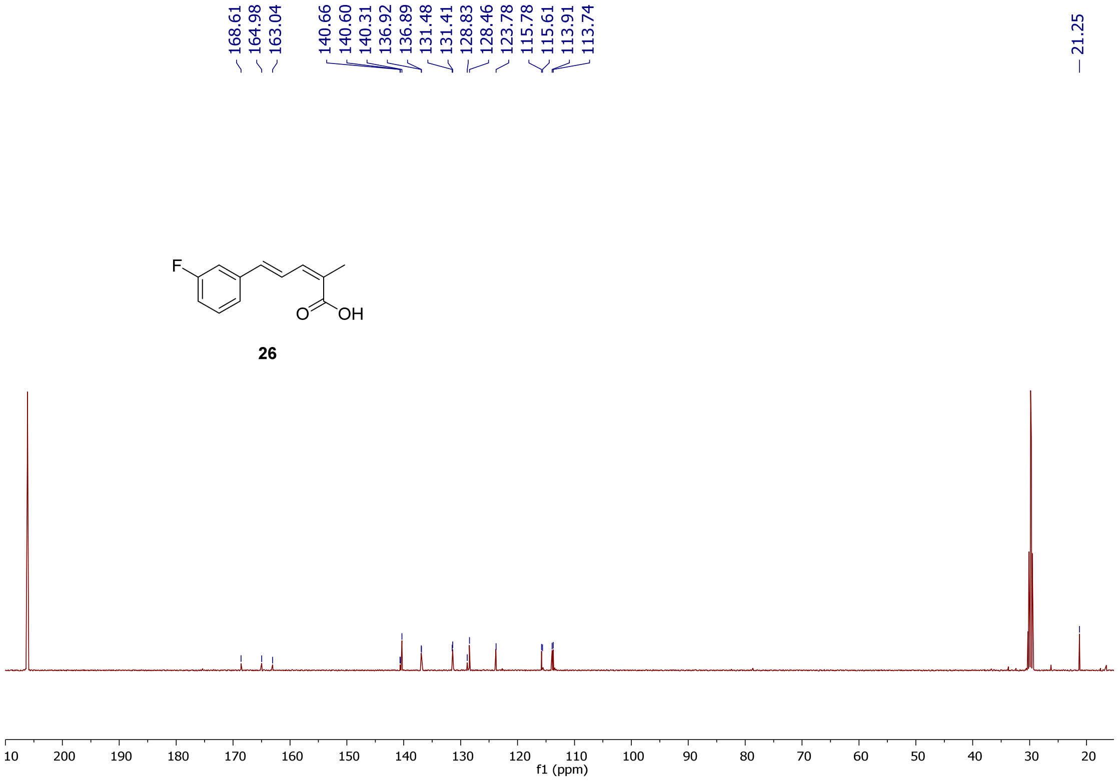

Figure S19. ^{1}H and ^{13}C NMR spectra of (2*Z*,4*E*)-5-(3'-Fluorophenyl)-2-methylpenta-2,4-dienoic acid **26.**

.

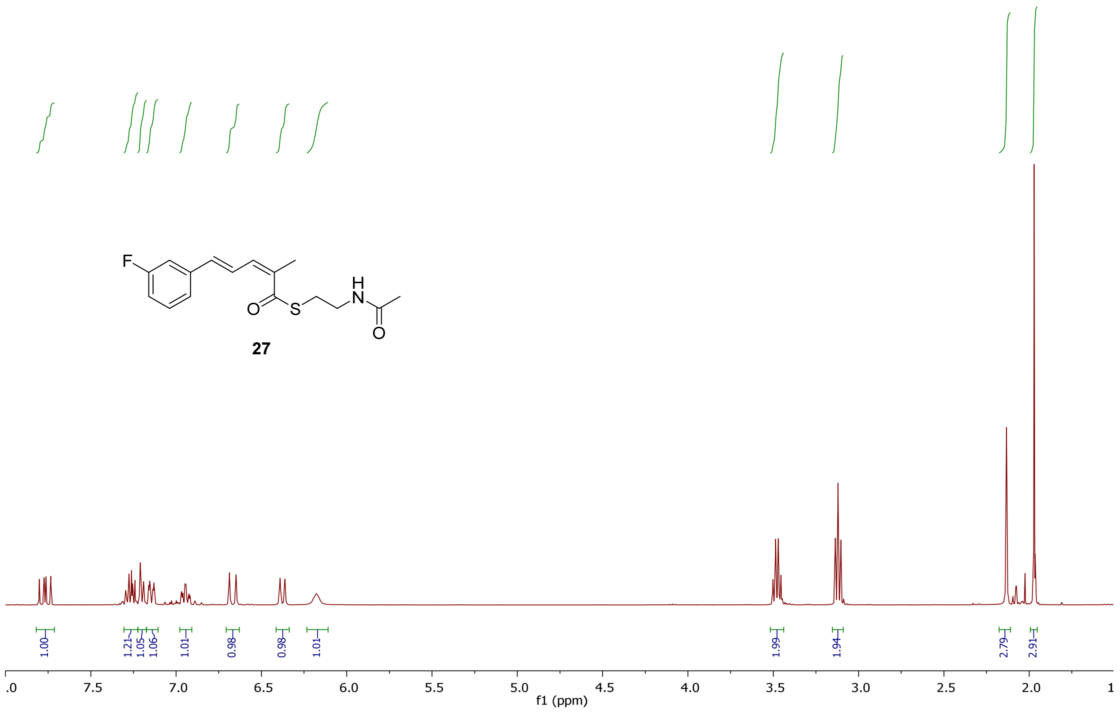

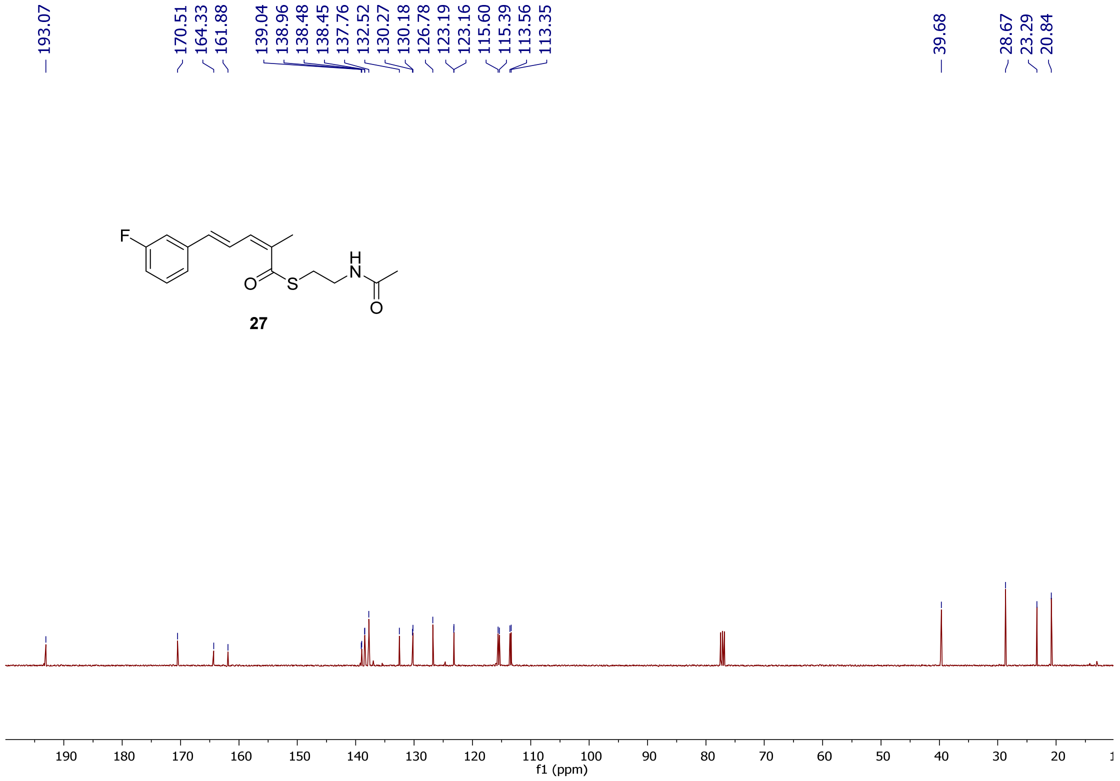

Figure S20. ^{1}H and ^{13}C NMR spectra of *S*-(2-Acetamidoethyl) (2*Z*,4*E*)-5-(3-fluorophenyl)-2-methylpenta-2,4-dienethioate **27**.

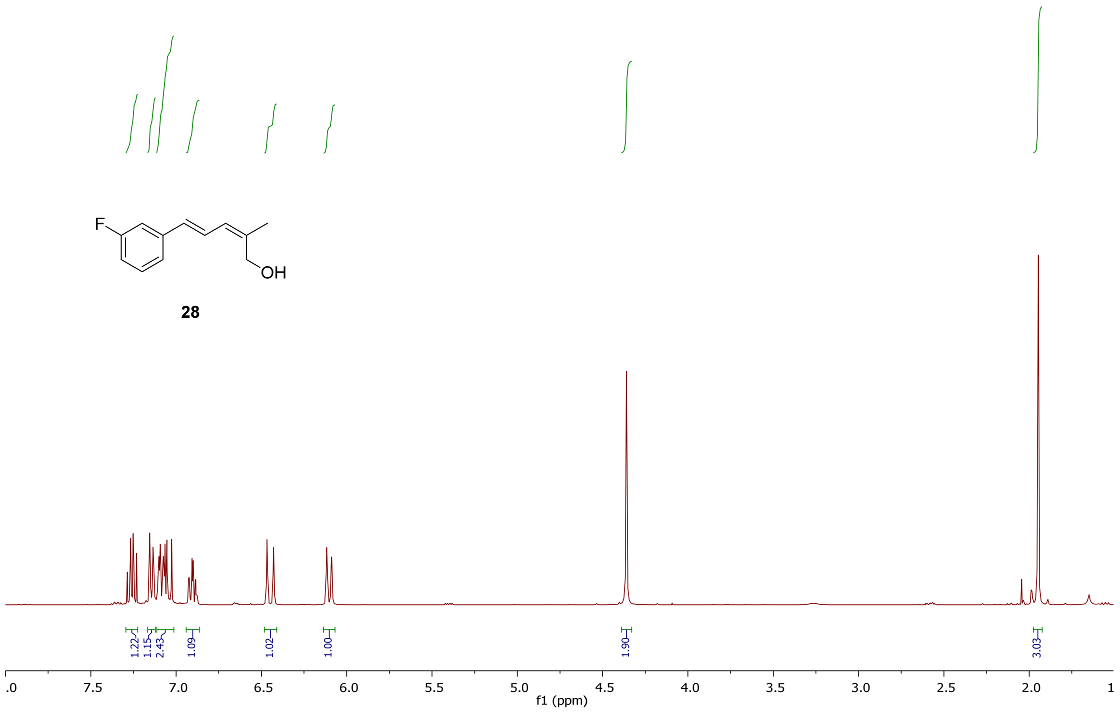

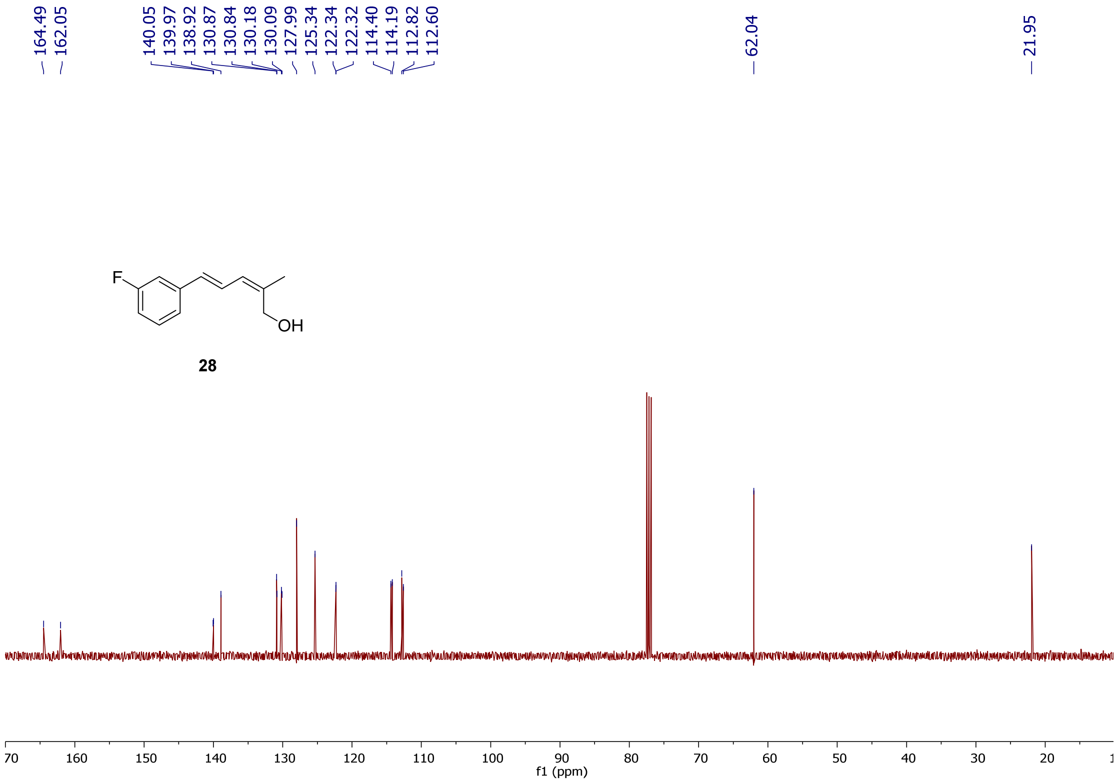

Figure S21. ^{1}H and ^{13}C NMR spectra of (2*Z*,4*E*)-5-(3-Fluorophenyl)-2-methylpenta-2,4-dien-1-ol **28**.

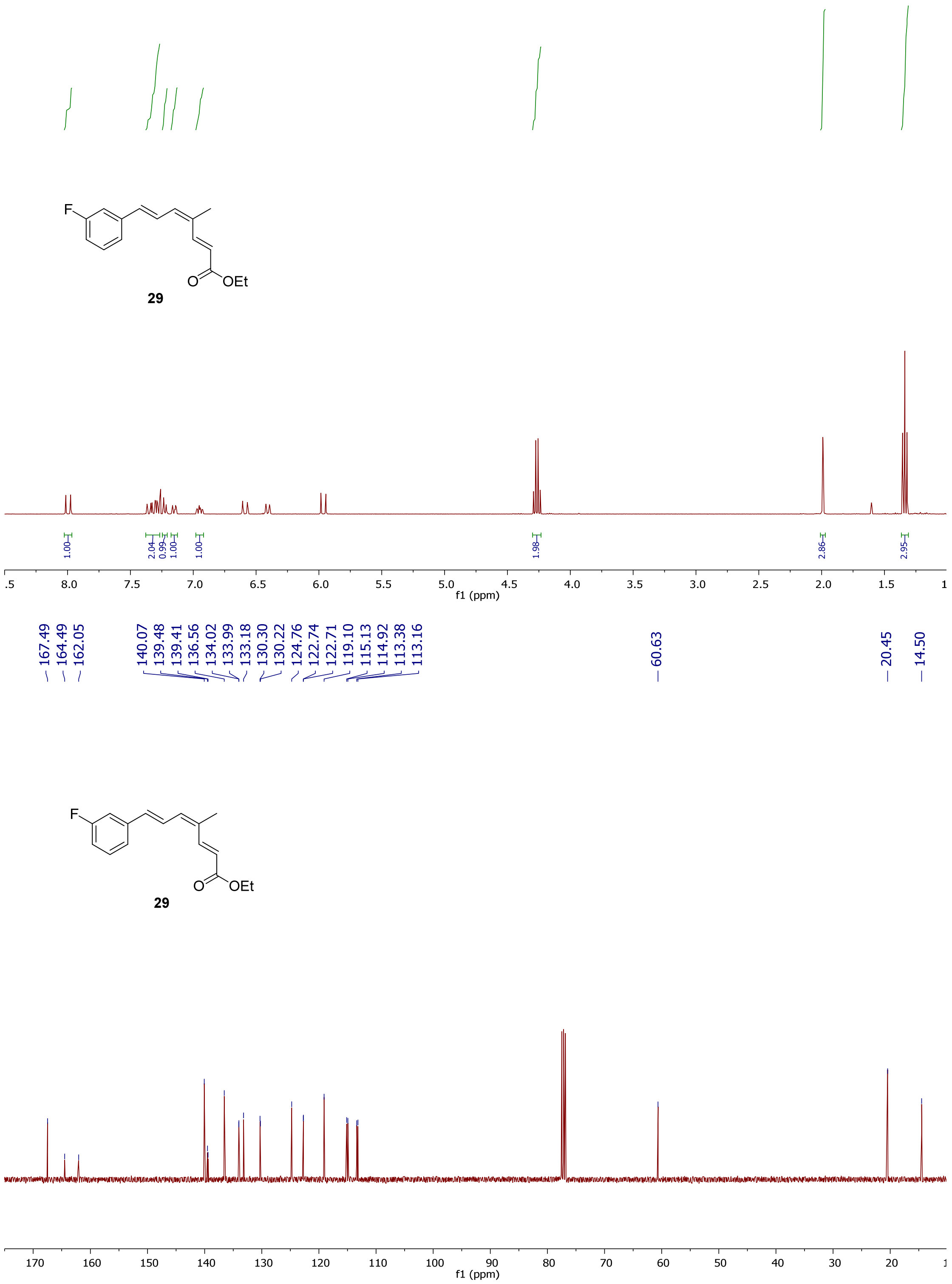

Figure S22. ^{1}H and ^{13}C NMR spectra of ethyl (2*E*,4*Z*,6*E*)-7-(3-fluorophenyl)-4-methylhepta-2,4,6-trienoate **29**.

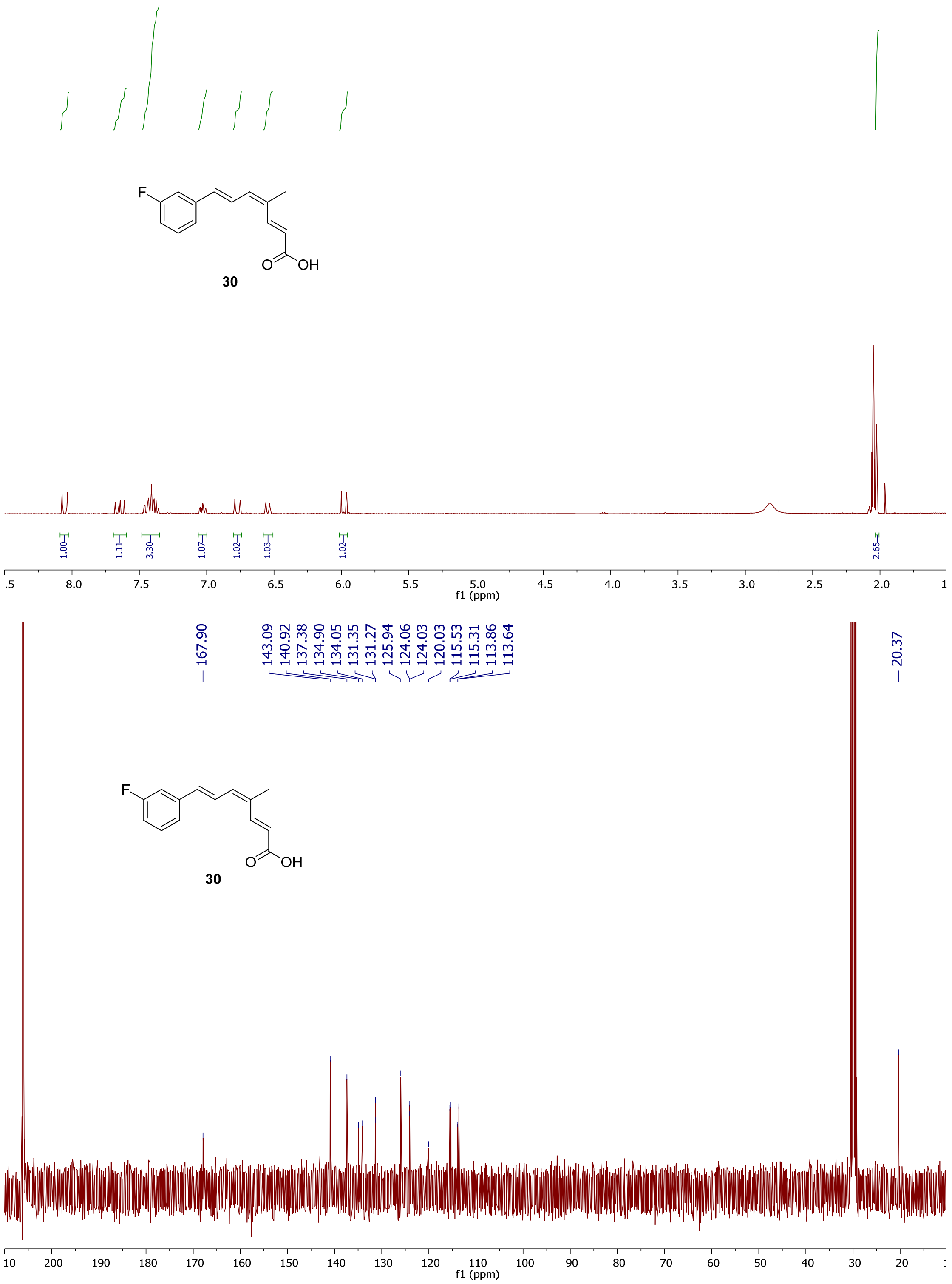

Figure S23. ^{1}H and ^{13}C NMR spectra of (2*E*,4*Z*,6*E*)-7-(3-Fluorophenyl)-4-methylhepta-2,4,6-trienoic acid **30.**

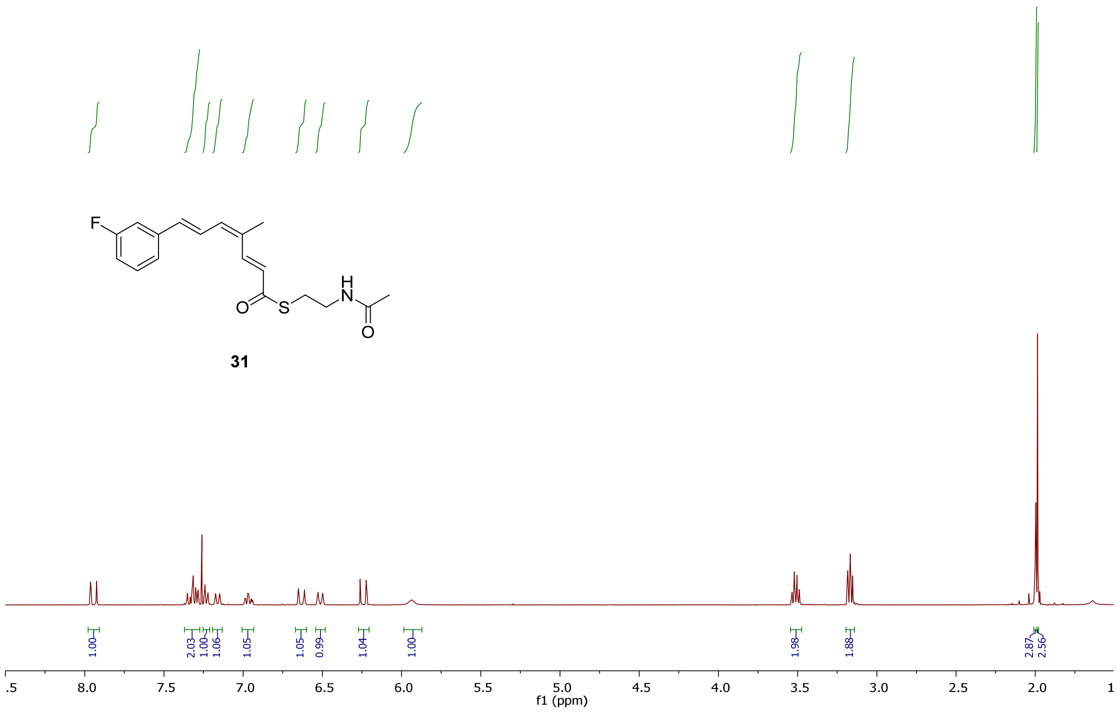

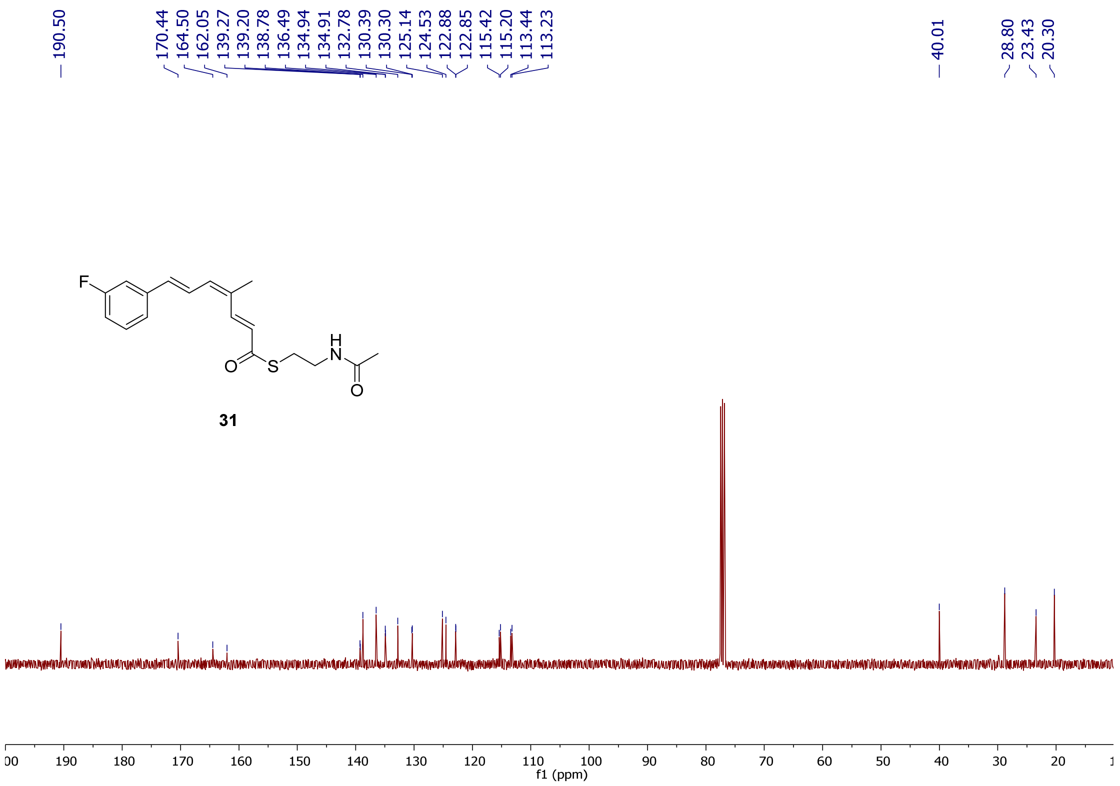

Figure S24. ^{1}H and ^{13}C NMR spectra of *S*-(2-Acetamidoethyl) (2*E*,4*Z*,6*E*)-7-(3-fluorophenyl)-4-methylhepta-2,4,6-trienethioate **31**.

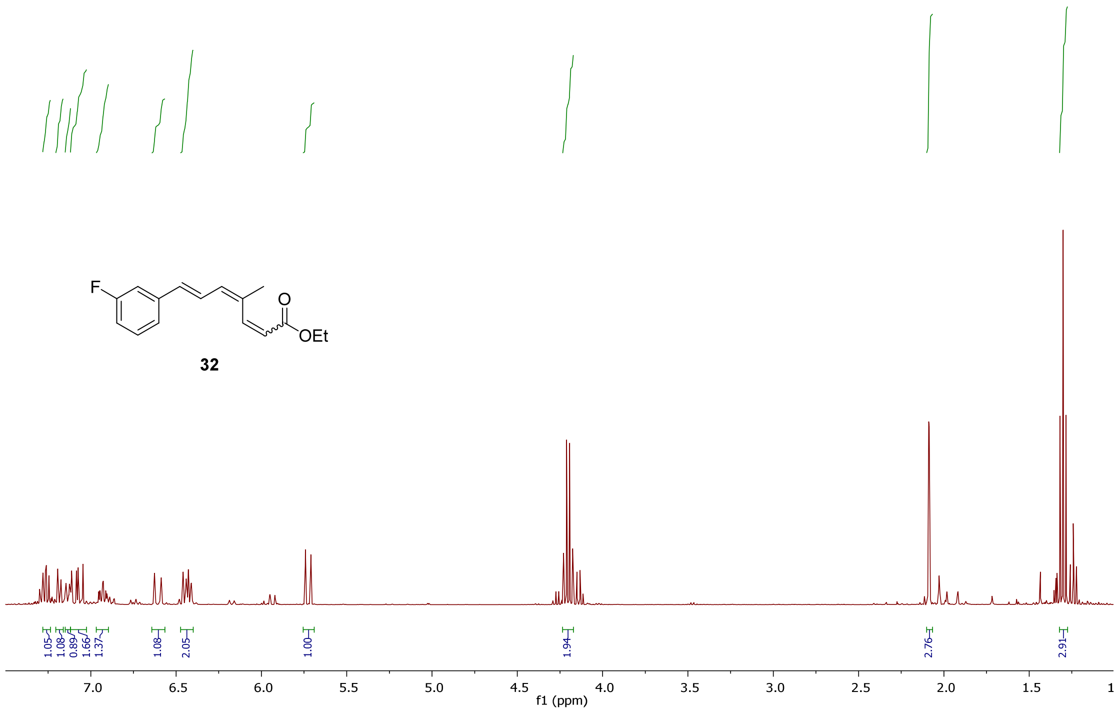

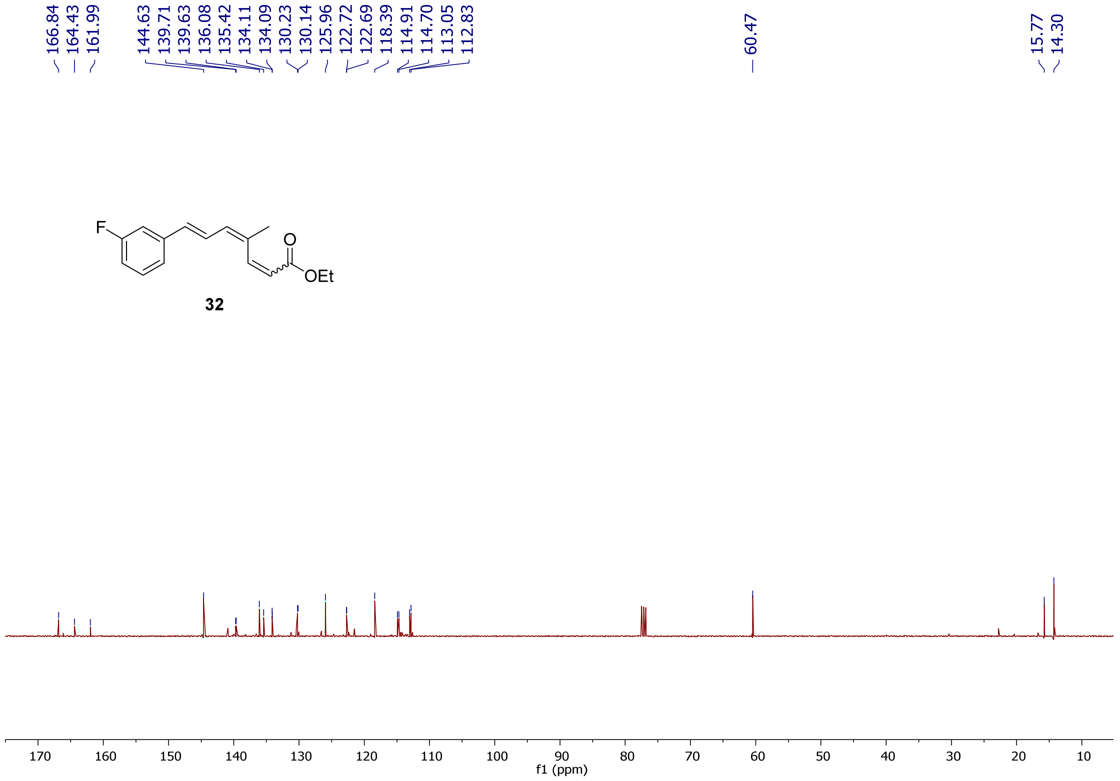

Figure S25. ^{1}H and ^{13}C NMR spectra of ethyl (2*Z*,4*Z*,6*E*)-7-(3-fluorophenyl)-4-methylhepta-2,4,6-trienoate **32**.

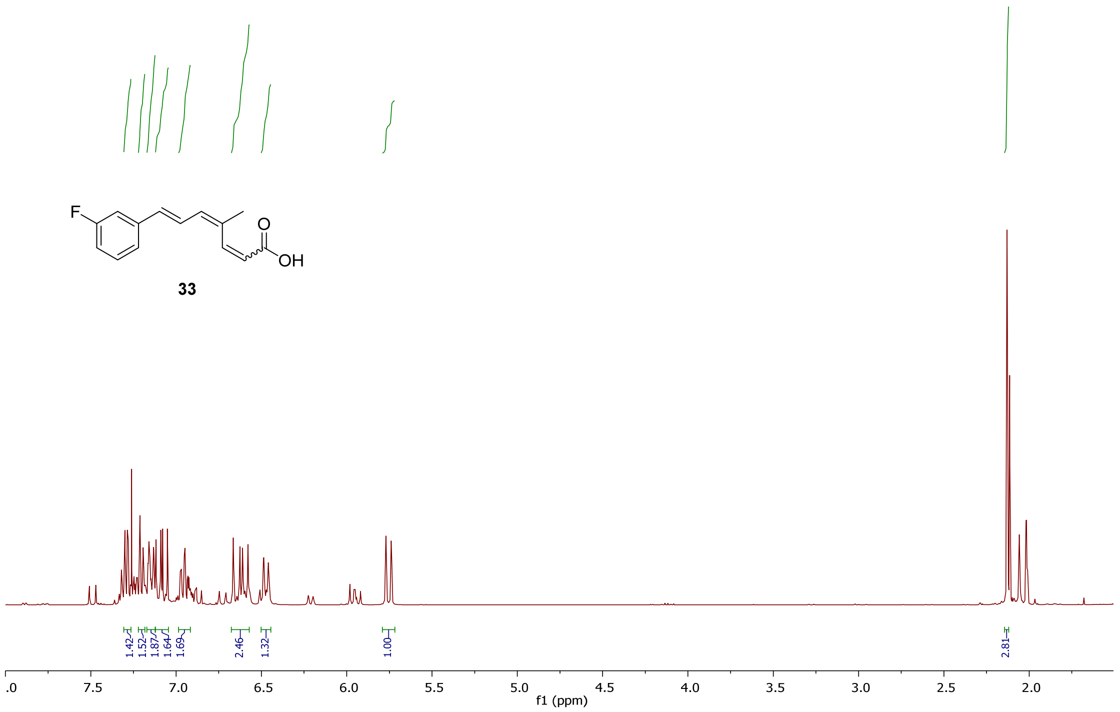

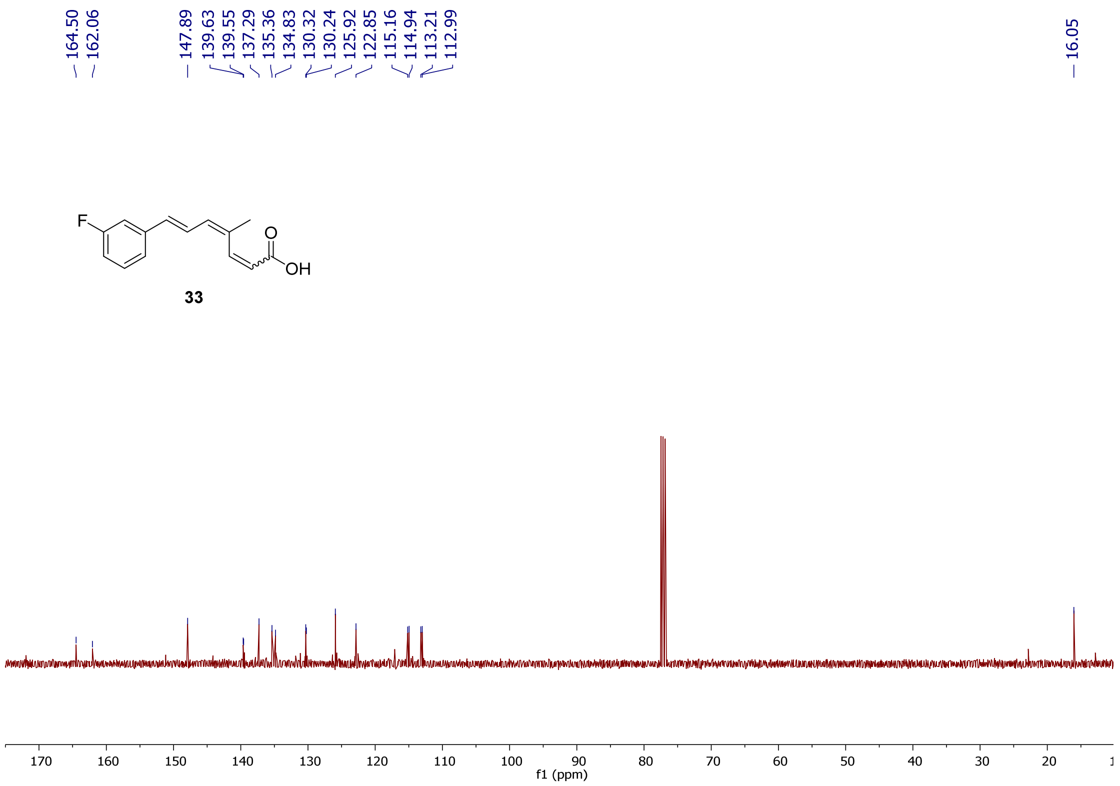

Figure S26. ^{1}H and ^{13}C NMR spectra of (2*Z*,4*Z*,6*E*)-7-(3-Fluorophenyl)-4-methylhepta-2,4,6-trienoic acid **33**.

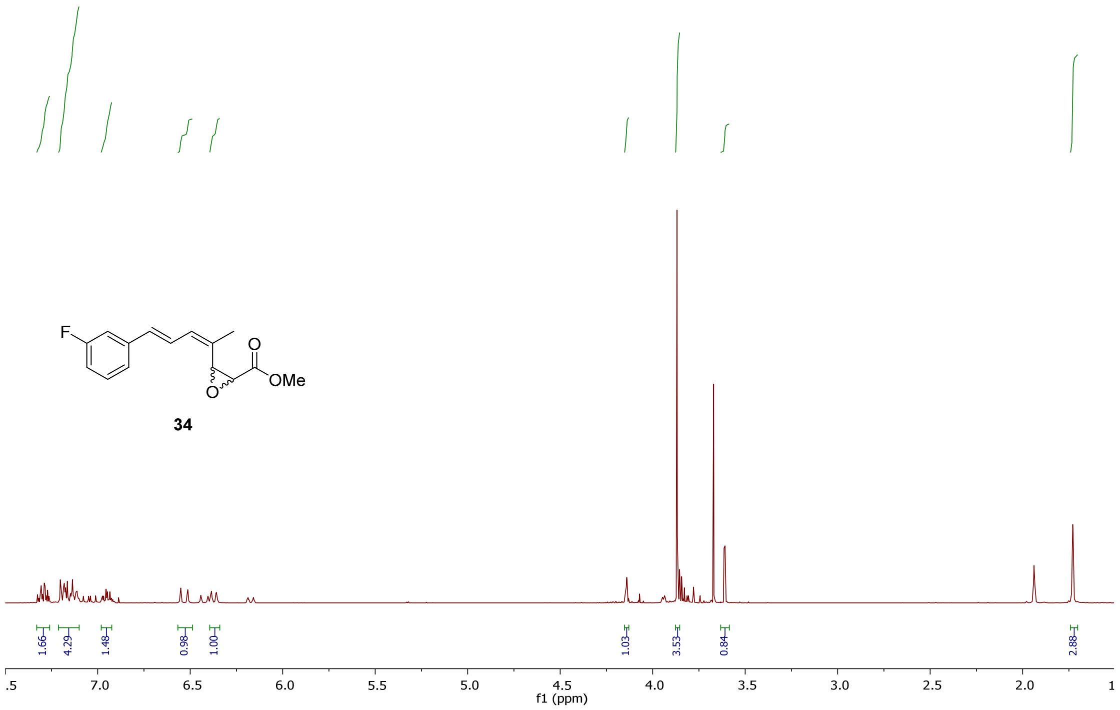

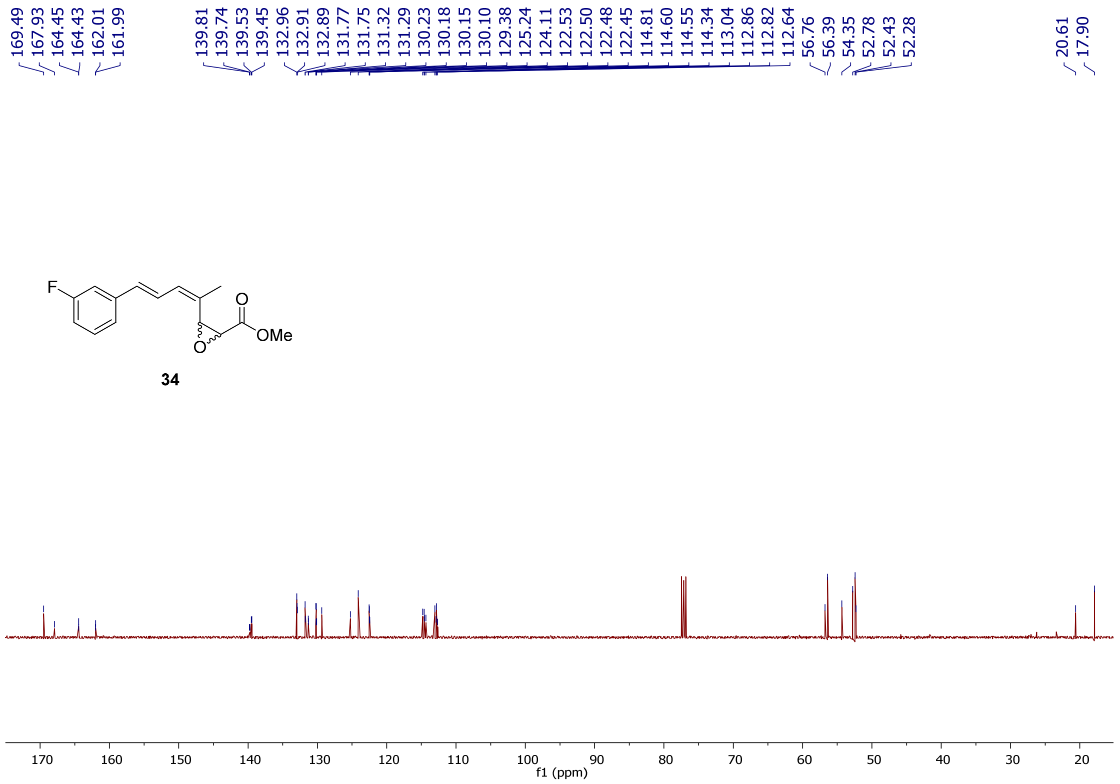

Figure S27. ^{1}H and ^{13}C NMR spectra of methyl (4*Z*,6*E*)-2,3-epoxy-7-(3-fluorophenyl)-4-methylhept-4,6-dienoate **34**.

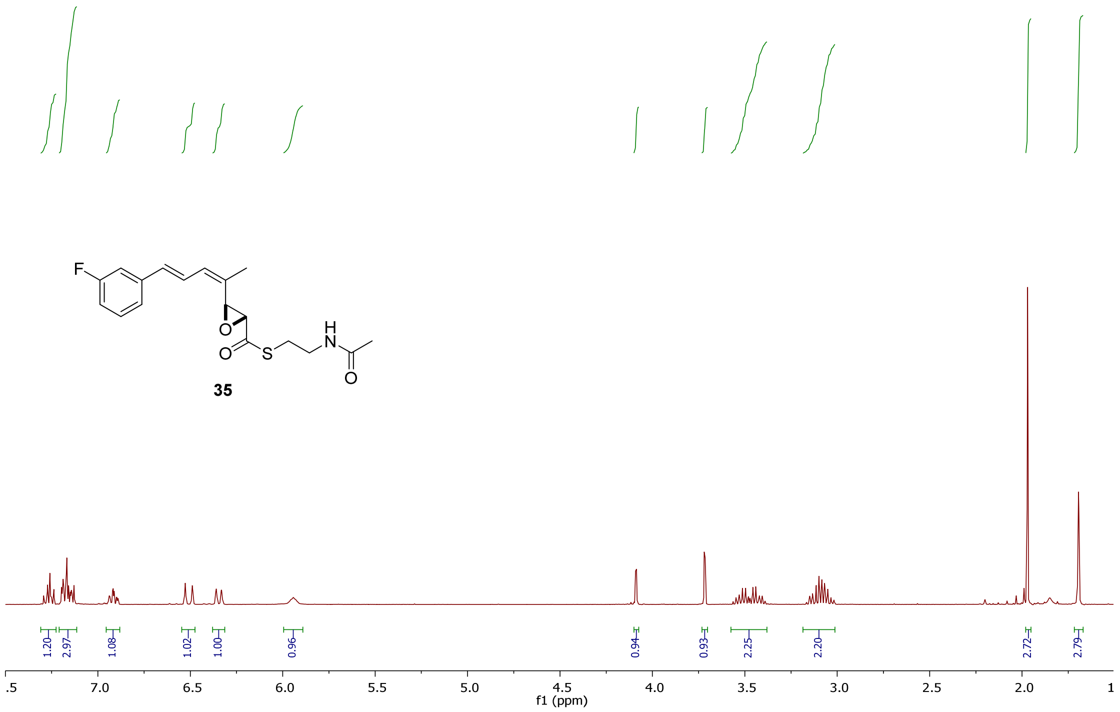

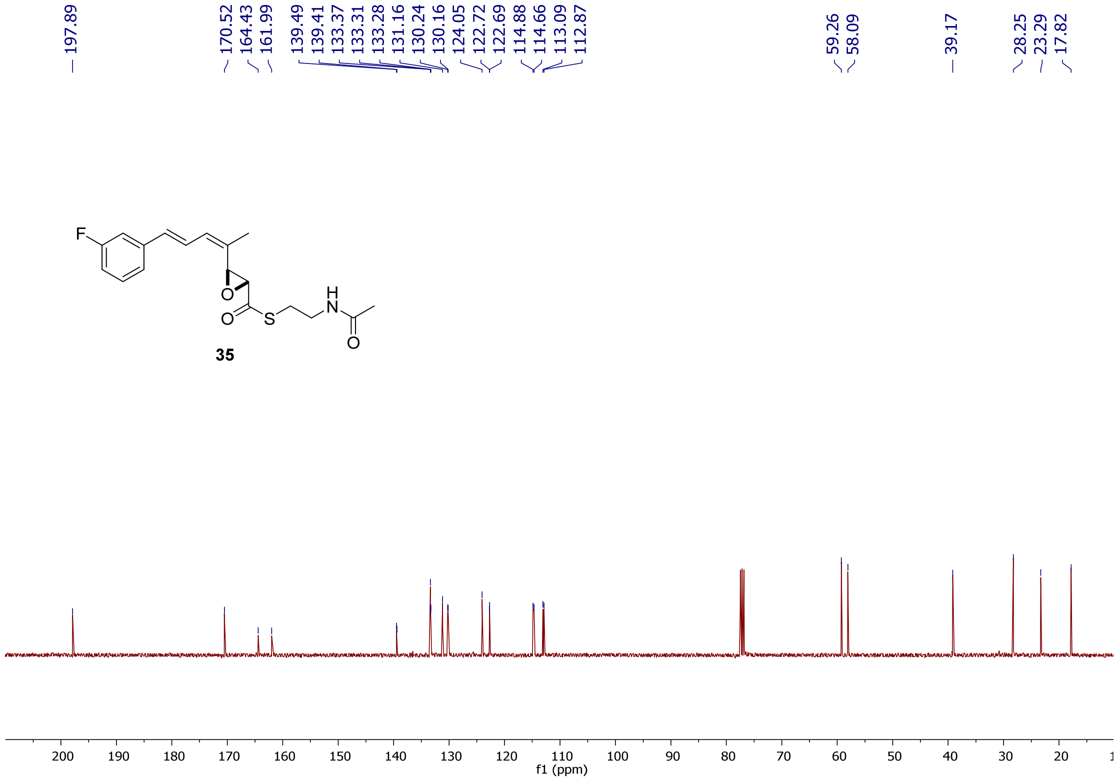

Figure S28. ^{1}H and ^{13}C NMR spectra of (4*Z*,6*E*)-2,3-Epoxy-7-(3-fluorophenyl)-4-methylhept-4,6-dienoic acid *N*-Acetyl cysteamine thioester **35**.

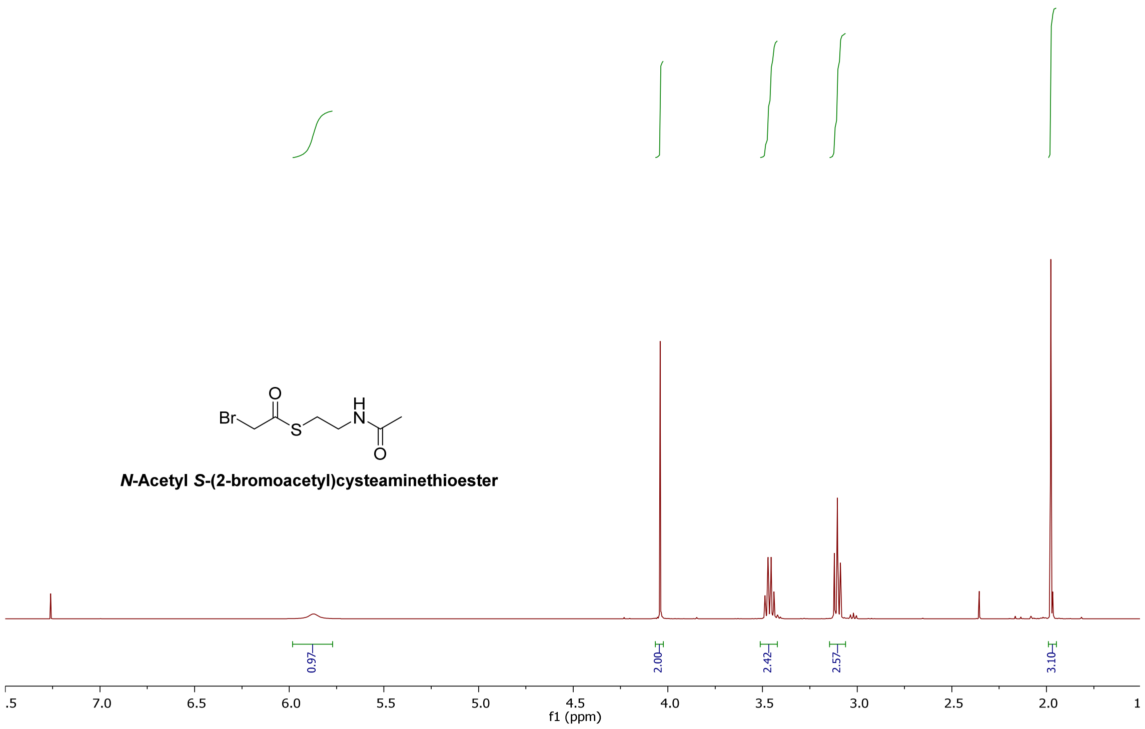

Figure S29. ^{1}H NMR spectrum of *N*-Acetyl *S*-(2-bromoacetyl)cysteaminethioester

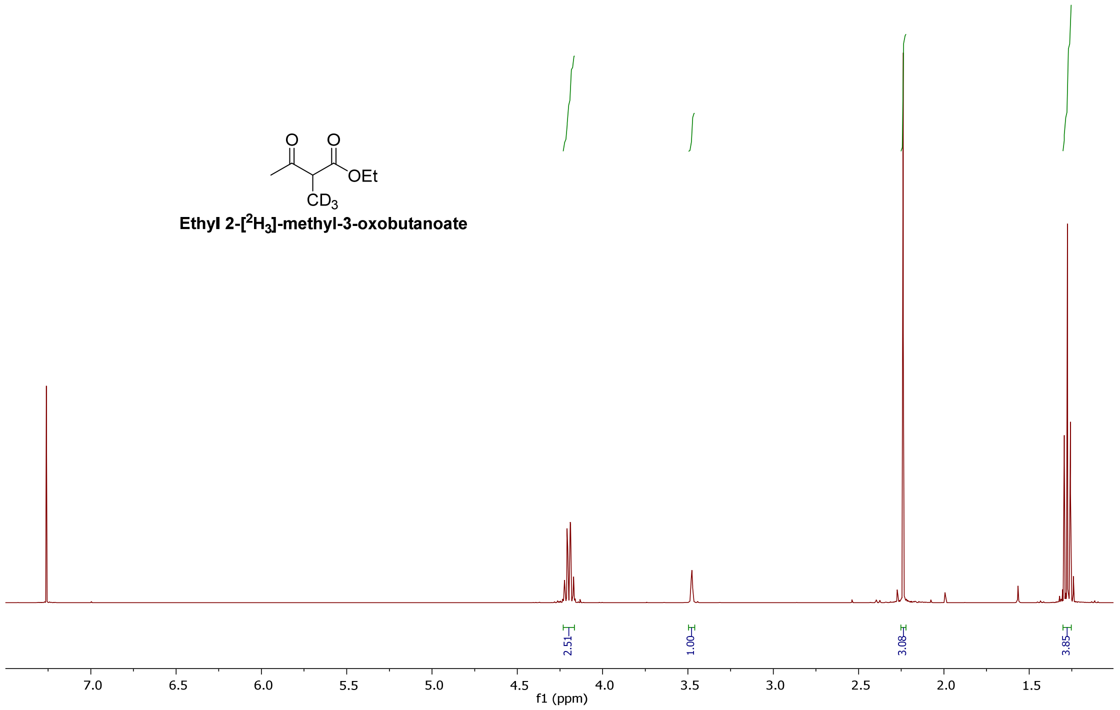

Figure S30. ^{1}H NMR spectrum of ethyl 2-[$^{2}H_3$]-methyl-3-oxobutanoate.

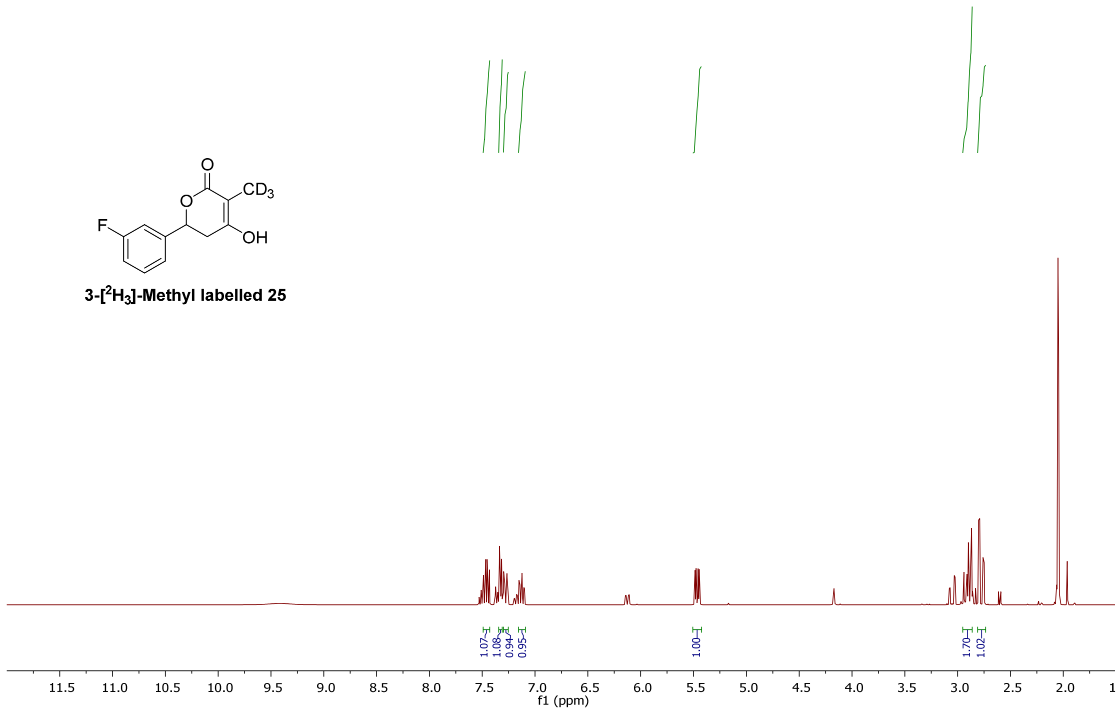

Figure S31. ^{1}H NMR spectrum of trideuterio-methyl **25**.

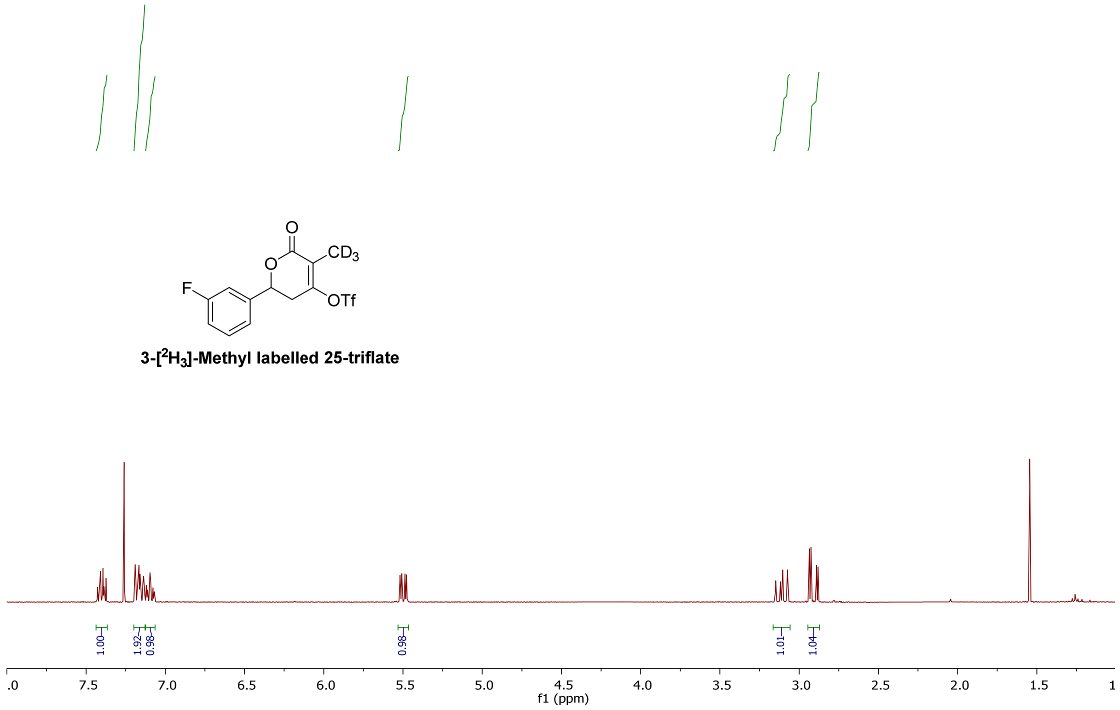

Figure S32. ^{1}H NMR spectrum of trideuterio-methyl **25** triflate.

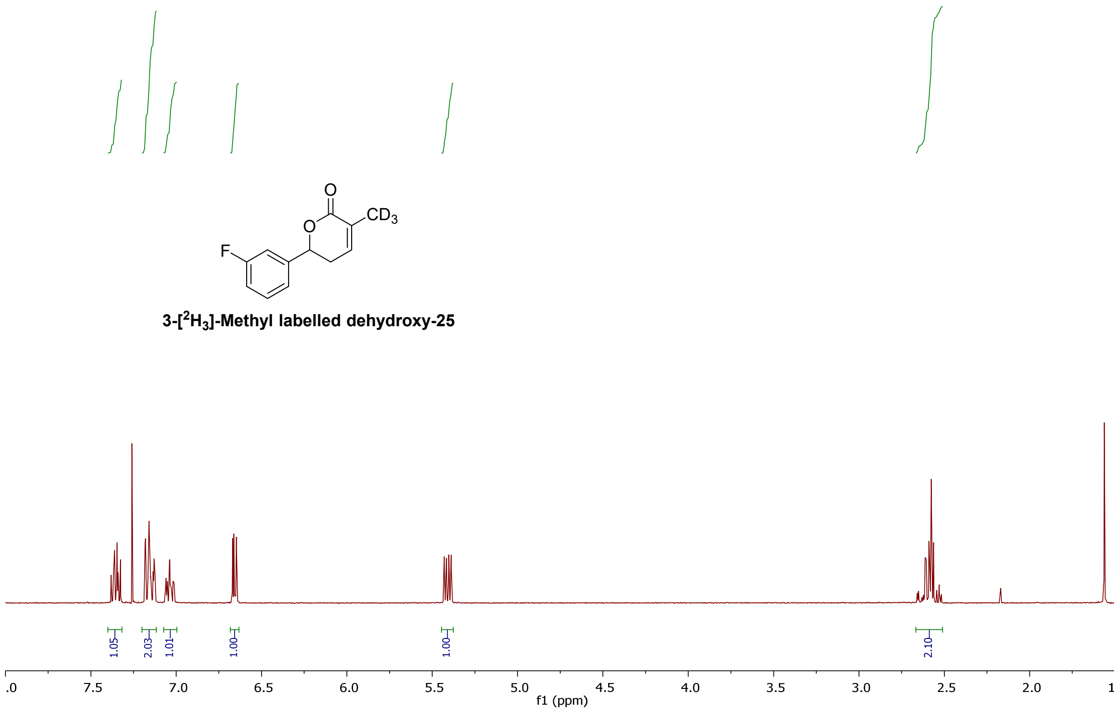

Figure S33. ^{1}H NMR spectrum of trideuterio-methyl dihydroxy-**25.**

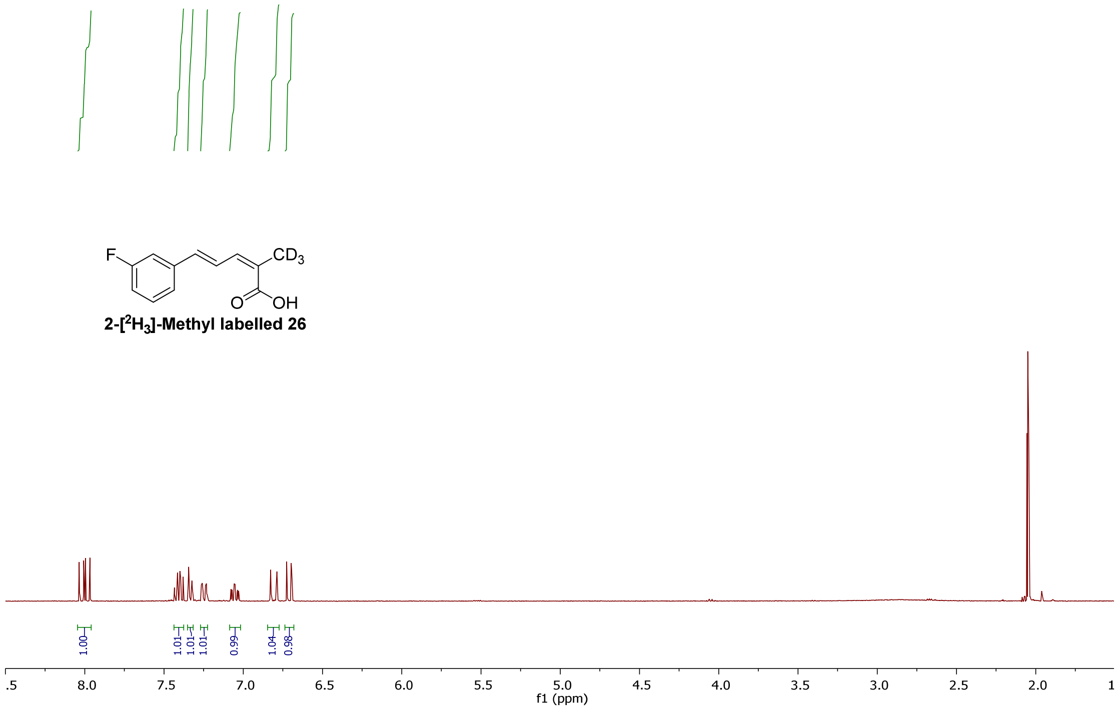

Figure S34. ^{1}H NMR spectrum of trideuterio-methyl **26.**

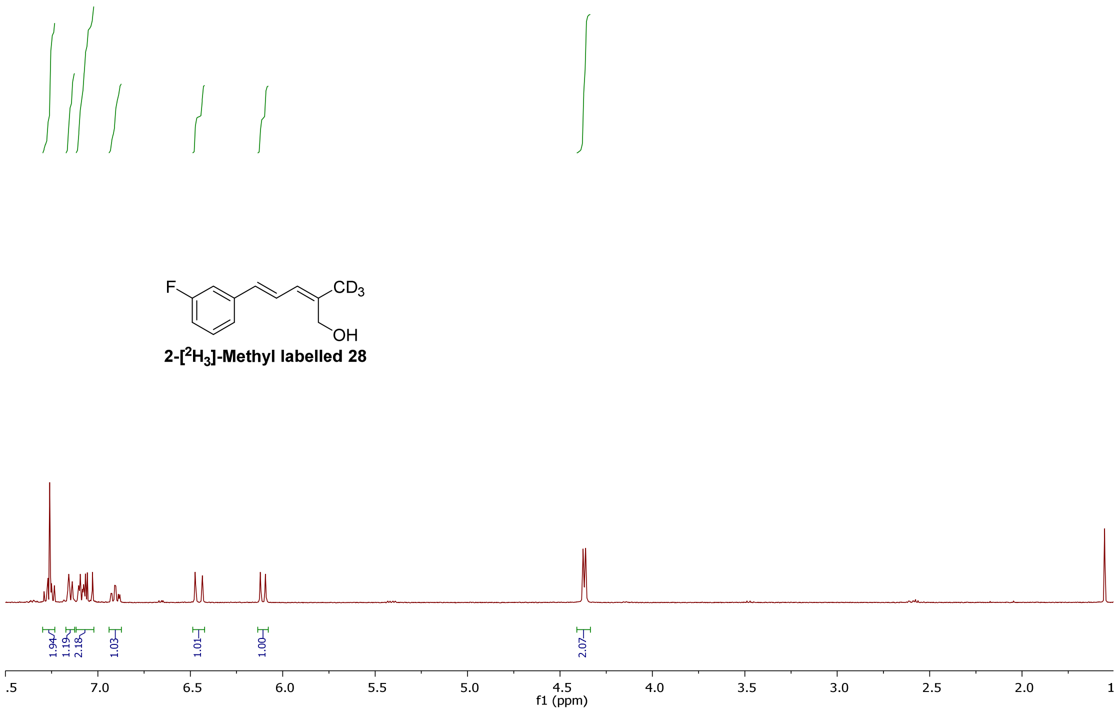

Figure S35. ^{1}H NMR spectrum of trideuterio-methyl **28.**

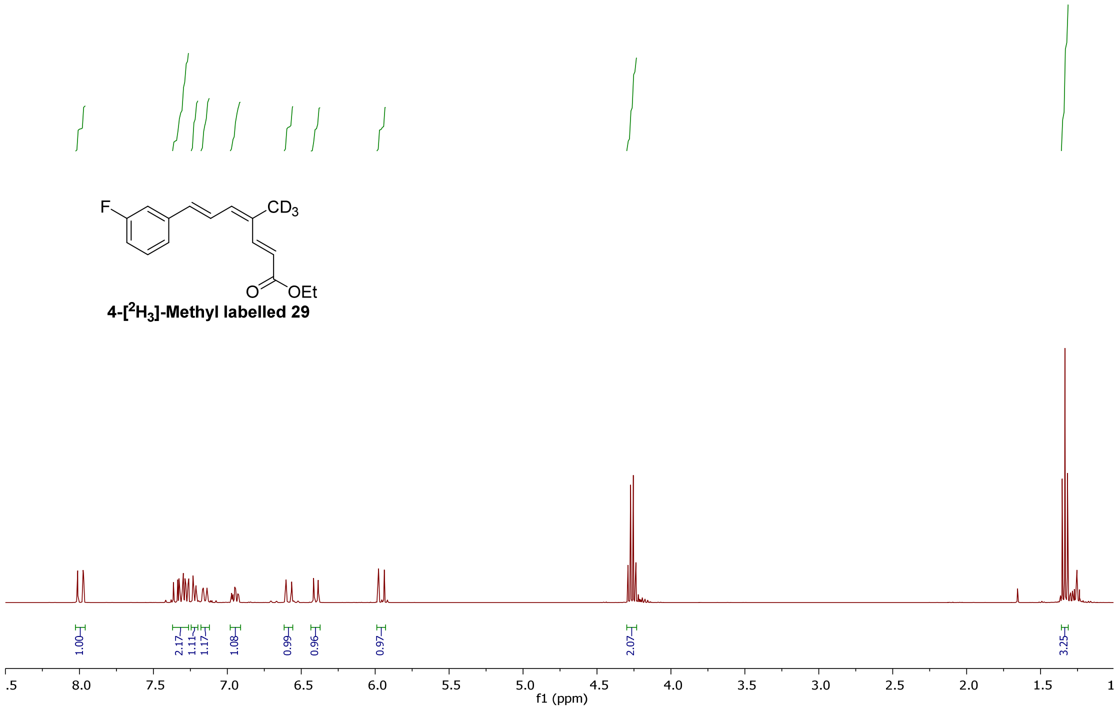

Figure S36. ^{1}H NMR spectrum of trideuterio-methyl **29**

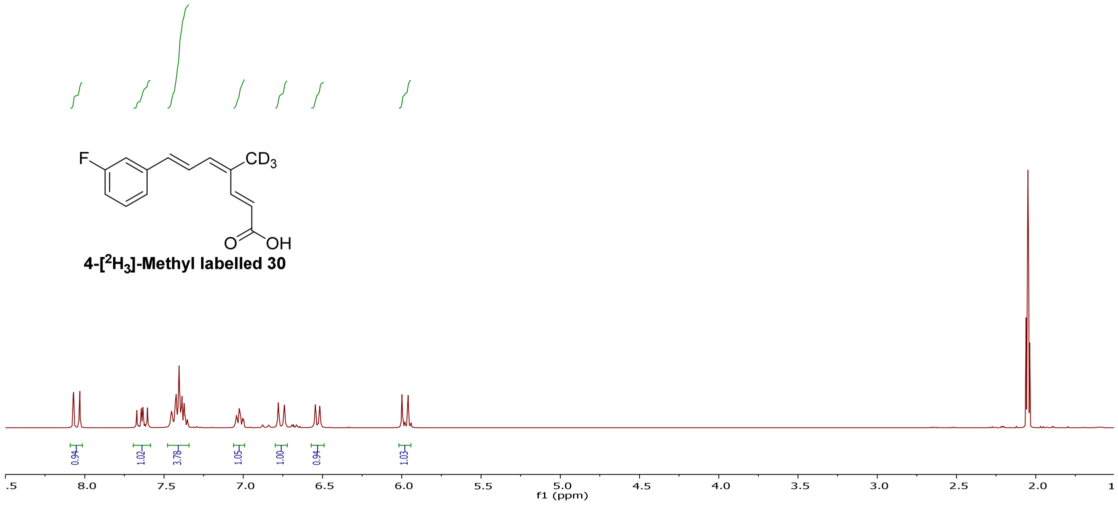

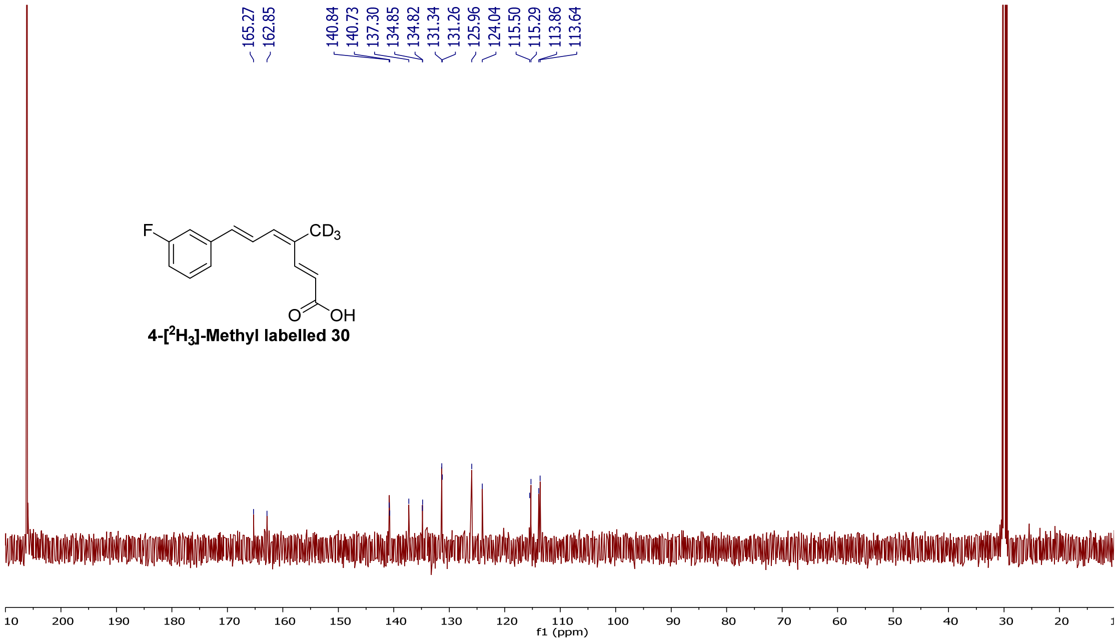

^{19}F-NMR

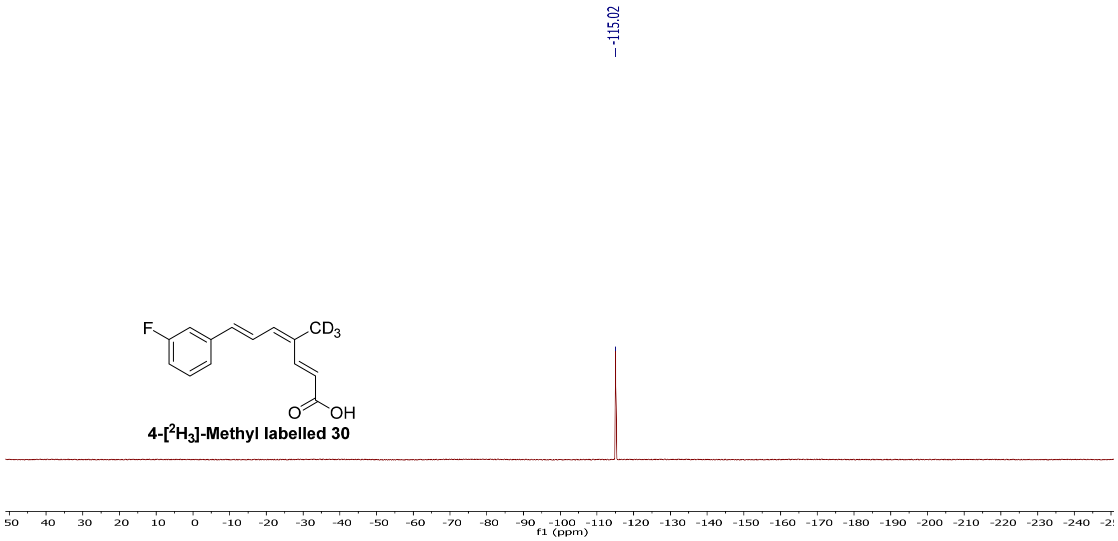

Figure S37. ^{1}H and ^{13}C and ^{19}F NMR spectra of trideuterio-methyl **30**

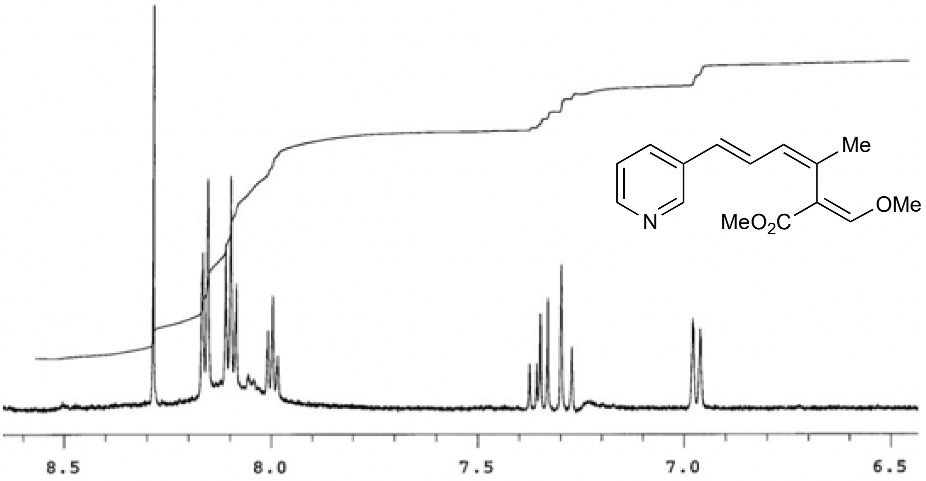

Fig**ure S38**. ^{1}H NMR spectrum of aromatic and olefinic region of 3-aza-strobilurin A **60**

---

[1] W. C. Still, M. Kahn, A. Mitra. *J. Org. Chem.*, 1978, **43**, 2923-2925.

[2] H. Keipour, T. Ollevier, *Org. Lett.*, 2017, **19**, 5736-5739.b

[3] G. Roblot, R. Wylde, A. Martin, J. Parello, *Tetrahedron*, 1993, **49**, 6381-6398.
